# Identification of key sequence features required for microRNA biogenesis in plants

**DOI:** 10.1038/s41467-020-19129-6

**Published:** 2020-10-21

**Authors:** Arantxa M. L. Rojas, Salvador I. Drusin, Uciel Chorostecki, Julieta L. Mateos, Belén Moro, Nicolas G. Bologna, Edgardo G. Bresso, Arnaldo Schapire, Rodolfo M. Rasia, Diego M. Moreno, Javier F. Palatnik

**Affiliations:** 1 IBR (Instituto de Biología Molecular y Celular de Rosario), CONICET and Universidad Nacional de Rosario, Rosario, 2000 Argentina; 2 Área Física, Departamento de Química-Física, Facultad de Ciencias Bioquímicas y Farmacéuticas, Universidad Nacional de Rosario, S2002LRK Rosario, Santa Fe, Argentina; 3 Área Biofísica, Facultad de Ciencias Bioquímicas y Farmacéuticas, Universidad Nacional de Rosario, Suipacha 531, S2002LRK Rosario Santa Fe, Argentina; 4 Instituto de Química de Rosario (CONICET-UNR), Suipacha 570, S2002LRK Rosario Santa Fe, Argentina; 5 Área Química General e Inorgánica, Departamento de Química-Física, Facultad de Ciencias Bioquímicas y Farmacéuticas, Universidad Nacional de Rosario, Suipacha 531, S2002LRK Rosario Santa Fe, Argentina; 6 Centro de Estudios Interdisciplinarios, Universidad Nacional de Rosario, Rosario, 2000 Argentina; 7 Present Address: Barcelona Supercomputing Centre (BSC-CNS), Barcelona, (08034) Spain; 8 Present Address: Institute for Research in Biomedicine (IRB), The Barcelona Institute of Science and Technology, Barcelona, (08028) Spain; 9 Present Address: Instituto de Fisiología, Biología Molecular y Neurociencias (IFIBYNE), CONICET-UBA, Buenos Aires, (1428) Argentina; 10 Present Address: Centre for Research in Agricultural Genomics (CRAG), CSIC-IRTA-UAB-UB, Campus UAB, Barcelona, (08193) Spain

**Keywords:** Non-coding RNAs, RNAi, Plant sciences, Plant molecular biology

## Abstract

MicroRNAs (miRNAs) are endogenous small RNAs of ∼21 nt that regulate multiple biological pathways in multicellular organisms. They derive from longer transcripts that harbor an imperfect stem-loop structure. In plants, the ribonuclease type III DICER-LIKE1 assisted by accessory proteins cleaves the precursor to release the mature miRNA. Numerous studies highlight the role of the precursor secondary structure during plant miRNA biogenesis; however, little is known about the relevance of the precursor sequence. Here, we analyzed the sequence composition of plant miRNA primary transcripts and found specifically located sequence biases. We show that changes in the identity of specific nucleotides can increase or abolish miRNA biogenesis. Most conspicuously, our analysis revealed that the identity of the nucleotides at unpaired positions of the precursor plays a crucial role during miRNA biogenesis in *Arabidopsis*.

## Abstract (web summary)

The secondary structure of miRNA precursor sequences is known to affect processing by DICER-like proteins. Here Rojas et al. show that additional sequence features also play a regulatory role in plants with nucleotide identity at unpaired positions substantially impacting processing efficiency.

## Introduction

MicroRNAs (miRNAs) are one class of small RNAs that perform essential functions as post-transcriptional regulators of gene expression in animals and plants. Plant miRNAs are transcribed as longer transcripts by RNA polymerase II, which are capped and polyadenylated (reviewed in refs. ^1,2^). These primary transcripts harbor an imperfect foldback structure with the miRNA embedded in one of its arms. In plants, all the processing steps necessary to release the mature miRNA from the precursor are carried out in the nucleus by a complex formed by DICER-LIKE1 (DCL1) and accessory proteins, such as HYPONASTIC LEAVES1 (HYL1) and SERRATE (SE) (reviewed in refs. ^1,2^). DCL1 performs cuts in the foldback at positions flanking the miRNA, releasing two paired RNA strands of ~21 nt with two nt 3′ overhangs, the miRNA/miRNA* duplex. One of these small RNAs, generally the miRNA, becomes incorporated into an ARGONAUTE (AGO) protein where it provides sequence specificity to identify target RNAs (reviewed in refs. ^3,4^). The *Arabidopsis* genome encodes ten AGO proteins and the miRNA pathway usually recruits AGO1 that preferentially binds small RNAs with 5′ U, which is the most common nucleotide at the 5′-end of plant miRNAs^3,4^.

The precise excision of the mature miRNA from the precursor is a key aspect of miRNA biogenesis, and relevant for the specific regulation of target RNAs. Many evolutionarily conserved miRNAs regulate transcription factors with important roles in plant development and hormone signaling, hence, mutations in the miRNA biogenesis machinery lead to important defects in plant growth and/or lethality^1^. Plant miRNA precursors are largely variable in size and shape, yet they are accurately processed to release rather specific miRNA sequences^5–7^. Previous work has identified structural determinants present in the precursors that guide the miRNA processing machinery to produce specific cuts along the precursor sequence^5–12^.

Numerous precursors have a 15–17 bp dsRNA segment above an inner loop and below the miRNA/miRNA* duplex, which guides a first cleavage by DCL1 at the proximal part of the precursor (base-to-loop processing)^7,9–11^. Other precursors have a dsRNA segment above the miRNA/miRNA* duplex and below a small terminal loop^5,12–14^. In these precursors, the processing machinery produces a first cut that releases the terminal loop and then continues towards the base of the precursor to release the miRNA/miRNA* duplex (loop-to-base processing)^5,13,14^. In either case, after a first cut is generated, DCL1 produces a subsequent cut ~21 bp away from the first one, releasing a miRNA/miRNA* duplex^7,15^.

Although previous work has shown the essential role of the precursor secondary structure for miRNA biogenesis, little is known about the importance of the precursor primary sequence. Here, by analyzing precursors from different plant species we found sequence biases at specific locations. Based on this information, we were able to increase or decrease the processing efficiency by introducing changes in the precursor sequence. Furthermore, our analysis revealed that the identity of nucleotides at mismatched positions along the precursor structure can play a significant role during miRNA processing. Most conspicuously, we found that C-C mismatches consistently impair miRNA biogenesis, and that they are absent from DCL1 cleavage sites.

## Results

### Identification of sequence biases in plant miRNA precursors

**Fig. 1:**
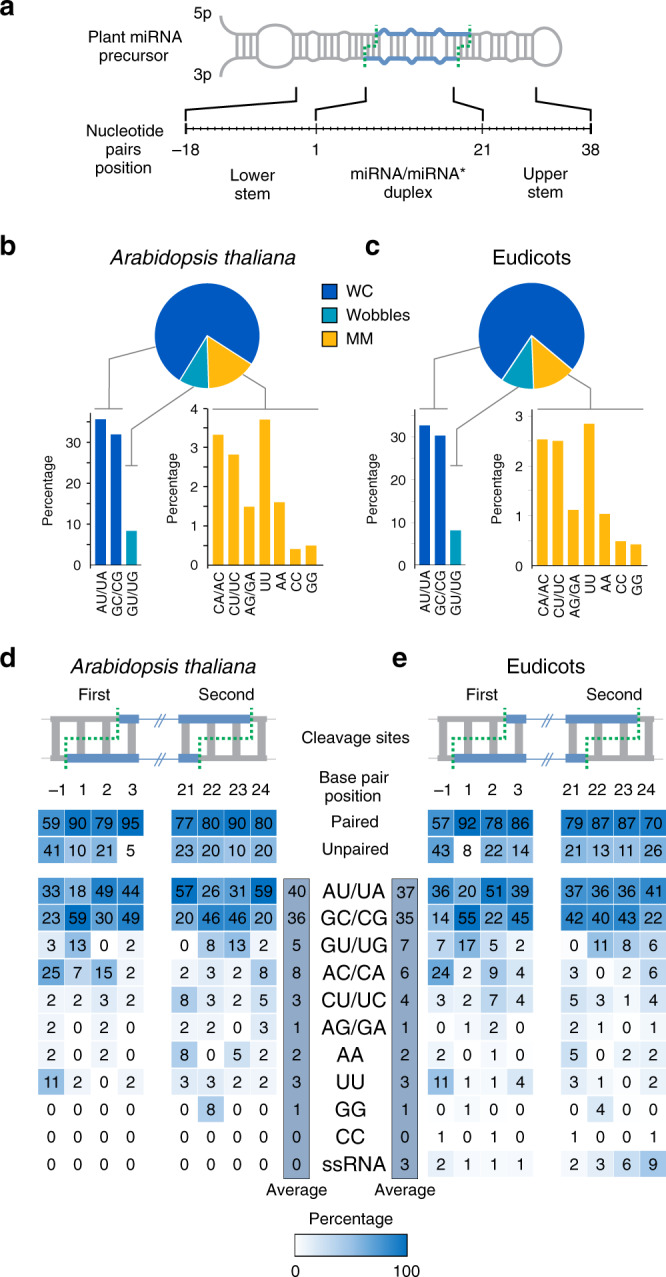
Frequency of nucleotide pairs in plant miRNA precursors. **a** Schematic representation of a plant microRNA precursor. The microRNA/microRNA* is indicated in blue, and the DCL1 cleavage site with dashed green lines. **b**, **c** Overall nucleotide pairs composition in conserved *MIRNA* precursors of *Arabidopsis thaliana* (**b**) and eudicots (**c**). Pie chart indicating the percentage of Watson–Crick pairs (WC), G-U wobbles pairs and mismatches (MM). Bars indicate the percentage of each pair. **d**, **e** Base pair composition at DCL1 cleavage sites of miRNA precursors from *Arabidopsis thaliana* (**d**) and eudicots (**e**). The first and second cleavage sites are indicated. Positions selected for each group of precursors are indicated in Supplementary Fig. 1. Numbers in each square indicate the percentage of each variant on each position.

To examine whether *MIRNAs* from *Arabidopsis* and other angiosperms show any nucleotide bias beyond the miRNA sequence, we analyzed miRNA precursors from 30 eudicot species (Source Data). First, we determined the frequency of Watson–Crick base pairs (WC), G-U wobbles, and mismatches (MM) in the secondary structure of *Arabidopsis* precursors, considering 56 pairs in length, therefore including the miRNA/miRNA* as well as regions below and above this duplex (Fig. 1a). We found that ~65% of the positions were paired, which is consistent with the structured nature of the miRNA precursors (Fig. 1b, c). Still, the G-U wobble (~8%) was less frequent than a A-U or G-C canonical Watson–Crick base pair (>30% each one) (Fig. 1b, c). We found similar proportions when we analyzed the *MIRNAs* in different eudicots (Fig. 1b, c).

Despite mismatches being less frequent than paired positions, we were able to analyze 10273 mismatched positions belonging to 1681 precursors of 30 eudicot species. Interestingly, we observed variation in the identity of the nucleotides composing the mismatches. The most common pair was U-U present in ~3% of the positions analyzed. On the other hand, C-C and G-G mismatches were present in <0.5% of the cases (Fig. 1b, c).

Next, we focused on the secondary structure of the two DCL1 cleavage sites flanking the miRNA/miRNA* duplex, which correspond to positions −1, 1, 2, 3 and 21, 22, 23, 24 for the first and the second cleavage site (Fig. 1d, e, Supplementary Fig. 1) (Source Data). Watson–Crick base pairs were the most frequent interactions, especially at positions 1, 3, and 23 (Fig. 1d, e). In contrast to these positions, position −1 was unpaired in ~40% of the precursors (Fig. 1d, e). The preferred mismatch at this position was an A-C/C-A with an overall frequency of 25% (*Arabidopsis*) and 24% (eudicots) (Fig. 1d, e). Interestingly, C-C mismatches, which were infrequent in the overall precursor structures (Fig. 1b, c), were absent from DCL1 cleavage sites (Fig. 1d, e). These biases were not explained by the frequencies of the individual nucleotides, as they were similar to each other (Supplementary Table 1).

As a comparison, we analyzed the composition of the cleavage sites of human miRNA precursors. All the positions corresponding to the first cleavage site were rather similar in human precursors with ~70% of Watson–Crick base pairs (Supplementary Fig. 2). No obvious bias in the preference of the nucleotide pairs was observed, and C-C mismatches were present at similar frequencies as other mismatch variants (Supplementary Fig. 2), suggesting that the features we found were plant specific.

### A single-nucleotide polymorphism increases the processing efficiency of *MIR172A*

**Fig. 2:**
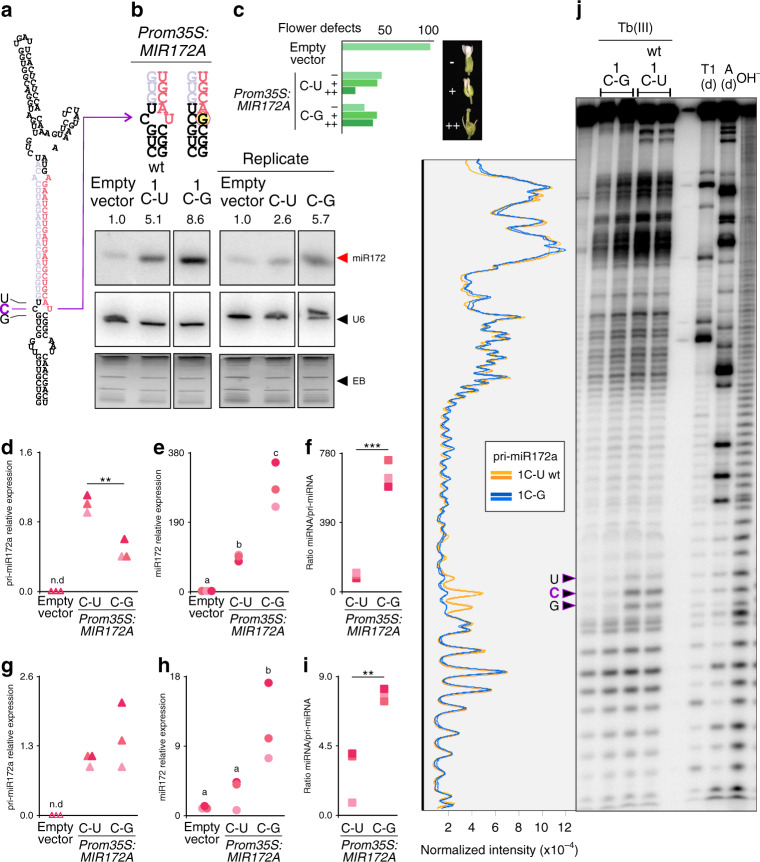
A single-nucleotide modification enhances miRNA biogenesis in vivo. **a** Predicted pri-miR172a foldback structure. The miRNA is shown in pink and the miRNA* in light purple. Letters on the left correspond to the nucleotides indicated with arrows in **j**. **b** Small RNA blots of primary transgenic inflorescences overexpressing *MIR172A* wt (1C-U) and mutated, 1C-G. Numbers above the gel indicate signal intensity normalized to U6 signal and relative to plants expressing the empty vector. The ethidium bromide (EB) staining of each gel is shown at the bottom. Blots were cropped to remove irrelevant lanes; see Supplementary Fig. 11a for uncropped images of the gels. **c** Phenotypes of plants overexpressing *MIR172A* wt (1C-U) and mutated (1C-G). Percentage of plants with no (−), moderate (+), and strong (++) flower defects. A representative flower for each phenotype is indicated in the inset. **d**–**i** Accumulation of pri-miRNA (**d**, **g**) and miRNA (**e**, **h**) as well as ratios between miRNA and pri-miRNA (**f**, **i**) in seedlings (**d**–**f**) or inflorescences (**g**–**i**) expressing the empty vector, wt *MIR172A* (1C-U), or mutated 1C-G as determined by RT-qPCR. Each sample indicated by different shades of pink corresponds to a pool of at least ten independent transgenic plants. Asterisks in **d**, **f**, and **i** indicate statistically significant differences according to Student *t* test (two-tailed), *p* < 0.01 (**) and *p* < 0.001 (***). Different letters in **e** and **h** indicate statistically significant differences, according to ANOVA followed by Tukey’s multiple comparison test (*p* < 0.05). n.d., not detected. **j** Denaturing polyacrylamide gel of Terbium induced autohydrolysis of pri-miR172a wt (1C-U) and mutated (1C-G). From right to left: in vitro transcribed pri-miR172a incubated with Tb (III), C-G and wt (C-U) respectively; T1 (d)T1 RNase in denaturing conditions; A(d): RNAse A in denaturing conditions; OH^−^: alkaline hydrolysis. Two independent reactions were loaded for each treatment. The intensity profile is depicted on the left. Arrowheads indicate bands with different intensity in wt and the 1C-G mutant variant. See Supplementary Fig. 12 for uncropped image of the gel.

Of the different nucleotide pairs forming the first DCL1 cleavage site, the G-C/C-G pair at position 1 had the highest frequency, being present at 59% and 55% of the precursors of *Arabidopsis* and eudicots, respectively (Fig. 1d, e). Furthermore, this position is paired in 90% or more of the plant miRNA precursors. Exceptions to this rule are the *Arabidopsis* *MIR827* and *MIR172A* precursors (Supplementary Fig. 3). We analyzed the importance of the pairing at position 1 in *MIR172A*, which causes obvious phenotypes after overexpression, namely early flowering time and flower-patterning defects, that quantitatively correlate with the mature miRNA levels^10,11,16,17^. *MIR172A* harbors a C-U mismatch at position 1 (Fig. 2a, Source Data), so we introduced a single change by site-directed mutagenesis in the *MIR172A* precursor and generated a C-G variant.

We analyzed miR172 levels in inflorescences of primary transgenic (T1) plants overexpressing the wild-type (wt) and the C-G variant precursor from the *35S* viral promoter using small RNA blots (Fig. 2b). Overexpression of the *MIR172* C-G mutant variant precursor resulted in >60% increase in the accumulation of mature miRNA with respect to control inflorescences transformed with wt *MIR172* precursor (Fig. 2b). Although both precursors caused a strong acceleration of flowering (Supplementary Fig. 4a), we found that the C-G variant produced stronger flower-patterning defects (Fig. 2c), as expected from the higher levels of mature miRNA. To further validate these results, we determined the levels of pri-miR172 and mature miR172 by RT-qPCR in seedlings (Fig. 2d, f) and inflorescences (Fig. 2g, i). Overall, we observed that the *MIR172* C-G mutant variant accumulated higher levels of miR172 than the wt *MIR172* C-U precursor (Fig. 2e, h), whereas the levels of pri-miR172 were similar or lower (Fig. 2d, g). In consequence, the ratio miR172/pri-miR172 was consistently higher for the *MIR172* C-G mutant variant compared with the wt *MIR172* C-U (Fig. 2f, i). The *MIR172* C-G variant accumulated slightly more miR172* than the wt precursor, but the difference was not statistically significant (Supplementary Fig. 4b).

The change C-U to C-G is located in a structured region of the *MIR172A* precursor (Fig. 2a). To test whether this single change affects the structure of the region, we probed the precursor using Terbium (Tb III), which induces the cleavage of RNA in a sequence-independent manner preferring flexible single-stranded and non-Watson–Crick base-paired regions^18,19^. Probing the two precursors with Tb (III) showed that the C-G variant reduced the flexibility of the precursor (Fig. 2j). Most conspicuously, the analysis revealed that three phosphodiester bonds in the vicinity of the mutation were selectively stabilized in the *MIR172A* C-G mutant precursor (Fig. 2j), suggesting a further stabilization of the base pairs near the mutation. Interestingly, *MIR172A* has been shown to be the most active member of the miR172 family in *Arabidopsis*^16,17^. Our results show that a single change can increase its processing efficiency and activity even further in vivo.

### The nucleotide identity at the DCL1 cleavage site significantly affects *MIR172* processing

**Fig. 3:**
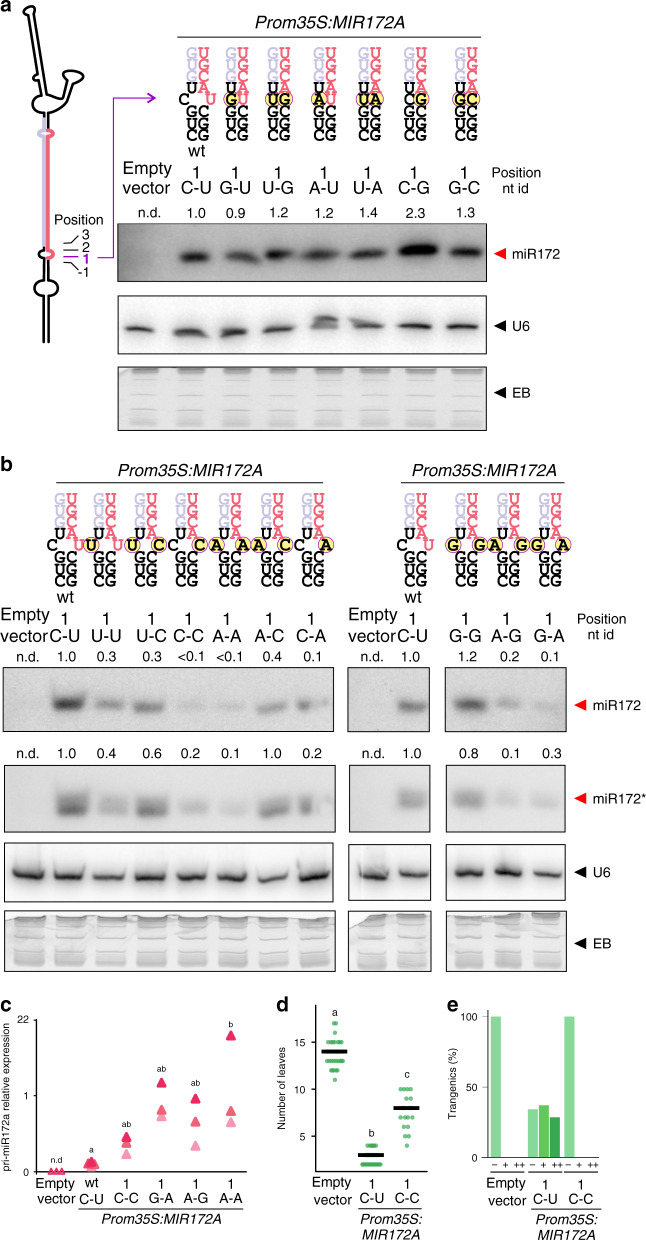
Nucleotide pair variants control miR172 biogenesis. **a**, **b** *MIR172A* was modified for all the nucleotide pair variants in position 1 of the precursor. Seedlings expressing all the possible pair variants in position 1 of the precursor were analyzed by small RNA blot for miR172. Numbers above gels indicate signal intensity normalized to U6 signal and relative to the wt precursor signal (1C-U). On **a**, a schematic representation of *MIR172A* is depicted, showing the position of the base pair modification. In **a**, **b** above each gel, a portion of the precursor is included, with the specific nucleotides modified indicated with a yellow circle. Bottom panel in **b**, a small RNA blot for miR172a*. The ethidium bromide (EB) staining of each gel is shown at the bottom. Blot **b** was cropped to remove irrelevant lanes; see Supplementary Fig. 11b–d for uncropped images of the gels. See Supplementary Figs. 5 and 6 for biological replicates. **c** Pri-miRNA quantification by RT-qPCR of seedlings expressing *MIR172A* unpaired variants: wt, 1C-C, 1 G-A, 1 A-G and 1 A-A. Different letters indicate statistically significant differences (ANOVA followed by Tukey’s multiple comparison test (*p* < 0.05)). n.d.: not detected. **d**, **e** Phenotypes of plants overexpressing *MIR172A* wt (1C-U) and mutated (1C-C) variant. Note the small effect of the mutated *MIR172A* C-C on flowering time (**d**) and flower-patterning defects (**e**) (see Fig. 2c for phenotypes references). Different letters in **d** indicate statistically significant differences (Kruskal–Wallis multiple comparison test, *p* < 0.05).

Next, we systematically analyzed the importance of the base pair composition at position 1 of the *MIR172A* pri-miRNA by generating mutant variants with the 16 different possible combinations of nucleotides (Fig. 3). These precursors were cloned under the control of the *35* *S* viral promoter and transformed into plants. We first compared the wild-type precursors with mutant variants harboring closed pairs, either with canonical Watson–Crick base pairs or G-U wobble (Fig. 3a). All these variants accumulated miRNA levels similar to the wild-type precursor, with the exception of the *MIR172A* C-G variant that accumulated the highest levels of the small RNA (Fig. 3a and Supplementary Fig. 5a). We also determined mature miR172 and pri-miR172 by RT-qPCR in these mutants and confirmed that the MIR172 C-G variant had the highest ratio miR172/pri-miR172 (Supplementary Fig. 5b–d).

Differently to the results obtained with *MIR172A* mutants that harbored closed pairs, we found that precursors with mismatches accumulate largely variable amounts of mature miRNA levels, which can decrease >10-fold depending on the identity of nucleotides forming the mismatch (Fig. 3b and Supplementary Fig. 6). Interestingly, the A-C and C-C mismatch variants, which generate miRNAs of identical sequences, accumulated largely different levels of miRNAs (Fig. 3b). Although the A-C mutant accumulated nearly half the levels of mature miRNA with respect to the wild-type precursor, the levels of miR172 were barely detected in the C-C mismatch variant (Fig. 3b and Supplementary Figure 6). Furthermore, the mutant variants A-A, A-G, and G-A accumulated ~10% mature miRNA levels with respect to the C-U wt *MIR172A* (Fig. 3b).

We determined the levels of miR172* (Fig. 3b, lower panel) and found a similar pattern of accumulation for both guide and passenger miR172 (Fig. 3b, upper panel), suggesting that differences in miR172 levels were not caused by preferentially loading miR172* into an AGO complex. We analyzed the pri-miRNA levels by RT-qPCR of the mutants that failed to accumulate miR172 and found that the mutant precursors accumulate larger amounts of primary transcripts (Fig. 3c). These results indicate that these mutants were affected at the level of the precursor processing. In good agreement with the low miR172 levels, overexpression of the *MIR172* C-C variant caused only a minor effect on flowering time and no defects on the flower patterning (Fig. 3d, e).

### C-C mismatches systematically affect plant miRNA processing

**Fig. 4:**
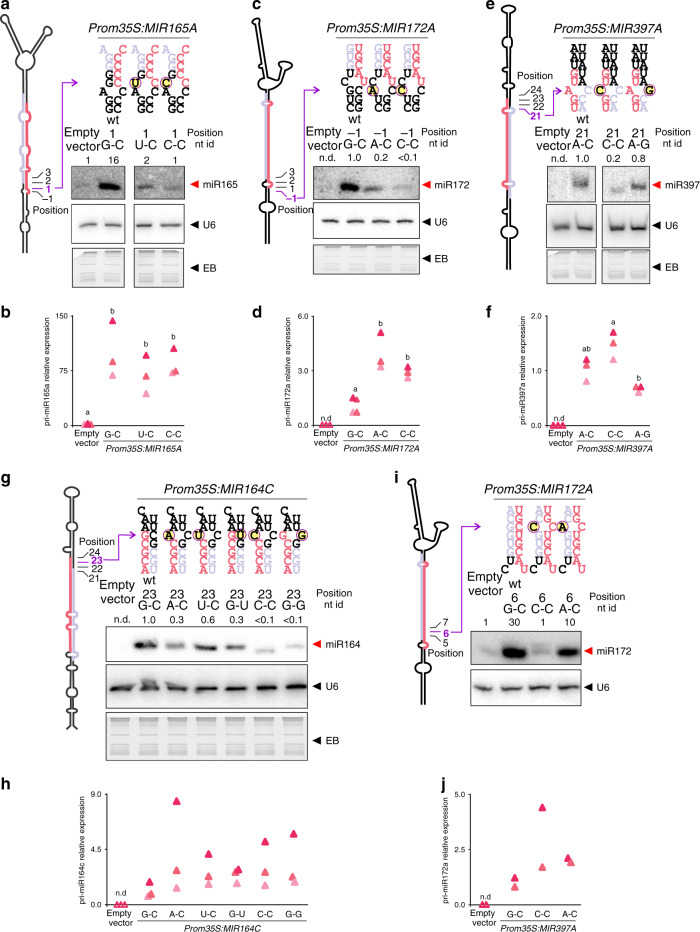
miRNA biogenesis is strongly affected by the nucleotide identity at the precursor unpaired positions. Several miRNA precursors where modified and overexpressed in plants. Schemes of miRNA precursors are included to the left in each panel, with the miRNA in pink and miRNA* in light purple. Seedlings expressing the *MIRNA* variants were analyzed by small RNA blots (**a**, **c**, **e**, **g**, **i**) and RT-qPCR (**b**, **d**, **f**, **h**, **j**). Numbers above gels indicate signal intensity normalized to U6 signal and relative to empty vector (**a**, **i**) or wt precursor on each gel (**c**, **e**, **g**). The ethidium bromide (EB) staining of each gel is shown below the blots. See Supplementary Fig. 7 for biological replicates and Supplementary Fig. 11e–i for uncropped gel images. The pri-miRNA levels were determined in three biological replicates by RT-qPCR and indicated below the blots. Different letters indicate significant differences (ANOVA followed by Tukey’s multiple comparison test (*p* < 0.05)). n.d., not detected. **a**, **b** *MIR165A* wt (1 G-C) and 1 U-C and 1 C-C mutant variants. **c**, **d** *MIR172A* wt (−1 G-C), and -1 A-C and −1 C-C mutant variants. **e**, **f** *MIR397A* wt (21 A-C) and 21 C-C and 21 A-G mutant variants. **g**, **h** *MIR164C* wt (23 G-C) and 23 A-C, 23 U-C, 23 G-U, 23 C-C, and 23 G-G mutant variants. **i**, **j** *MIR172A* wt (6 G-C) and 6 C-C and 6 A-C mutant variants.

As the identity of the unpaired nucleotides at position 1 of *MIR172A* significantly affected miRNA biogenesis, we decided to test their importance in other precursors and positions (Fig. 4). At position −1, *MIR165A* has a mismatch followed by a Watson–Crick G-C pair at position 1 (Fig. 4a). Introducing a mutation at position 1 that generated two continuous mismatches affected the biogenesis of miR165a (Fig. 4a, b and Supplementary Fig. 7a, b), in good agreement with previous work highlighting the importance of the secondary structure in *MIRNA* processing^9–11^. However, the C-C mismatch at position 1 reduced even further the biogenesis of miR165a than the U-C mismatch (Fig. 4a and Supplementary Fig. 7a, b). We also generated two new mutants in *MIR172A*, an A-C and C-C mismatch at position −1. Although both mismatches affected miR172 biogenesis, the C-C mismatch had a stronger effect than the A-C variant (Fig. 4c and Supplementary Fig. 7e, f). These results confirm the deleterious impact of C-C mismatches in the first DCL1 cleavage site.

Next, we analyzed mutations in the second DCL1 cleavage site. The *MIR397A* precursor has an A-C mismatch at position 21 (Fig. 4e). We introduced C-C and A-G mismatches and observed that the C-C was detrimental for miR397 biogenesis while the A-G variant was not (Fig. 4e and Supplementary Fig. 7c, d). *MIR164C* has a G-C pair at position 23, and we generated five variants by site-directed mutagenesis, A-C, U-C, G-U, C-C, and G-G (Fig. 4g, Supplementary Fig. 7g). We observed a broad distribution of miR164 accumulation depending on the identity of nucleotides (Fig. 4g, Supplementary Fig. 7g), similar to what we observed previously for miR172 (Fig. 3). We observed that C-C and G-G mutants accumulated much less miR164 than A-C, U-C, and G-U variants (Fig. 4g and Supplementary Figure 7g).

As expected, we observed a correlation between the miRNA levels generated by the different mutant precursors and the strength of the developmental defects (miR165, miR164) or the target transcript levels (miR397) (Supplementary Fig. 8). In addition, all the *MIRNA* mutants that failed to accumulate substantial levels of the mature miRNA expressed primary transcripts at similar or higher levels than their cognate wild-type *MIRNAs* (Fig. 4b, d, f, h), demonstrating that the low levels of the mature miRNAs were not caused by a poor expression of their precursors.

Overall, the results show that the identity of unpaired nucleotides in the miRNA precursors has a strong effect on the accumulation of the mature miRNA in vivo (Figs. 3 and 4). Although the final impact caused by the unpaired nucleotides is likely affected by the precursor context, we found that C-C mismatches consistently impaired miRNA biogenesis. Contrarily, the biogenesis of precursors harboring A-C, U-U, and U-C mismatches is similar to those with WC pairs. Interestingly, the latter mismatches were the most frequent in miRNA precursors (Fig. 1).

Next, we studied the effect of the identities of mismatches in the miRNA/miRNA* duplex region. Introducing a mismatch at position 6 of miR172/miR172* to generate an A-C mismatch had a small effect on miR172 accumulation (Fig. 4i–j, Supplementary Fig. 7h–i and Supplementary Fig. 8d, e). We observed, however, a different effect after introducing a C-C mismatch variant at the same position. On the one hand it diminished miR172 accumulation (Fig. 4i and Supplementary Fig. 7h, i). On the other hand, it caused the generation of two small RNA variants from the *MIR172A* precursor (Fig. 4i). So, although C-C mismatches impair the processing quantitatively in all the cases analyzed so far, the mutation in the miRNA/miRNA* region affected the biogenesis qualitatively. It has been recently shown that the flexibility on miR168/miR168* interaction can be linked to miR168 variants of different lengths and sequences^20^. We inspected the miR168/miR168* duplex and found a C-C mismatch that is conserved in miR168/miR168* duplexes in 37 out of 38 precursors (Supplementary Fig. 9), so that conservation of this particular mismatch is also consistent with particular properties of the precursor processing.

### Molecular dynamics analysis revealed differences in the mismatch variants of *MIR172A*

**Fig. 5:**
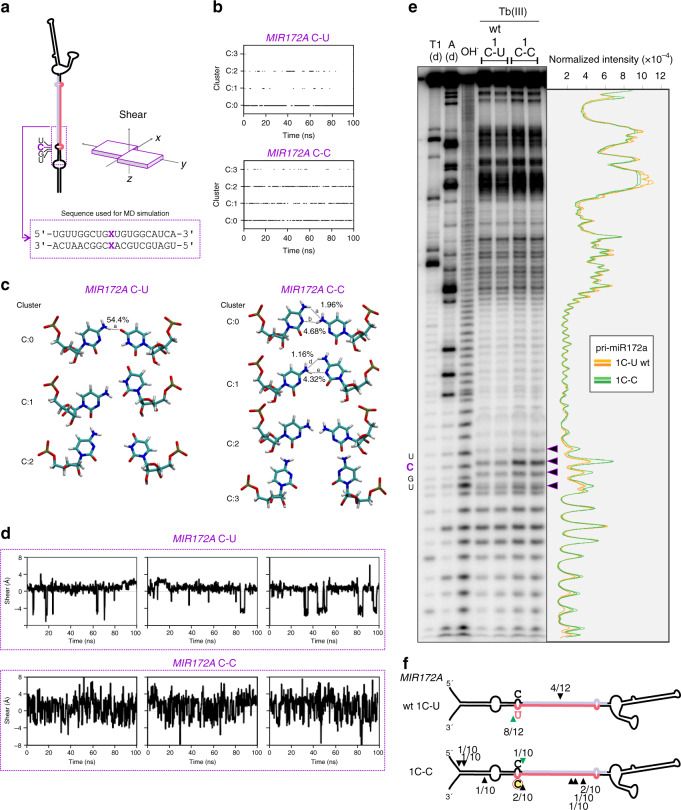
A C-C variant increases the flexibility of the precursor structure. **a** Schematic representation of *MIR172A* precursor. Letters on the left correspond to the nucleotides (nt) indicated with arrows in **e**. A dashed outline indicates the region used for molecular dynamics (MD) simulations, the sequence is showed below, “x” indicates the nucleotides modified for the simulation of each variant. The “shear” parameter (an in-plane shift of the bases within the base pair) is depicted on the right (movement of bases along the *x* axis). See Methods and Supplementary Data 1 for additional information. **b**–**d** Results of MD for *MIR172A* 1C-U (wt) and 1C-C variants in position 1. Clustering (**b**), nucleotide pair conformation (**c**), and shear parameter (**d**) are depicted. The percentage of simulation time that each hydrogen bond is formed is indicated next to the bonds in the conformational depictions (**c**), numbers indicating the conformations correspond to clusters in **b**. For shear parameter three replicates are shown. See Supplementary Data 1 for all other nucleotide variants. **e** Structural analysis of pri-miR172a with wt (C-U) and C-C variant at position 1. Denaturing polyacrylamide gel, from left to right: T1(d): T1 RNase in denaturing conditions; A(d): RNAse A in denaturing conditions; OH^−^: alkaline hydrolysis; pri-miR172a incubated with Tb(III), wt and C-C variants respectively; two independent reactions were loaded for each treatment. On the right, the intensity profile of Terbium reactions is depicted. Black arrowheads indicate bands with different intensity between wt and C-C variant. See Supplementary Fig. 12 for uncropped image of the gel. **f** *MIR172A* wt and 1C-C variant where analyzed through modified 5´RACE. Arrowheads and numbers indicate processing intermediates identified by sequencing and their frequency; green arrowheads indicate the expected DCL1 cleavage product.

The surprisingly different effects on miRNA biogenesis caused by the identity of nucleotides prompted us to study the system in more detail. We resorted to molecular dynamics (MD) simulations in order to obtain atomistic details about the influence of the base pair nature on the precursor behavior. To achieve this goal, the 16 variants for position 1 of a segment of the *MIR172A* precursor (Figs. 3 and 5a) were built in silico and subjected to 100 ns of MD simulations. A representative snapshot of the system was built for the wt variant, composed of the dsRNA, solvent box and NaCl (Supplementary Data 1). To check the stability of the *MIRNAs* through the simulations, the total axial bend of each model was calculated, which showed a similar stable behavior in all cases (Supplementary Data 1). These simulations were used to analyze in detail all the structural features of all the base pairs belonging to each nucleic acid chain (Supplementary Data 1 and 3). The structural parameter that showed the most distinct behaviors among the different mutant variants was the shear parameter, which represents the relative displacement between the bases on a plane perpendicular to the RNA axis (Fig. 5a–d, Supplementary Data 1).

Although canonical Watson–Crick base pairs as well as G-U wobbles showed a stable value of the shear parameter along the MD simulation (Supplementary Data 1), mismatch variants, displayed higher fluctuation of values. Some variants, such as C-U and A-C showed some fluctuation between stable values. Others, such as C-C had a higher fluctuation and no stable values at all (Fig. 5d). To better illustrate the different behavior of the nucleotide pair variants, we performed a clustering of the different conformations visited through the MD simulation (Fig. 5b, Supplementary Data 3) and an analysis of the formation and permanence of hydrogen bonds between the bases (Fig. 5c, Supplementary Data 3).

Canonical Watson–Crick base pairs and G-U wobbles had a single stable conformation, in agreement with the shear values. Variants such as G-G and A-C showed different positions for the bases along time (Supplementary Data 3), reflecting periodical changes between different conformations that are stabilized through hydrogen bonds (Supplementary Data 3). In contrast, analysis of the C-C mismatch showed constant movement between the different conformations, consistent with the lack of any stable conformation for this variant (Fig. 5b–d, Supplementary Data 1 and 3). Results for the C-C variant suggest significantly higher flexibility than in the wild-type precursor harboring a C-U mismatch. We also analyzed the variants at position 23 of *MIR164C* (Fig. 4g and Supplementary Fig. 10a) by MD. In good agreement, the shear parameter showed again an unstable behavior of the C-C pair (Supplementary Fig. 10b and Supplementary Data 2 and 3).

In order to confirm these results, we used Terbium (III) to probe the structure of the wild-type *MIR172A* precursor harboring C-U and the mutant with a C-C mismatch. Despite both precursors having a mismatch at position 1, we found that the *MIR172A* C-C precursor variant was more sensitive to Terbium (Tb III) than the wild-type precursor at the location of the C-C mismatch as well as at neighboring nucleotides (Fig. 5e), supporting our MD calculations, and demonstrating an increase of structural flexibility caused by the C-C mismatch.

In addition, we mapped the processing intermediates for *MIR172A* with a C-C mismatch and observed a random distribution of the cuts (Fig. 5f), in contrast to the wild-type precursor that harbored ~70% of the cuts flanking the miRNA/miRNA*. Altogether, the results show that the identity of the unpaired nucleotides confer different structural constraints that can be translated into an aberrant or altered miRNA precursor processing.

## Discussion

MiRNAs are processed from precursors harboring imperfect foldback structures. Many reports have demonstrated the importance of the precursor secondary structure during miRNA biogenesis^5–12^. These structural determinants are mostly represented by dsRNA segments of 15–17 bp located below or above the miRNA/miRNA*^5–12^. In addition, the identity of the 5′ nucleotide as well as mismatches in the miRNA/miRNA* region have been shown to be important for selection of the specific AGO protein^21–26^.

Here, we describe a new set of sequence features that are important to define a plant miRNA primary transcript. We identified certain preferred nucleotide pairs at the precursors’ cleavage site, which might contribute to the recognition and/or activity of the processing complex. However, an unexpected new finding is that the identity of the nucleotides located at mismatched positions of the imperfect dsRNA that serves as a miRNA precursor can have a deep impact on the processing efficiency. This is in turn reflected in the evolution of *MIRNAs*, as certain mismatches such as C-C and G-G are largely excluded from the precursor structures, especially from DCL1 cleavage sites. Although we did not find a similar bias in the nucleotide pairs at the cleavage sites of human precursors, a general analysis of the frequency of nucleotide pairs in other RNA structures shows that the C-C mismatch is the least frequent pair^27–29^, suggesting that it might have an adverse effect in other pathways too.

We found that, at least during plant miRNA biogenesis, the C-C pair is detrimental in different locations within a miRNA precursor. A MD analysis showed that bases at this pair are in continuous movement (shear), while Terbium (III) probing revealed that a dsRNA containing a C-C mismatch is more flexible than one harboring a C-U mismatch. Taken together, these results show that mismatches formed by different nucleotides can affect the structural properties of the foldbacks, which in turn is reflected in a differential miRNA processing efficiency. Other mismatches such as G-G, A-G/G-A are also found at low frequency in plant miRNA precursors and, at least in certain contexts, also impaired miRNA biogenesis. Mutations in the first cleavage site of the *MIR172A* precursor that impaired miRNA biogenesis resulted in an increased accumulation of the pri-miR172 levels, indicating that the first cleavage reaction was compromised, however we cannot rule out the C-C mismatches interfere with other steps of the miRNA pathway as well, such as AGO loading.

Although C-C mismatches are usually excluded from the precursors’ foldbacks, we found a few cases where C-C mismatches are evolutionarily conserved in the precursor structure, such as the one found in the miRNA/miRNA* duplexes of *MIR168*. The later precursor has been shown to produce isomiRs with different features, which in turn is relevant for the selection of the cognate AGO protein^20,30^. A flexible miR168/miR168* duplex structure has been shown to be responsible for the miR168 isomiRs^20^. In this line, the flexibility introduced by a C-C mismatch to dsRNA segments might fulfill specific roles in certain specific contexts. Of all the *MIR172A* variants tested, the only one that generated isomiRs was a precursor harboring a C-C mismatch in the miRNA/miRNA* duplex, thus supporting this possibility.

We found that positions 1, 3, and 23 of the precursors are mostly paired, and showed that closing the secondary structure of *MIR172A* that has an unusual mismatch at position 1 increased miR172 accumulation. In a similar way, introducing a mismatch at position 23 of *MIR164C* systematically reduced its biogenesis. In good agreement, recent results have shown that the processing of the precursors is also favored by a G-C-rich signature in the miRNA/miRNA* duplex^31^, highlighting the importance of a stable secondary structure at certain regions of the plant miRNA precursors.

Structure–function analyses of RNA foldbacks usually focus on the secondary structure, disregarding the importance of the nucleotide identity at unpaired positions. Artificial miRNA (amiRNA) technology is used to downregulate gene expression, and is based on the replacement of the small RNA sequence of a natural miRNA precursor maintaining its secondary structure^32–34^. amiRNAs are especially suited to downregulate gene expression in specific tissues by driving their expression from different promoters. Our findings show the importance of considering the identity of the unpaired nucleotides during the design of these experiments. In the case of artificial miRNAs, we suggest designing precursors harboring only A-C, U-C, or U-U mismatches. Our results could be useful for the design of targeted modifications of *MIRNA* genes in order to modify the expression levels without altering the small RNA sequence. Furthermore, it might be interesting to test whether the identity of the mismatch pairs, especially C-C mismatches, also affects other biological processes.

## Methods

### Sequence analysis of *MIRNAs*

Analysis of nucleotide pairs in the precursors’ sequences was performed for *MIRNAs* conserved in eudicots, using publicly available sequence data^12^. Sixty-one sequences of conserved precursors from *Arabidopsis thaliana*, and 1681 sequences of precursors from eudicot species were analyzed (Supplementary Fig. 1 and Source Data). Secondary structures of miRNA precursors were predicted with the Mfold web server (http://unafold.rna.albany.edu/?q=mfold/RNA-Folding-Form) using default parameters^35^. For human miRNA analysis, information of 477 precursors available in miRBase v22 were included^36^. Data used for these analyses is available in Source Data file.

### Plant material

*A. thaliana* ecotype Columbia was used for all experiments. Plants were grown at 22 °C under continuous light at 100 µmol photons m^−2^ s^−1^. Transgenic plants were selected on Murashige and Skoog media with 50 µg/ml kanamycin. For the experiments included in Figs. 2c, 3d–e, and Supplementary Figs 4a, 8d, e, transgenic plants were transferred to soil after seven days and continued growing in long days. Upon flowering, primary inflorescences were collected and phenotypic analysis was performed (see Source Data file for additional details). Seedlings were collected after 10 days for RNA extraction (experiments included in Figs. 3–5 and related Supplementary Figures).

### Transgenic lines

miRNA precursors corresponding to *MIR165A* (AT1G01183), *MIR164C* (AT5G27807), *MIR172A* (AT2G28056), and *MIR397A* (AT4G05105) were expressed under the control of the CaMV 35 S promoter of the binary vector CHF3^37^. Sequences of all the constructs used and prepared in this article are indicated in Supplementary Table 3.

### RNA analysis

Ten-day old seedlings or inflorescences were ground in liquid nitrogen, and RNA extraction was performed with TRI Reagent® (MRC) following the product instructions. Each sample analyzed contained material from 10 independent primary transgenic plants. Small RNA blots were performed as described previously^38^. Probes used for detection of each miRNA are listed in Supplementary Table 3. Note that we used 19 or 20 nt long probes that perfectly match to all the mutant miRNAs generated in our experiments. In brief, 3–15 µg of total RNA were resolved on 17% polyacrylamide denaturing gels (7 M Urea), transferred to a Nytran SPC membrane (GE Healthcare) and UV-crosslinked. Probes were 5′-end-labeled with [γ-^32^P] ATP using T4 polynucleotide kinase (Thermo Scientific). Detection of miR172a* was carried out first, the membraned stripped and checked, and then used for miR172. Images were obtained with a Typhoon FLA 7000 (GE Healthcare) and the signal intensity of each band was determined using the GelQuant.NET software (biochemlabsolutions.com). The intensity values of the bands detected with the miRNA probes were normalized to the signal obtained with the U6 snRNA probe. These values were then divided by the normalized signal intensity of the sample corresponding to the transgenics with the empty vector or with the wt precursor. All the quantifications are detailed in Source Data file.

Primary transcripts and small RNAs were measured by RT-qPCR in at least three independent samples (two technical replicates) as described before^12^. In brief, 500 ng of total RNA were treated with RQ1 RNAse-free DNase (Promega). First strand synthesis was carried out with M-MLV reverse transcriptase (Invitrogen) using dTV oligonucleotides for polyadenylated RNAs and stem-loop oligonucleotides for mature miRNAs or miR172a*^39,40^. PCR reactions were performed in an AriaMx Real-time PCR System (Agilent) using SYBR Green I (Roche) to monitor double-stranded DNA synthesis. Pri-miRNA levels were normalized to *RPS26* (AT3G56340) expression. Oligonucleotides used are listed in Supplementary Table 2.

The rapid amplification of 5′ cDNA ends (5′ RACE) method to detect cuts in the precursors was carried out as described previously^14^. In brief, total RNA was treated with DNAse, purified and the 5′-end of processing intermediates were ligated to a RNA adapter (GeneRacer™ RNA, Thermofisher). Then, polyadenylated RNA was purified with PolyATtract® mRNA Isolation Systems (Promega). First strand synthesis was carried out with an oligonucleotide specific for transcripts derived from CHF3 vector. Hemi-nested PCRs were performed with two vector-specific reverse oligonucleotides. PCR products were purified, cloned, and sequenced. Oligonucleotides used are listed in Supplementary Table 2.

### RNA structure

Pri-miR172a wt (C-U), 1C-G, and 1C-C versions were transcribed in vitro using T7 RNA polymerase as described before^6^. Alkaline hydrolysis of transcripts was used as a ruler, as well as MW standards. Terbium (Tb III) induced autohydrolysis reactions were carried out by incubating 1.5 µl RNA (1 µM), 1 µl folding buffer (20 mM Tris pH 7.5, 25 mM KCl and 10 mM MgCl2), 0.3 µl of *Escherichia coli* tRNA (0.1 mg/ml) and 6.2 µl water for 15 min at room temperature, after which 1 µl of 100 mM Tb (III) was added. The reaction was carried out for 15 min, and then stopped by precipitating the RNA overnight. For control, 1 µl of water was added instead of Tb (III). After RNA precipitation and resuspension, the digested products were analyzed in 8% or 12% (w/v) denaturing polyacrylamide gels, respectively. The results were visualized by Phosphor Imaging (Typhoon FLA 7000, GE). Image processing, band profile acquisition and quantification were performed with the FIJI software^41^.

### Statistical methods

Tests applied are indicated in the Figure captions. Information about size of samples (*n*), and the respective tables of each statistical test are included in Source Data file. RT-qPCR and plant phenotype data were analyzed with InfoStat software^42^.

### MD simulations

The initial structural models for the dsRNAs were built from a portion of the sequences of *MIR172A* from position −10 to position 10 and *MIR164C* from position 13 to position 32 using NAB^43^ (Fig. 5a and Supplementary Fig. 10a). Each model was immersed in a truncated octahedral periodic box with a minimum solute-wall distance of 8 Å filled with around 10,000 water molecules and 0.1 M NaCl. Each system was then subjected to 50 ns of MD simulation (detailed below). Subsequently, the nucleotide pair at position 1 of *MIR172A* and position 23 of *MIR164C* were mutated in silico to generate the 16 different possible combinations of the 4 nucleotides. All models were further subjected to 100 ns of MD simulations, during which their structures were analyzed. MD simulations were performed with the Amber 14 package^44^. The force field used was ff99bsc0 with χOL3 modifications^45,40^ for dsRNAs and TIP3P for water^46^. Particle-mesh Ewald^47^ was implemented for long range interactions and a cutoff distance of 12 Å was used. Temperature and pressure were regulated with Berendsen thermostat and barostat^48^ with a coupling constant of 2 ps. In addition, we performed two extra versions of each MD simulation of *MIR172A* using Langevin thermostat with a collision frequency of 5 ps^−1^ to improve statistical significance and ensure results are not dependent on thermostat choice. A comparison of the calculated shear parameter (see below) is shown in Fig. 5d and Supplementary Data 1, in which the reproducibility of the results can be seen. The simulation protocols, validated in previous work^49^, started with an energy minimization of the initial structure, followed by a heating phase from 0 K to 300 K through 20 ps at constant volume and an equilibration phase at 300 K and 1 bar of constant pressure through 20 ps to reach proper density. These equilibrated structures were the starting point for 100 ns of MD simulations at 300 K in the NVT ensemble. To avoid artifacts of unpairing at the base pairs of the ends of the models, a simple distance restraint was applied to base pairs located at the ends of the strands so that their hydrogen bonds are maintained. Structural parameters for the dsRNAs were calculated using Curves+^50^, which include axial bend, shear, buckle, stretch, propeller, stagger, opening, shift, tilt, slide, rill, rise, and twist (Supplementary Data 1 and 2). Base pair conformational clustering was performed using the hierarchical agglomerative approach from the cpptraj module of Amber14 (Supplementary Data 3).

### Reporting summary

Further information on research design is available in the Nature Research Reporting Summary linked to this article.

## Supplementary information

Supplementary Information

Supplementary Data 1

Supplementary Data 2

Supplementary Data 3

Reporting Summary

## Data Availability

All relevant data are included in the paper and/or its Supplementay Information files. Data corresponding to Figures 1–5, Supplementary Figure 2 and Supplementary Figures 4-9 are detailed in Source Data file provided with this manuscript.

## Source data

Source Data
