# Supplementary material for: Identification of key sequence features required for microRNA biogenesis in plants
Source: Nat Commun. 2020 Oct 21;11:5320. doi: 10.1038/s41467-020-19129-6 (PMC7577975; doi:10.1038/s41467-020-19129-6)
Supplement: Supplementary file 1 — Supplementary Information [file 41467_2020_19129_MOESM1_ESM.pdf]

Supplementary Information:

Identification of key sequence features required for microRNA biogenesis in plants

Rojas *et al.*

Supplementary Figures.

Supplementary Figure 1: List of the plant miRNA precursors analyzed.

Supplementary Figure 2: Pairs of nucleotides at DROSHA and Dicer cleavage sites of human miRNA precursors.

Supplementary Figure 3: Arabidopsis miRNA precursors with unpaired nucleotides at position 1.

Supplementary Figure 4: Characterization of plants overexpressing *MIR172A* wt (1 C-U) and mutated (1 C-G).

Supplementary Figure 5: Characterization of paired variants at position 1 of *MIR172A*.

Supplementary Figure 6: miRNA accumulation of variants with a mismatch at position 1 of *MIR172A.*

Supplementary Figure 7: miRNA accumulation in seedlings expressing different precursor mutants.

Supplementary Figure 8: miRNA activity in plants expressing different *MIRNA* variants.

Supplementary Figure 9: Conservation of the C-C mismatch in *MIR168* of different plant species.

Supplementary Figure 10: Molecular Dynamics simulation of *MIR164C* variants.

Supplementary Figure 11: Uncropped images shown in Figure 2-4.

Supplementary Figure 12: Uncropped images shown in Figures 2j and 5e.

Supplementary Fig. 13: Uncropped images shown in Supplementary Figures 5-7.

Supplementary Tables.

Supplementary Table 1: Nucleotide frequencies at precursors cleavage sites.

Supplementary Table 2: Oligonucleotides used in this study.

Supplementary Table 3: Vector sequences generated and used in this study.

Supplementary Figure 1:

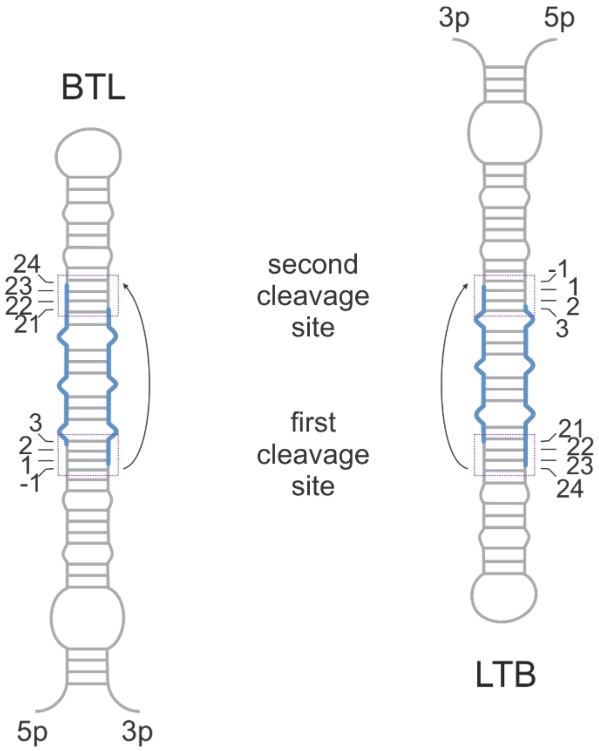

BTL-Eudicots	BTL-Arabidopsis	LTB-Eudicots	LTB-Arabidopsis
miR164a-c	miR164a-c	miR156a-h	miR156a-d,h
miR165a-b	miR165a-b	miR157a-d	miR157a-c
miR166a-g	miR166a-g	miR159a-c	miR160a-c
miR167a-d	miR167a,c-d	miR160a-c	miR162a
miR168a-b	miR168a-b	miR162a-b	miR171b-c
miR169a-n	miR169a	miR171b-c	miR408
miR170	miR170	miR319a-c	
miR171a	miR171a	miR408	
miR172a-e	miR172a-e		
miR390a-b	miR390a		
miR393a-b	miR393a-b		
miR394a-b	miR395a-c,f		
miR395a-f	miR396a-b		
miR396a-b	miR397a-b		
miR397a-b	miR398a-c		
miR398a-c	miR399a-f		
miR399a-f	miR403		
miR403	miR827		
miR827			

Supplementary Figure 1: List of the plant miRNA precursors analyzed.

List of base-to-loop (BTL) and loop-to-base (LTB) precursors used for the analysis of the nucleotide composition. The sequences are shown in the Source Data File.

Supplementary Figure 2:

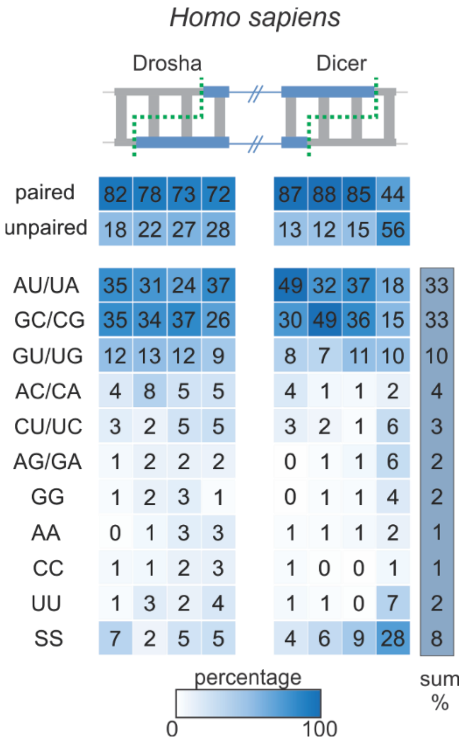

Supplementary Figure 2: Pairs of nucleotides at DROSHA and Dicer cleavage sites of human miRNA precursors.

The miRNA precursor sequences were obtained from miRBase v22 (See Source Data File).

Supplementary Figure 3:

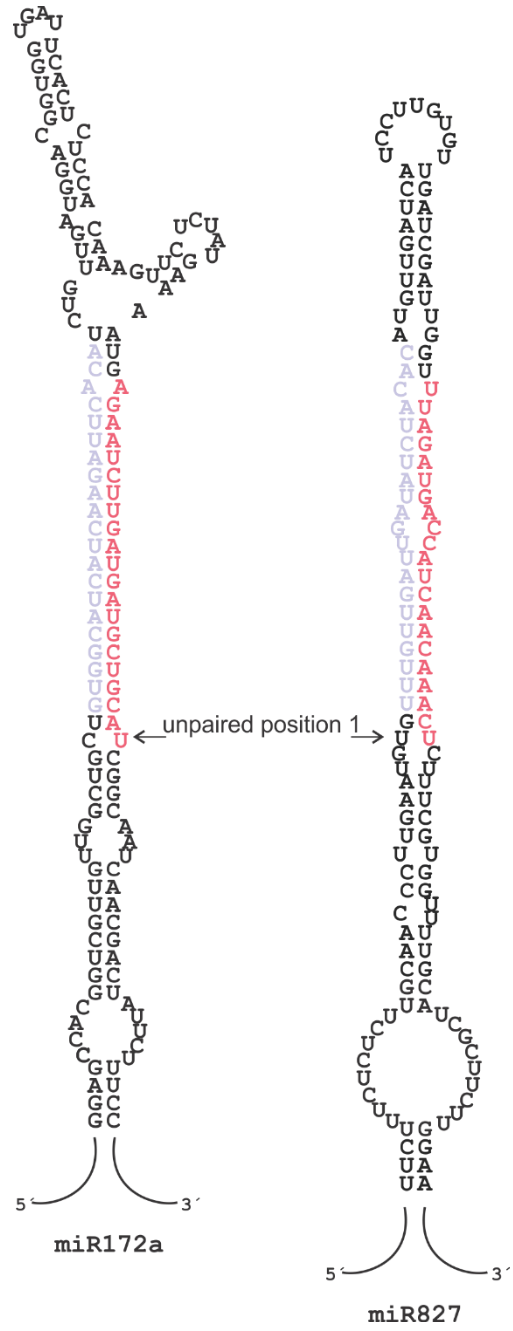

Supplementary Figure 3: Arabidopsis miRNA precursors with unpaired nucleotides at position 1.

Fold-back structure of miR172a and miR827 precursors of *Arabidopsis thaliana*. miRNA is indicated in pink and the miRNA* in purple.

Supplementary Figure 4:

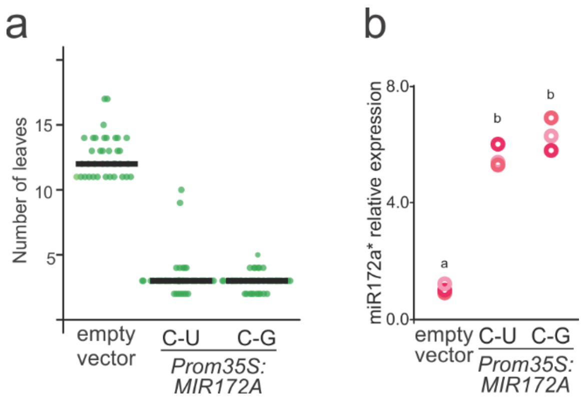

Supplementary Figure 4: Characterization of plants overexpressing *MIR172A* wt (1 C-U) and mutated (1 C-G).

a) Number of rosette leaves at flowering.
b) miR172a* levels as determined by RT-qPCR in inflorescences of empty vector, *MIR172A* wt (1 C-U) or mutated 1 C-G. Different letters indicate statistically significant differences (ANOVA followed by Tukey's multiple comparison test ($p<0.05$)).

Supplementary Figure 5:

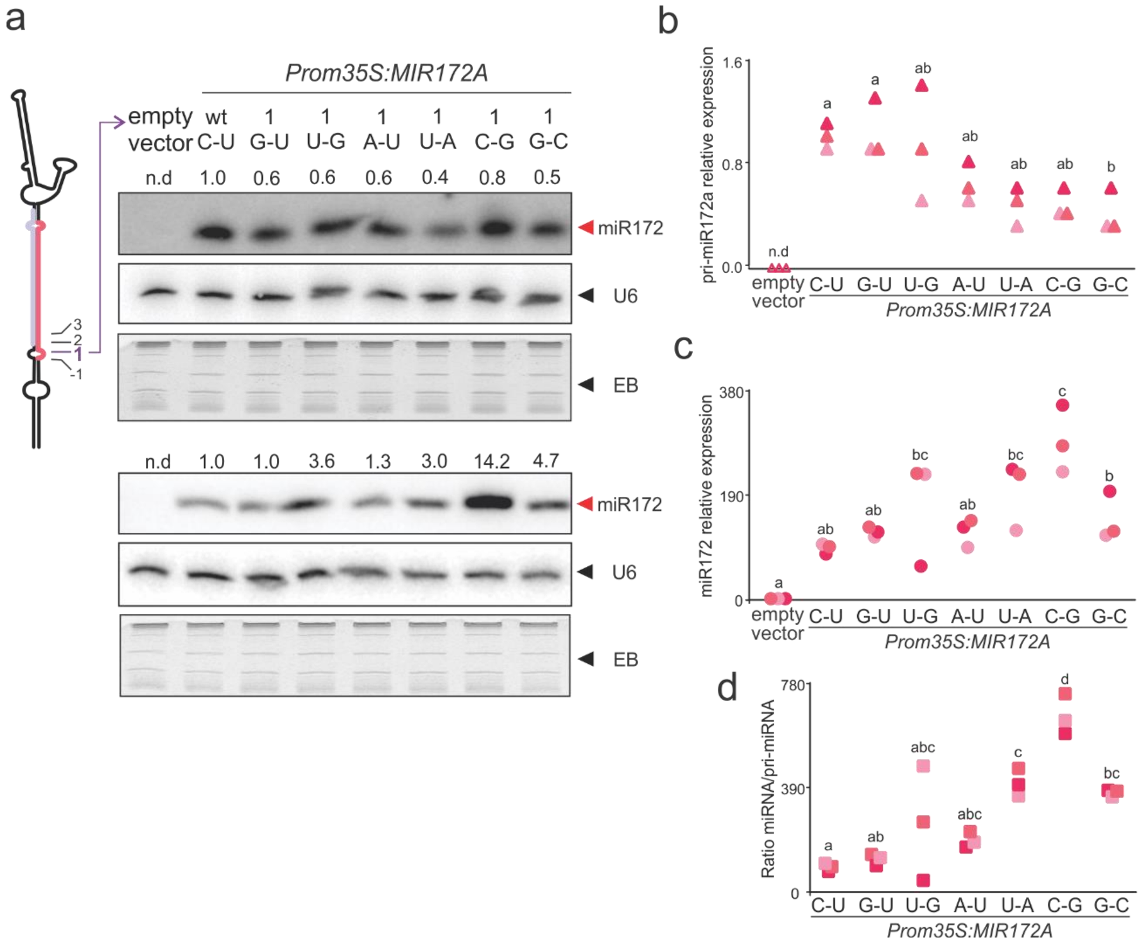

Supplementary Figure 5: Characterization of paired variants at position 1 of *MIR172A*.

a) Biological replicates of small RNA blot shown in Figure 3a. See Supplementary Figure 13a for uncropped images of the gels.
b-c) Accumulation of pri-miRNA (b) and miRNA (c) as determined by RT-qPCR in transgenic seedlings with the empty vector, wt *MIR172A* (1C-U), or paired *MIR172A* variants.
d) Ratios of miRNA (c) and pri-miRNA (b).
Each sample is a pool of at least ten independent transgenic plants. Different letters indicate statistically significant differences (ANOVA followed by Tukey's multiple comparison test ($p<0.05$)). The same data corresponding to empty vector, *MIR172A* wt (1 C-U) and 1 C-G variant are also shown in Figure 2d-f.

Supplementary Figure 6:

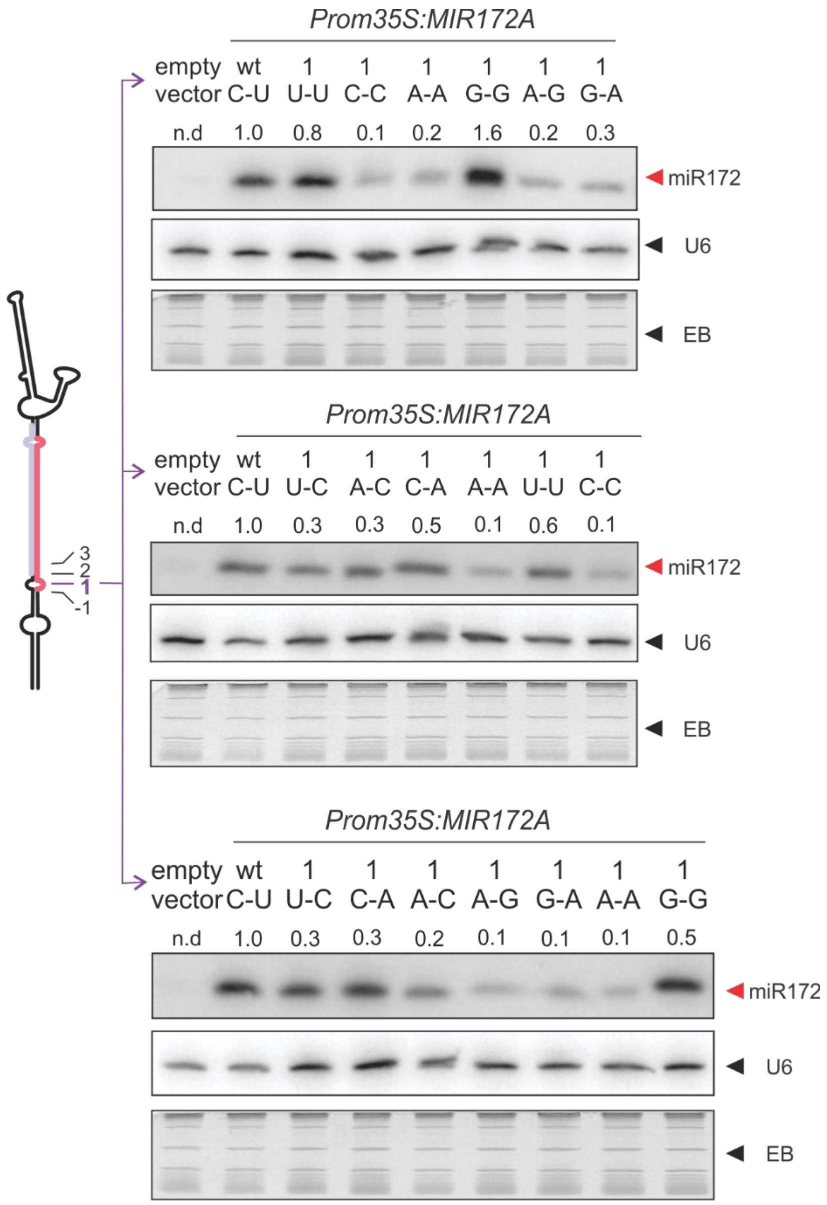

Supplementary Figure 6: miRNA accumulation of variants with a mismatch at position 1 of *MIR172A*. Small RNA blots are biological replicates of blots in Figure 3b. See Supplementary Figure 13b for uncropped images.

Supplementary Figure 7:

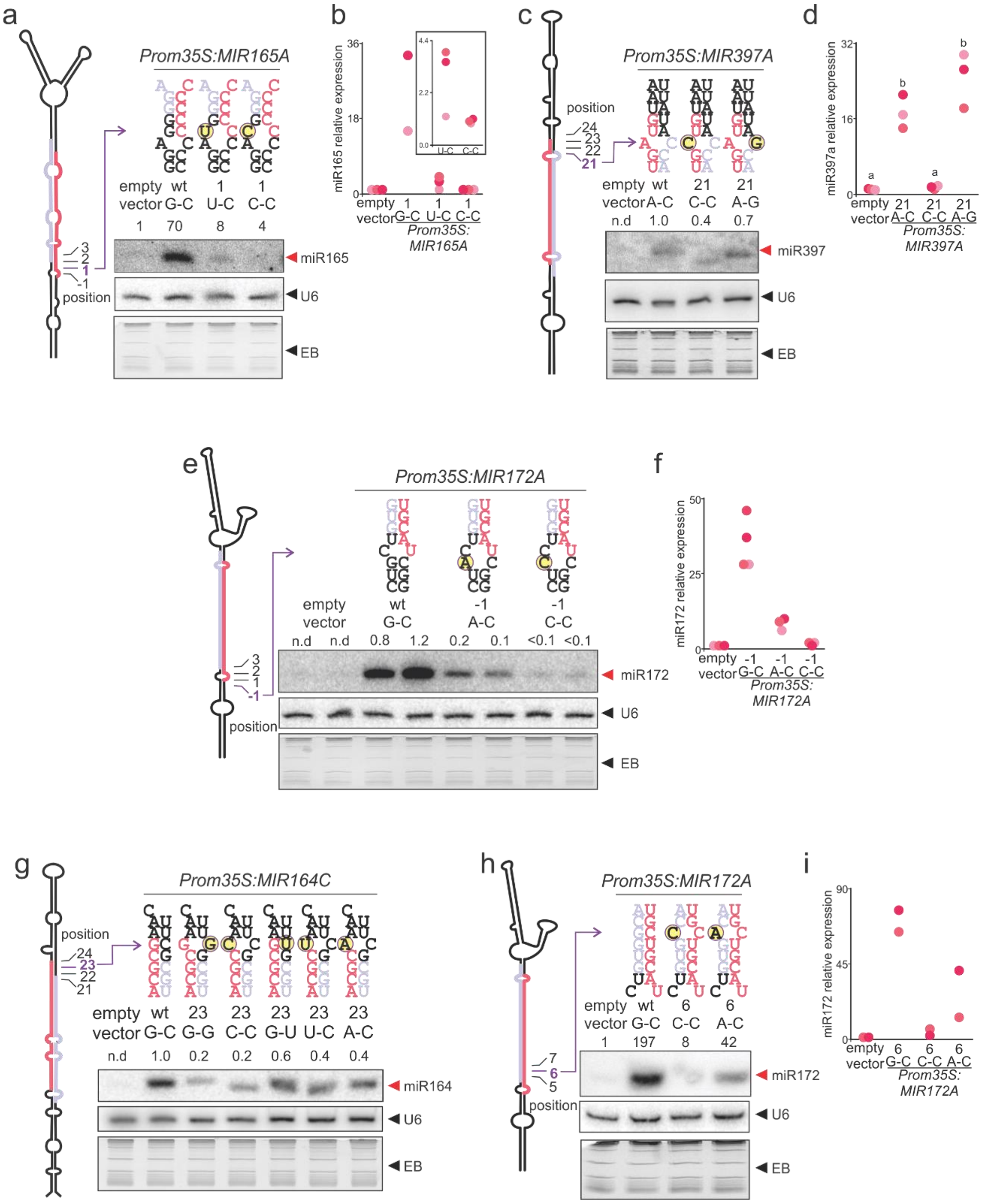

Supplementary Figure 7: miRNA accumulation in seedlings expressing different precursor mutants.

Biological replicates of small RNA blots shown in Figure 4 (see Supplementary Fig. 13c-g for uncropped images), and miRNA accumulation measured by RT-qPCR. Different letters indicate statistically significant differences (ANOVA followed by Tukey’s multiple comparison test ($p<0.05$)).

a-b) *MIR165A* wt (1G-C), 1U-C and 1C-C variants. An inset is included in (b) for better comparison between U-C and C-C variants.

c-d) *MIR397A* wt (21A-C), 21C-C and 21A-G variants.

e-f) *MIR172A* wt (-1G-C), -1A-C and -1C-C variants.
g) *MIR164C* wt (23G-C), 23A-C, 23U-C, 23G-U, 23C-C and 23G-G variants.
h-i) *MIR172A* wt (6G-C), 6C-C and 6A-C variants.

Supplementary Figure 8:

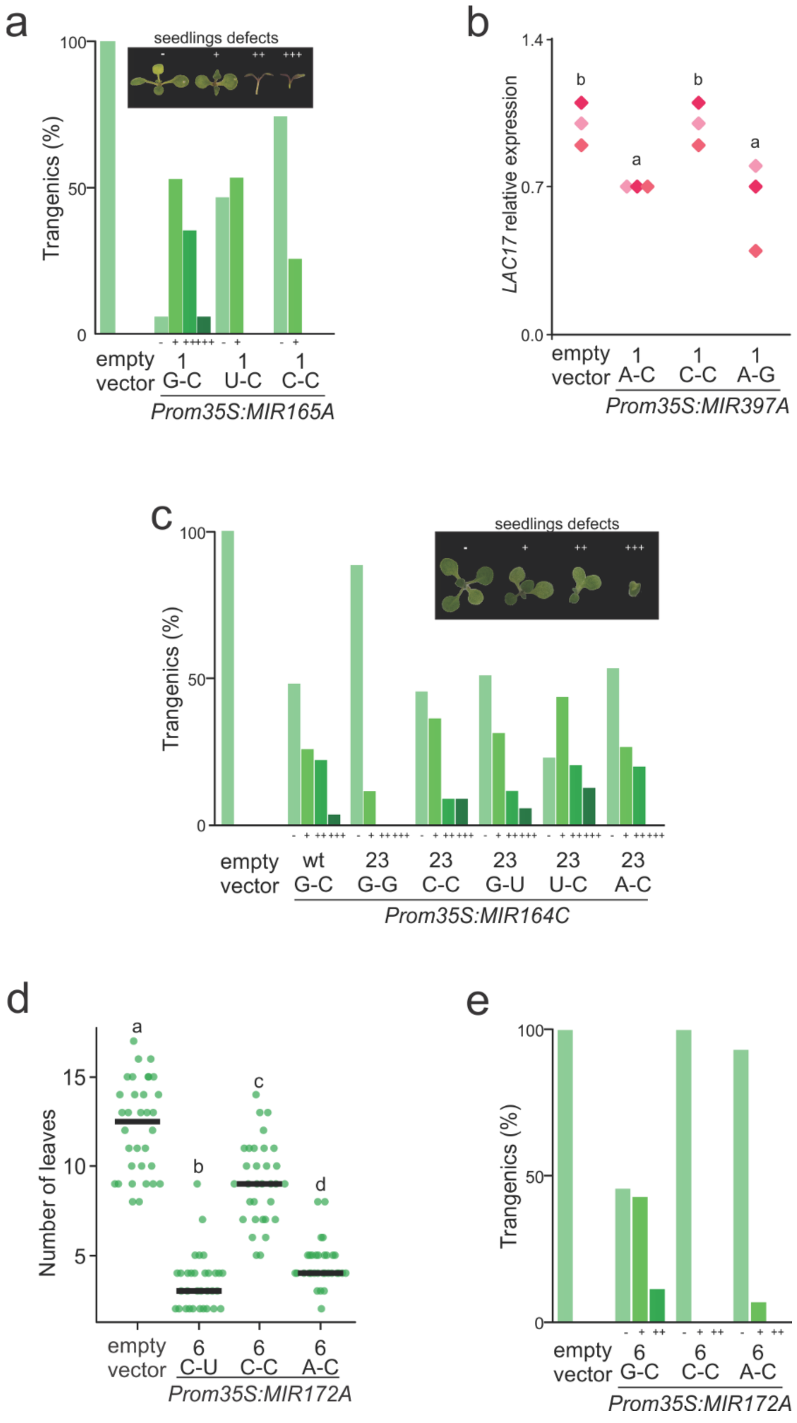

Supplementary Figure 8: miRNA activity in plants expressing different *MIRNA* variants. The sequence of the mutant *MIRNAs* are shown in Fig. 4.

a) Percentage of seedling with no (-), curled (+), radial (++) or pinhead (+++) phenotype of T1 population expressing *MIR165A* wt (1G-C), 1U-C or 1C-C variant. A representative seedling for each phenotype is indicated in the inset as previously reported [Zhu et al. (2013) Nat Struct Mol Biol 20, 1106-1115; Chorostecki et al. (2017) Plant Cell 29, 1248-1261].

b) RT-qPCR for the miR397a target *LAC17*. Three biological replicates of pooled T1 seedlings expressing *MIR397A* wt (21A-C), 21C-C or 21A-G variant were analyzed. Different letters

indicate statistically significant differences (ANOVA followed by Tukey's multiple comparison test ($p<0.05$)).

c) Percentage of seedlings with aligned (-), misaligned (+), partially fused (++) or cup-shaped (+++) cotyledons of T1 population expressing *MIR164C* wt (23G-C), 23A-C, 23U-C, 23G-U, 23C-C or 23G-G variant. A representative seedling for each phenotype is indicated in the inset as previously reported [Laufs et al. (2004) Development 131, 4311-4322; Schwab et al. (2005) Dev Cell 8, 517-527].

d-e) Phenotypes of plants overexpressing *MIR172A* wt (6G-C), 6 C-C or 6A-C variants. Number of leaves at flowering (d), letters indicates statistically significant differences (Kruskal-Wallis multiple comparison test, $p<0.05$). Percentage of plants with no (-), moderate (+), and strong (++) flower defects (e). A representative flower for each phenotype is indicated in Figure 2c as previously reported [Aukerman and Sakai (2003) Plant Cell 15, 2730-2741; Chen (2004) Science 303, 2022-2025; Mateos et al. (2010) Curr Biol 20, 49-54].

Supplementary Figure 9:

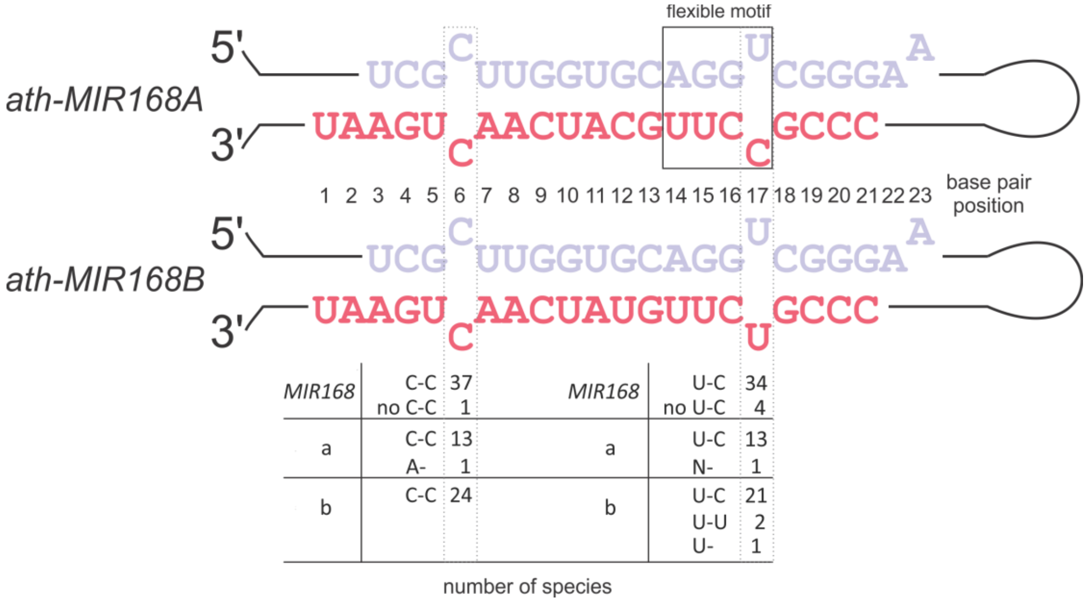

Supplementary Figure 9: Conservation of the C-C mismatch in *MIR168* of different plant species.
Schematic representations of *MIR168A* and *MIR168B* precursors from *Arabidopsis thaliana*. The miRNA is in pink and the miRNA* in purple. The table indicates the number of *MIR168* sequences of eudicot species with a C-C mismatch at position 6 and other base pair combinations found to a lesser extent. The Table also shows the conservation of the U-C mismatch in the flexible motif already reported [Iki T, et al. Structural Flexibility Enables Alternative Maturation, ARGONAUTE Sorting and Activities of miR168, a Global Gene Silencing Regulator in Plants. Mol Plant 11, 1008-1023 (2018)]. See Source Data File for more details.

Supplementary Figure 10:

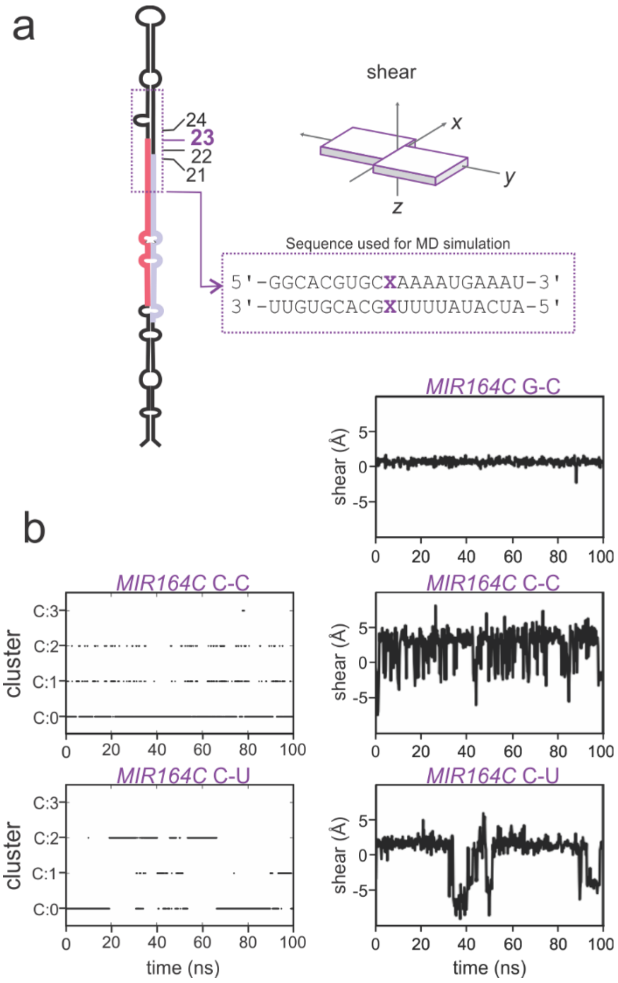

Supplementary Figure 10: Molecular Dynamics simulation of *MIR164C* variants.

a) Schematic representation of *MIR164C* precursor. A dashed outline indicates the region used for Molecular Dynamics (MD) simulations. The "X" in the nucleotide sequence indicates the modified position for the simulation of each variant. The "shear" parameter (an in-plane shift of the bases within the base pair) is depicted on the right (movement of bases along the *x* axis).

b) Results of MD for *MIR164C* G-C (wt), C-C and C-U variants in position 23. The clustering (G-C always remains in the same conformation) and the shear parameter are indicated. See Supplementary Data 2 for results of the other nucleotide variants.

Supplementary Figure 11:

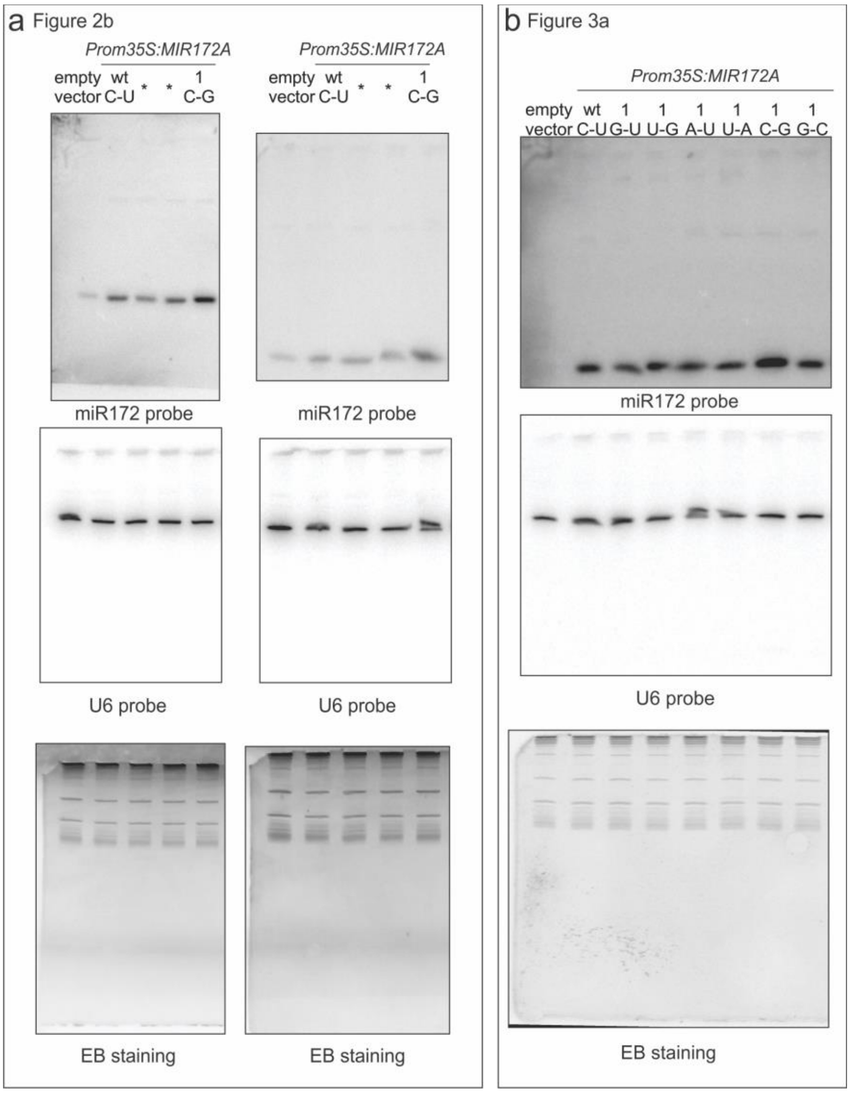

c Figure 3b, left -

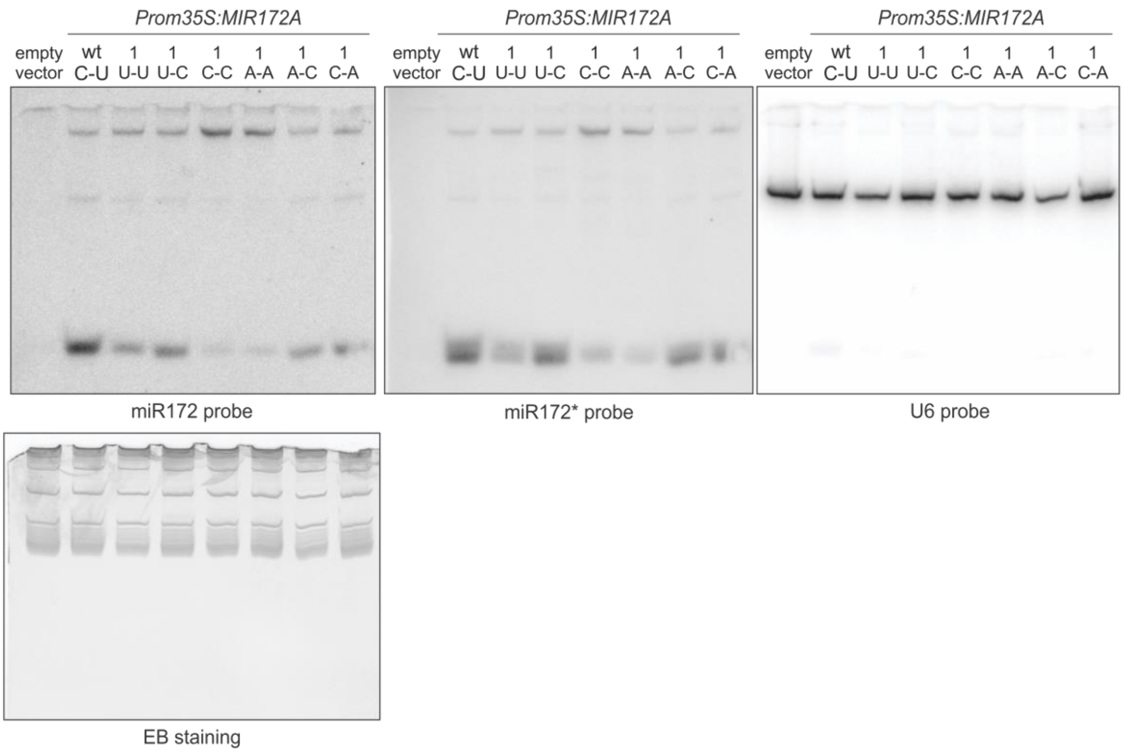

d Figure 3b, right

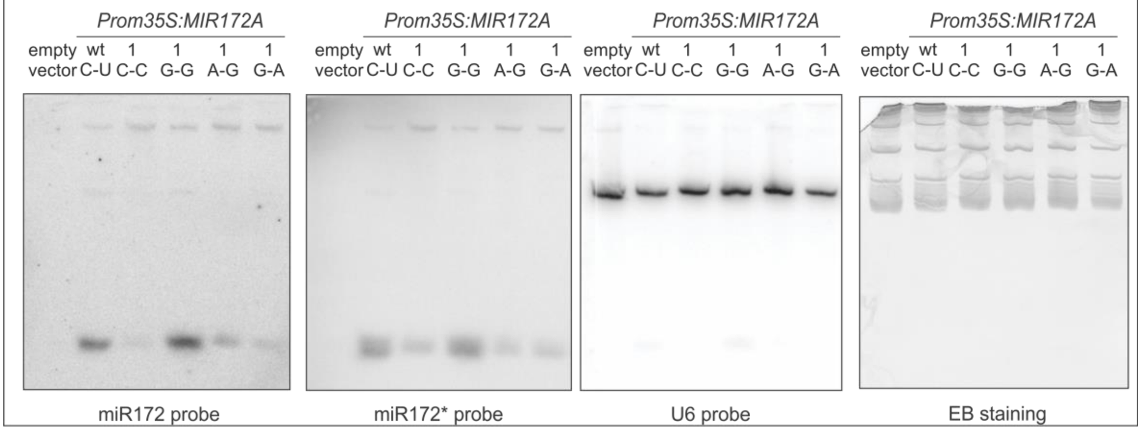

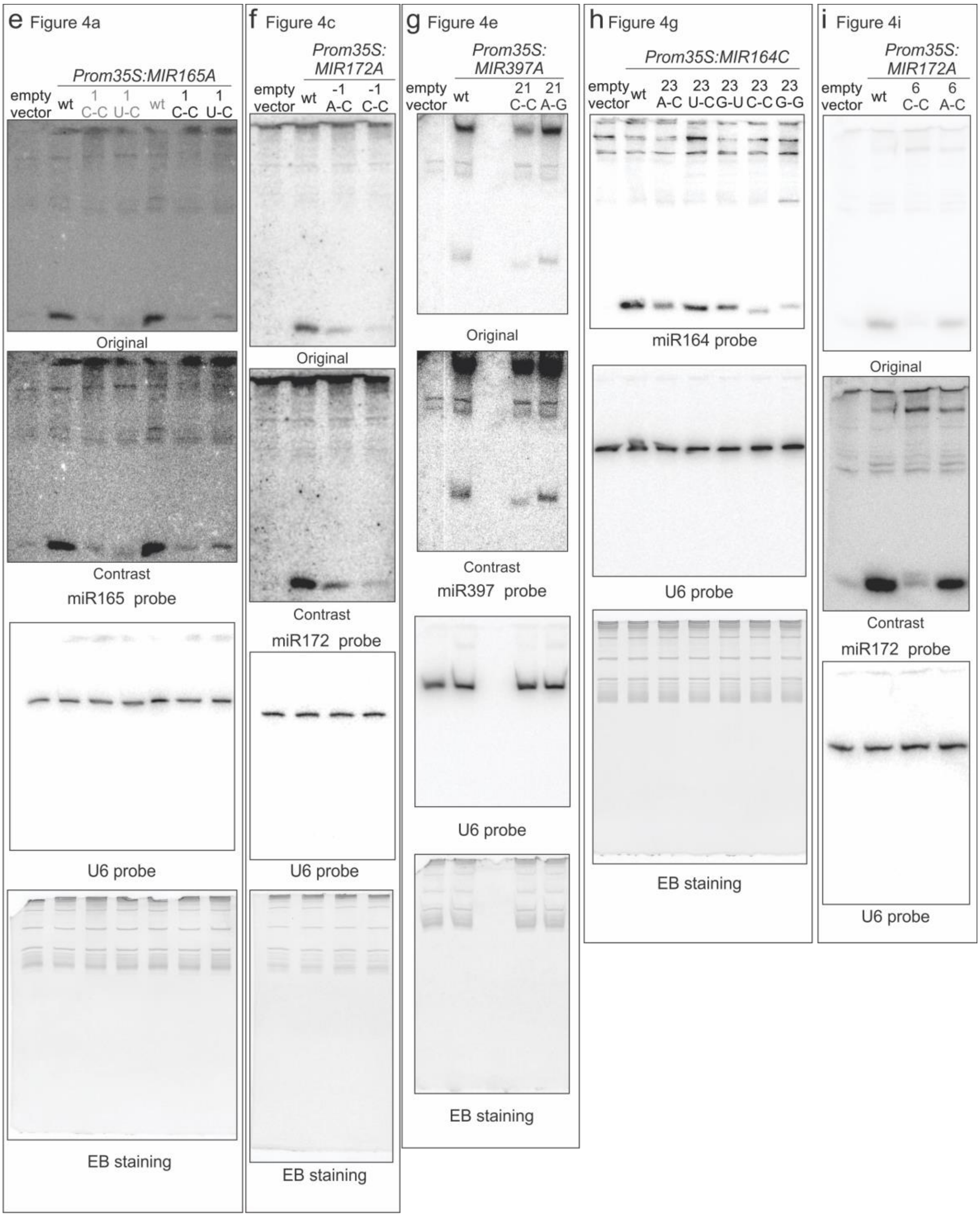

Supplementary Figure 11: Uncropped images shown in Figure 2-4. Below each image, the probe and/or ethidium bromide staining (EB) are indicated. Each panel is labeled with the corresponding manuscript Figure where the images are shown.

a) Lanes cropped in Figure 2b are indicated with (*) character.

e) Lanes labeled in grey were cropped in Figure 4a.

g) Empty lane was cropped in Figure 4e.

Supplementary Figure 12:

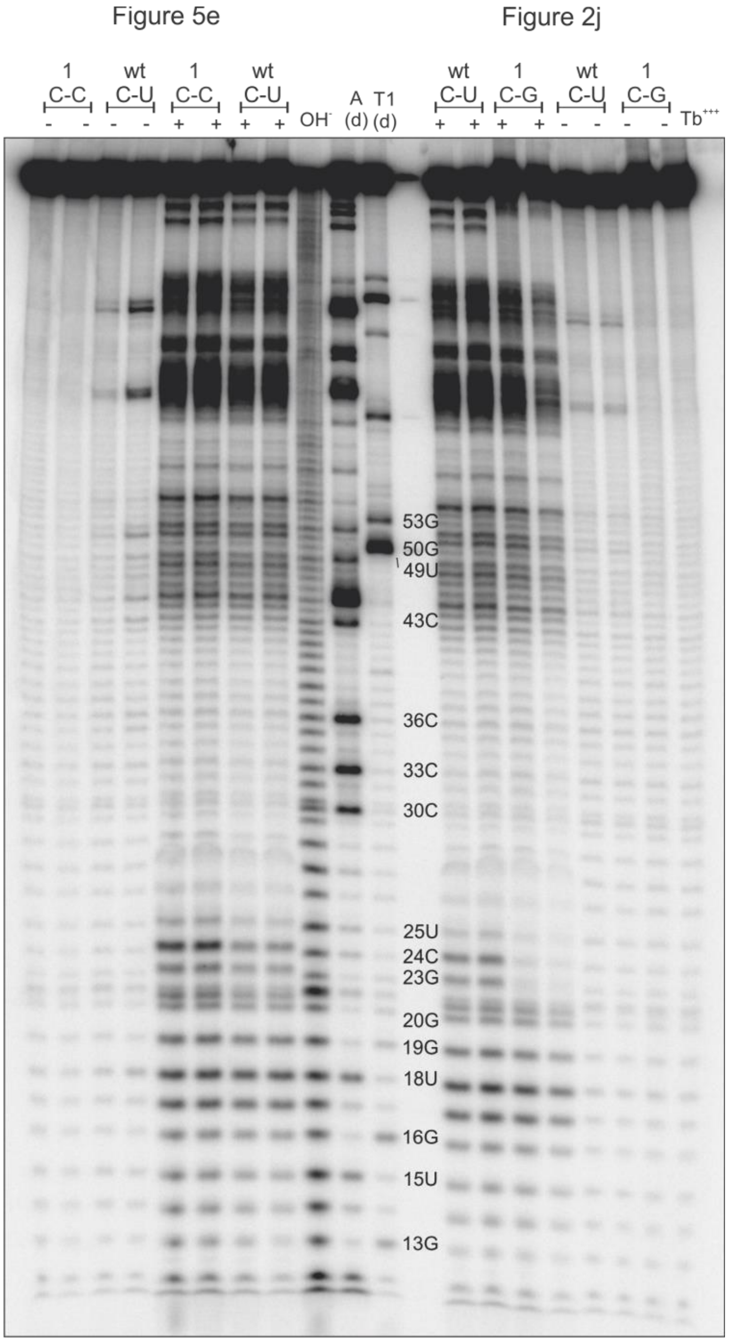

Supplementary Fig 12: Uncropped images shown in Figures 2j and 5e. Lanes corresponding to transcripts incubated in control conditions (without Terbium) are not included in Figure 2 and 5. Numbers and letters indicate the nucleotide position at the pri-miR172a transcript.

Supplementary Fig. 13:

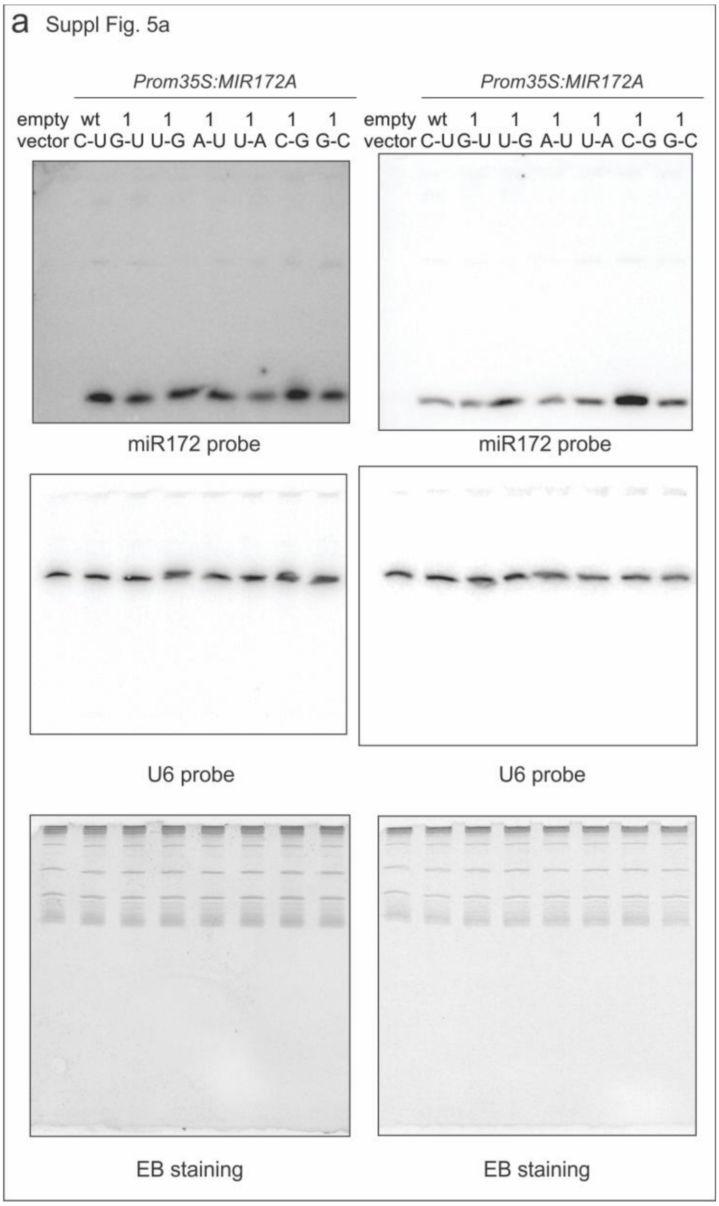

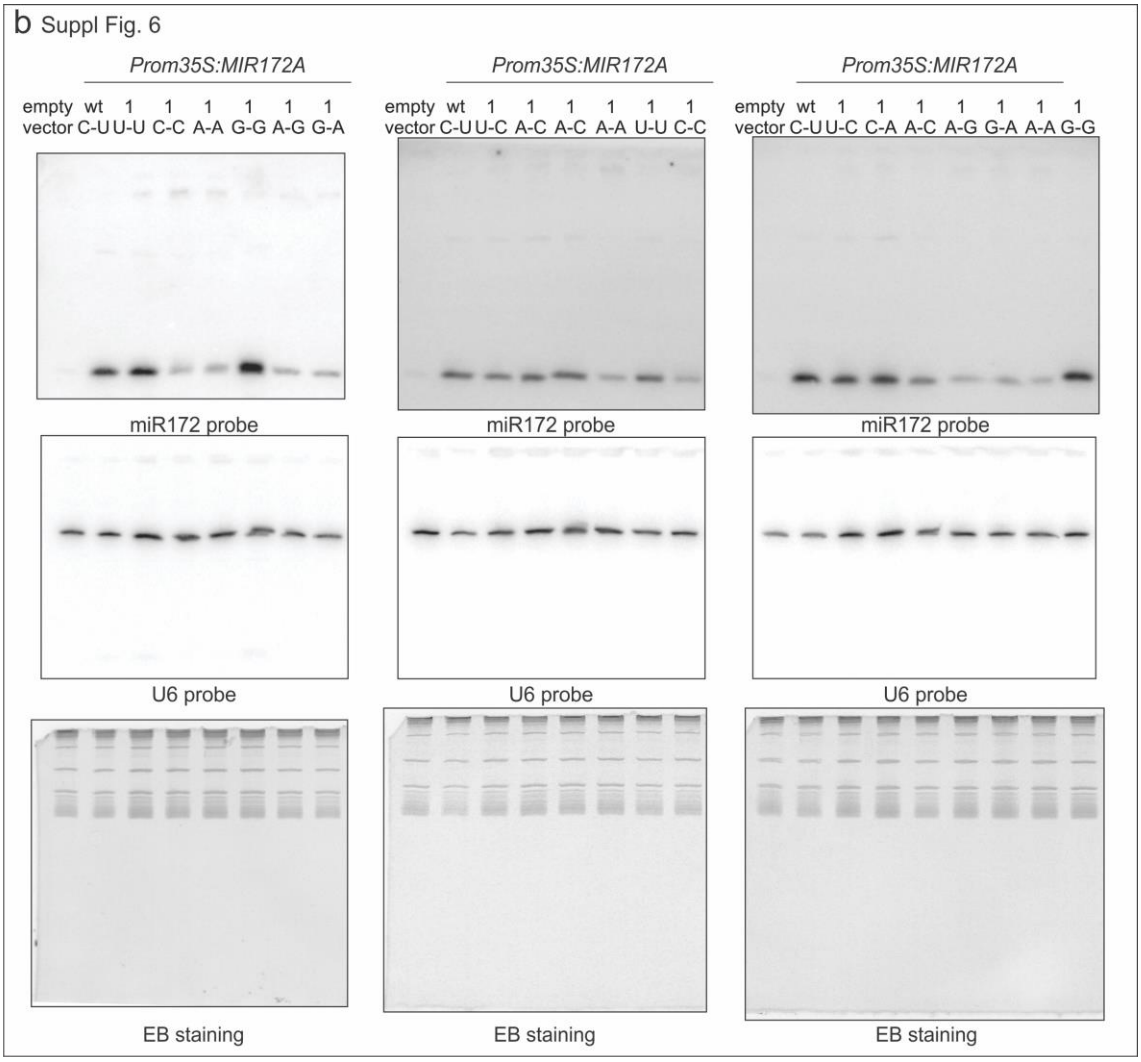
b Suppl Fig. 6
Prom35S:MIR172A
empty vector
wt C-U
1 U-U
1 C-C
1 A-A
1 G-G
1 A-G
1 G-A
1 U-C
1 A-C
1 C-A
1 A-G
1 G-A
miR172 probe
U6 probe
EB staining

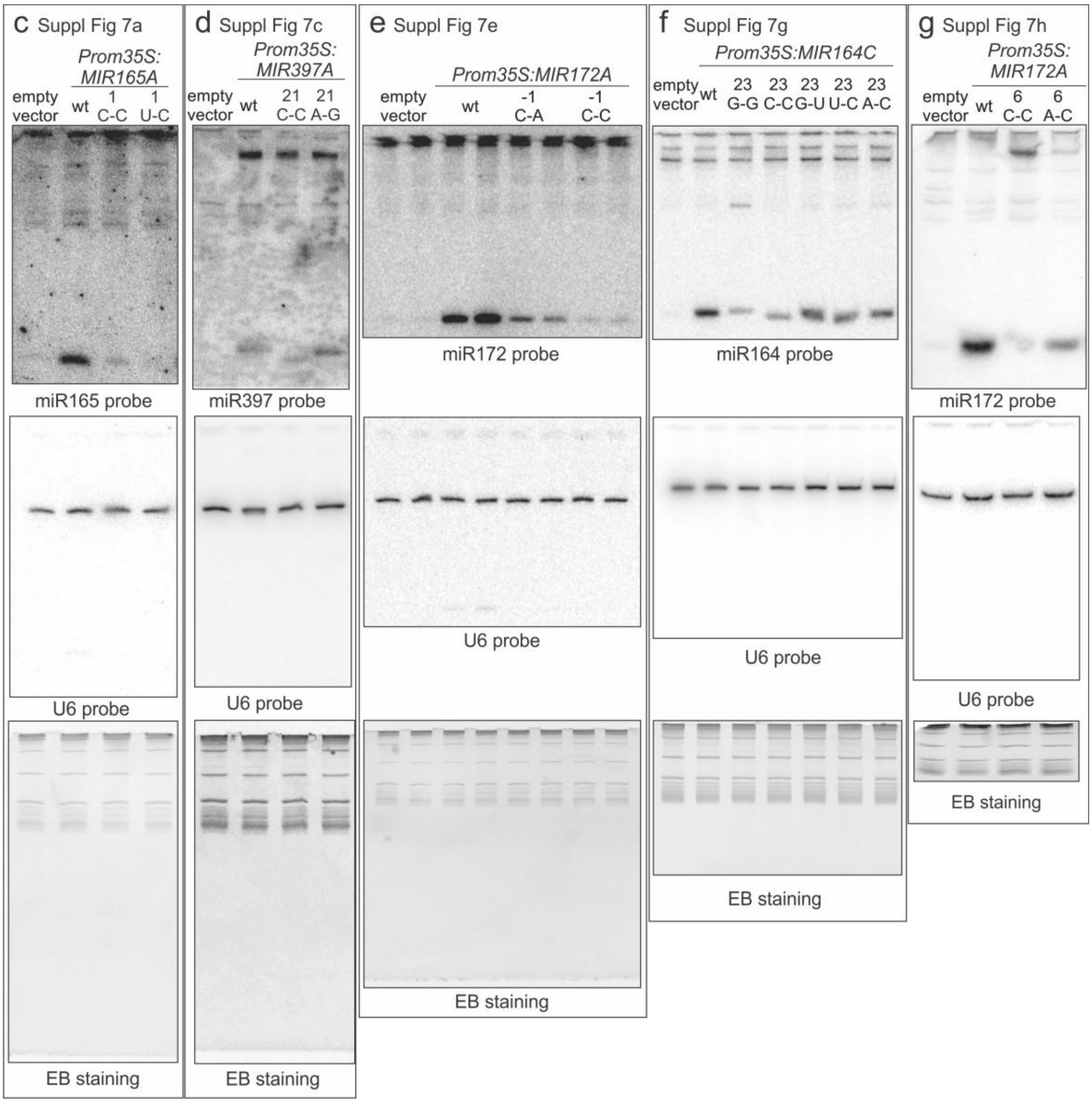

Supplementary Fig. 13: Uncropped images shown in Supplementary Figures 5-7. Below each image, the probe or the ethidium bromide staining (EB) is indicated. Each panel is labeled with the corresponding Supplementary Figure where images are shown.

Supplementary Table 1: Nucleotide frequencies at precursors cleavage sites.

	**Fig 1b, *Arabidopsis thaliana***		**Fig 1c, Eudicots**		**Fig 1d, Cleavage sites *A. thaliana***		**Fig 1d, Cleavage sites Eudicots**		**Supplementary Figure 2, Cleavage sites *Homo sapiens***	
**nucleotide**	sum	%	sum	%	sum	%	sum	%	sum	%
**A**	3102	24.9%	38570	24%	249	26.3%	6084	24.8%	1621	23.0%
**U**	3672	29.5%	47489	30%	250	26.5%	6670	27.2%	1947	27.6%
**C**	2744	22.1%	35634	22%	232	24.6%	5964	24.3%	1637	23.2%
**G**	2924	23.5%	39170	24%	214	22.6%	5784	23.6%	1847	26.2%
**sum**	12442		160863		945		24502		7052	

Supplementary Table 2: Oligonucleotides used in this study.

**Name**	**sequence**	**used for**	**notes**
**dTV**	TTT TTT TTT TTT TTT TTT TTT TTT V	Reverse transcription	
**CHF3_Fw**	AACACGGGGGACGAGCTC	qPCR	complementary to CHF3 vector, used for pri-miR172a and pri-miR397a
**pri-miR172a_Rv**	TGAATCACCACCGTCCATCAAC	qPCR	complementary to pri-miR172a
**pri-miR164c_Fw**	GCGGAGCTCGAGAGAAAGTAAT GGGTGAGTAACAC	qPCR	complementary to pri-miR164c
**pri-miR164c_Rv**	CGCGGATCCCATCCTCGGGGAGG GGGAGAGACACG	qPCR	
**pri-miR165a_Fw**	CCGAGCTCGAGGGGAATGTTGTCT GGATC	qPCR	complementary to pri-miR165a
**pri-miR165a_Rv**	GGGGATCCGAGGCAATAACATG	qPCR	
**pri-miR397a_Rv**	CATAATTGAGCGCAACGCTG	qPCR	complementary to pri-miR397a
**RPS26C_Fw**	GACTTTCAAGCGCAGGAATGGTG	qPCR	gene used for normalization
**RPS26C_Rv**	CCTTGTCCTTGGGGCAACACTTT	qPCR	
**SLO miR172**	GTCTCCTCTGGTGCAGGGTCCGAGGTA TTCGCACCAGAGGAGACNTGCAG	Reverse transcription	For all the miR172a 3' variants
**SLO miR172a***	GTCTCCTCTGGTGCAGGGTCCGAGGTA TTCGCACCAGAGGAGACTGNGAA	Reverse transcription	
**SLO miR165**	GTCTCCTCTGGTGCAGGGTCCGAGGTA TTCGCACCAGAGGAGACGGGGRA	Reverse transcription	
**SLO miR397a**	GTCTCCTCTGGTGCAGGGTCCGAGGTA TTCGCACCAGAGGAGACCAKCAA	Reverse transcription	

**miRNA_Rv**	TGGTGCAGGGTCCGAGGTATT	qPCR	Universal, complementary to SLO sequence
**miR172_Fw**	GGCGGAGAATCTTGATGATG	qPCR	
**miR172a*_Fw**	GGCGGGTGGCATCATCAAGA	qPCR	
**miR165a_Fw**	GGCGGTCGGACCAGGCTTCA	qPCR	
**miR397a_Fw**	GGCGGTCATTGAGTGCAKCG	qPCR	Complementary to both variants
**LAC17_Fw**	GAATTTCGACCCGAACAAGG	qPCR	
**LAC17_Rv**	CCAAGTGACAGTGCATGAAC	qPCR	
**RNA adapter**	CGACUGGAGCACGAGGACACUGACA UGGACUGAAGGAGUAGAAA	5´end RNA ligation	Gene Racer RNA oligonucleotide
**Specific RT**	GTGCGCAATGAAACTGATGC	Reverse transcription, 5' RACE	complementary to 3' region of transcripts derived from CHF3 vector
**5'RACE_Fw1**	CGACTGGAGCACGAGGACACTGA	5'RACE, PCR1	Used for heminested PCRs together with Specific RT oligonucleotide as reverse primer
**5'RACE_Fw2**	GGACACTGACATGGACTGAAGGAGTA	5'RACE, PCR2	
**miR164**	TGCACGTGCCCTGCTTCTCCA	probe	
**miR165**	GGGGGATGAAGCCTGGTCCGA	probe	
**miR172a_19nt**	TGCAGCATCATCAAGATTC	probe	
**miR172a***	TGTGAATCTTGATGATGCCAC	probe	
**miR397a**	CANCAACGCTGCACTCAATGA	probe	
**snRNA U6**	CTCGATTTATGCGTGTCATCCTTGC	probe	
**MIR172a+T7_Fw**	CTGTAATACGACTCACTATAggagcca cggtcgttgttgg	PCR to generate template for *in vitro* transcription	
**MIR172a_Rv**	GGAAAGAATAGTCGTTGATTGC		

Supplementary Table 3: Vector sequences generated and used in this study.

Vector	Lab code	Expressed sequence
**Empty vector**	CHF3	AACACGGGGGACGAGCTCGGTACCCGGGGATCCTCTAGAGTCGACCTGCAG
***Prom35S:MIR172a* wt**	NB40	AACACGGGGGACGAGCTCCCGGAGCCACGGTCGTTGTTGGCTGCTGTGGCATCATCAAGATTCACATCT<br>GTTGATGGACGGTGGTGATTCACTCTCCACAAAGTTCTCTATGAAAATGAGAATCTTGATGATGCTGCAT<br>CGGCAATCAACGACTATTCTTTCCGGATCC
***Prom35S:MIR172a* 1AU**	AR21	AACACGGGGGACGAGCTCCCGGAGCCACGGTCGTTGTTGGCTGATGTGGCATCATCAAGATTCACATCT<br>GTTGATGGACGGTGGTGATTCACTCTCCACAAAGTTCTCTATGAAAATGAGAATCTTGATGATGCTGCAT<br>CGGCAATCAACGACTATTCTTTCCGGATCC
***Prom35S:MIR172a* 1UA**	AR23	AACACGGGGGACGAGCTCCCGGAGCCACGGTCGTTGTTGGCTGTTGTGGCATCATCAAGATTCACATCT<br>GTTGATGGACGGTGGTGATTCACTCTCCACAAAGTTCTCTATGAAAATGAGAATCTTGATGATGCTGCAA<br>CGGCAATCAACGACTATTCTTTCCGGATCC
***Prom35S:MIR172a* 1GC**	AR75	AACACGGGGGACGAGCTCCCGGAGCCACGGTCGTTGTTGGCTGCTGTGGCATCATCAAGATTCACATCT<br>GTTGATGGACGGTGGTGATTCACTCTCCACAAAGTTCTCTATGAAAATGAGAATCTTGATGATGCTGCAG<br>CGGCAATCAACGACTATTCTTTCCGGATCC
***Prom35S:MIR172a* 1CG**	ALS11	AACACGGGGGACGAGCTCCCGGAGCCACGGTCGTTGTTGGCTGGTGTGGCATCATCAAGATTCACATCT<br>GTTGATGGACGGTGGTGATTCACTCTCCACAAAGTTCTCTATGAAAATGAGAATCTTGATGATGCTGCAC<br>CGGCAATCAACGACTATTCTTTCCGGATCC
***Prom35S:MIR172a* 1GU**	AR73	AACACGGGGGACGAGCTCCCGGAGCCACGGTCGTTGTTGGCTGGTGTGGCATCATCAAGATTCACATCT<br>GTTGATGGACGGTGGTGATTCACTCTCCACAAAGTTCTCTATGAAAATGAGAATCTTGATGATGCTGCAT<br>CGGCAATCAACGACTATTCTTTCCGGATCC
***Prom35S:MIR172a* 1UG**	EB41	AACACGGGGGACGAGCTCCCGGAGCCACGGTCGTTGTTGGCTGTTGTGGCATCATCAAGATTCACATCT<br>GTTGATGGACGGTGGTGATTCACTCTCCACAAAGTTCTCTATGAAAATGAGAATCTTGATGATGCTGCAG<br>CGGCAATCAACGACTATTCTTTCCGGATCC
***Prom35S:MIR172a* 1UC**	AR22	AACACGGGGGACGAGCTCCCGGAGCCACGGTCGTTGTTGGCTGTTGTGGCATCATCAAGATTCACATCT<br>GTTGATGGACGGTGGTGATTCACTCTCCACAAAGTTCTCTATGAAAATGAGAATCTTGATGATGCTGCAC<br>CGGCAATCAACGACTATTCTTTCCGGATCC
***Prom35S:MIR172a* 1AC**	AR24	AACACGGGGGACGAGCTCCCGGAGCCACGGTCGTTGTTGGCTGATGTGGCATCATCAAGATTCACATCT<br>GTTGATGGACGGTGGTGATTCACTCTCCACAAAGTTCTCTATGAAAATGAGAATCTTGATGATGCTGCAC<br>CGGCAATCAACGACTATTCTTTCCGGATCC
***Prom35S:MIR172a* 1CA**	EB39	AACACGGGGGACGAGCTCCCGGAGCCACGGTCGTTGTTGGCTGCTGTGGCATCATCAAGATTCACATCT<br>GTTGATGGACGGTGGTGATTCACTCTCCACAAAGTTCTCTATGAAAATGAGAATCTTGATGATGCTGCAA<br>CGGCAATCAACGACTATTCTTTCCGGATCC
***Prom35S:MIR172a* 1AG**	AR25	AACACGGGGGACGAGCTCCCGGAGCCACGGTCGTTGTTGGCTGATGTGGCATCATCAAGATTCACATCT<br>GTTGATGGACGGTGGTGATTCACTCTCCACAAAGTTCTCTATGAAAATGAGAATCTTGATGATGCTGCAG<br>CGGCAATCAACGACTATTCTTTCCGGATCC
***Prom35S:MIR172a* 1GA**	AR20	AACACGGGGGACGAGCTCCCGGAGCCACGGTCGTTGTTGGCTGGTGTGGCATCATCAAGATTCACATCT<br>GTTGATGGACGGTGGTGATTCACTCTCCACAAAGTTCTCTATGAAAATGAGAATCTTGATGATGCTGCAA<br>CGGCAATCAACGACTATTCTTTCCGGATCC
***Prom35S:MIR172a* 1AA**	AR26	AACACGGGGGACGAGCTCCCGGAGCCACGGTCGTTGTTGGCTGATGTGGCATCATCAAGATTCACATCT<br>GTTGATGGACGGTGGTGATTCACTCTCCACAAAGTTCTCTATGAAAATGAGAATCTTGATGATGCTGCAA<br>CGGCAATCAACGACTATTCTTTCCGGATCC
***Prom35S:MIR172a* 1GG**	EB40	AACACGGGGGACGAGCTCCCGGAGCCACGGTCGTTGTTGGCTGGTGTGGCATCATCAAGATTCACATCT<br>GTTGATGGACGGTGGTGATTCACTCTCCACAAAGTTCTCTATGAAAATGAGAATCTTGATGATGCTGCAG<br>CGGCAATCAACGACTATTCTTTCCGGATCC
***Prom35S:MIR172a* 1UU**	BM65	AACACGGGGGACGAGCTCCCGGAGCCACGGTCGTTGTTGGCTGTTGTGGCATCATCAAGATTCACATCT<br>GTTGATGGACGGTGGTGATTCACTCTCCACAAAGTTCTCTATGAAAATGAGAATCTTGATGATGCTGCAG<br>CGGCAATCAACGACTATTCTTTCCGGATCC
***Prom35S:MIR172a* 1CC**	JM71	AACACGGGGGACGAGCTCCCGGAGCCACGGTCGTTGTTGGCTGCTGTGGCATCATCAAGATTCACATCT<br>GTTGATGGACGGTGGTGATTCACTCTCCACAAAGTTCTCTATGAAAATGAGAATCTTGATGATGCTGCAC<br>CGGCAATCAACGACTATTCTTTCCGGATCC
***Prom35S:MIR172a* 6AC**	AR28	AACACGGGGGACGAGCTCCCGGAGCCACGGTCGTTGTTGGCTGCTGTGACATCATCAAGATTCACATCT<br>GTTGATGGACGGTGGTGATTCACTCTCCACAAAGTTCTCTATGAAAATGAGAATCTTGATGATGCTGCAT<br>CGGCAATCAACGACTATTCTTTCCGGATCC
***Prom35S:MIR172a* 6CC**	AR27	AACACGGGGGACGAGCTCCCGGAGCCACGGTCGTTGTTGGCTGCTGTGCCATCATCAAGATTCACATCT<br>GTTGATGGACGGTGGTGATTCACTCTCCACAAAGTTCTCTATGAAAATGAGAATCTTGATGATGCTGCAT<br>CGGCAATCAACGACTATTCTTTCCGGATCC
***Prom35S:MIR172a* -1AC**	JM32	AACACGGGGGACGAGCTCCCGGAGCCACGGTCGTTGTTGGCTACTGTGGCATCATCAAGATTCACATCT<br>GTTGATGGACGGTGGTGATTCACTCTCCACAAAGTTCTCTATGAAAATGAGAATCTTGATGATGCTGCAT<br>CGGCAATCAACGACTATTCTTTCCGGATCC
***Prom35S:MIR172a* -1CC**	AR117	AACACGGGGGACGAGCTCCCGGAGCCACGGTCGTTGTTGGCTCCTGTGGCATCATCAAGATTCACATCT<br>GTTGATGGACGGTGGTGATTCACTCTCCACAAAGTTCTCTATGAAAATGAGAATCTTGATGATGCTGCAT<br>CGGCAATCAACGACTATTCTTTCCGGATCC
***Prom35S:MIR165a* wt**	JD20	AACACGGGGGACGAGCTCATCATTCCCTCATCATAACACCATCATCACCATTCACCAACCTCTCTCTCTCTC<br>TCCTCTATCACTCTCTACAACAAAAATTTGTGAATCTGCTAAGATCGATTATCATGAGGGTTAAGCTATTTC<br>AGTTGAGGGGAATGTTGTCTGGATCGAGGATATTATAGATATATACATGTGTATGTTAATGATTCAAGTG<br>ATCATAGAGAGTATCCTCGGACCAGGCTTCATCCCCCCCAACATGTTATTGCCTCGGATCC
***Prom35S:MIR165a* 1UC**	AR124	AACACGGGGGACGAGCTCATCATTCCCTCATCATAACACCATCATCACCATTCACCAACCTCTCTCTCTCTC<br>TCCTCTATCACTCTCTACAACAAAAATTTGTGAATCTGCTAAGATCGATTATCATGAGGGTTAAGCTATTTC<br>AGTTGATGGGAATGTTGTCTGGATCGAGGATATTATAGATATATACATGTGTATGTTAATGATTCAAGTG<br>ATCATAGAGAGTATCCTCGGACCAGGCTTCATCCCCCCCAACATGTTATTGCCTCGGATCC
***Prom35S:MIR165a* 1CC**	AR96	AACACGGGGGACGAGCTCATCATTCCCTCATCATAACACCATCATCACCATTCACCAACCTCTCTCTCTCTC<br>TCCTCTATCACTCTCTACAACAAAAATTTGTGAATCTGCTAAGATCGATTATCATGAGGGTTAAGCTATTTC<br>AGTTGACGGGAATGTTGTCTGGATCGAGGATATTATAGATATATACATGTGTATGTTAATGATTCAAGTG<br>ATCATAGAGAGTATCCTCGGACCAGGCTTCATCCCCCCCAACATGTTATTGCCTCGGATCC

***Prom35S:MIR164c* wt**	SVS43	AACACGGGGGACGAGCTCGAGAGAAAGTAATGGGTGAGTAACACTTGATGGAGAAGCAGGGCACGTG CGAACACAAATGAAATCGATCGGTACTTGTTGATCATATTTTCGCACGTGTTCTACTACTCCAACACGTGT CTCTCCCCCTCCCCGAGGATGGATCC
***Prom35S:MIR164c* 23AC**	AR43	AACACGGGGGACGAGCTCGAGAGAAAGTAATGGGTGAGTAACACTTGATGGAGAAGCAGGGCACGTG CAAACACAAATGAAATCGATCGGTACTTGTTGATCATATTTTCGCACGTGTTCTACTACTCCAACACGTGT CTCTCCCCCTCCCCGAGGATGGGATCC
***Prom35S:MIR164c* 23UC**	JM84	AACACGGGGGACGAGCTCGAGAGAAAGTAATGGGTGAGTAACACTTGATGGAGAAGCAGGGCACGTG CTAACACAAATGAAATCGATCGGTACTTGTTGATCATATTTTCGCACGTGTTCTACTACTCCAACACGTGT CTCTCCCCCTCCCCGAGGATGGGATCC
***Prom35S:MIR164c* 23GU**	JM81	AACACGGGGGACGAGCTCGAGAGAAAGTAATGGGTGAGTAACACTTGATGGAGAAGCAGGGCACGTG CGAACACAAATGAAATCGATCGGTACTTGTTGATCATATTTTTGCACGTGTTCTACTACTCCAACACGTGT CTCTCCCCCTCCCCGAGGATGGGATCC
***Prom35S:MIR164c* 23CC**	JM73	AACACGGGGGACGAGCTCGAGAGAAAGTAATGGGTGAGTAACACTTGATGGAGAAGCAGGGCACGTG CCAACACAAATGAAATCGATCGGTACTTGTTGATCATATTTTCGCACGTGTTCTACTACTCCAACACGTGT CTCTCCCCCTCCCCGAGGATGGGATCC
***Prom35S:MIR164c* 23GG**	JM72	AACACGGGGGACGAGCTCGAGAGAAAGTAATGGGTGAGTAACACTTGATGGAGAAGCAGGGCACGTG CGAACACAAATGAAATCGATCGGTACTTGTTGATCATATTTTGGCACGTGTTCTACTACTCCAACACGTGT CTCTCCCCCTCCCCGAGGATGGGATCC
***Prom35S:MIR397a* wt**	RER87	AACACGGGGGACGAGCTCGGTACCAGGATATTTTTTCCTGGGTTTGAATGAACATCATTGAGTGCAGCGT TGATGTAATTTCGTTTTGTTTTTCATTGTTGAATGGATTAAAAGAATTTATACCAGCGTTGCGCTCAATTAT GTTTTTCTAATTTTCAGGATCC
***Prom35S:MIR397a* 21CC**	NB8	AACACGGGGGACGAGCTCGGTACCAGGATATTTTTTCCTGGGTTTGAATGAACATCATTGAGTGCAGCGT TGCTGTAATTTCGTTTTGTTTTTCATTGTTGAATGGATTAAAAGAATTTATACCAGCGTTGCGCTCAATTAT GTTTTTCTAATTTTCAGGATCC
***Prom35S:MIR397a* 21AG**	NB6	AACACGGGGGACGAGCTCGGTACCAGGATATTTTTTCCTGGGTTTGAATGAACATCATTGAGTGCAGCGT TGATGTAATTTCGTTTTGTTTTTCATTGTTGAATGGATTAAAAGAATTTATAGCAGCGTTGCGCTCAATTAT GTTTTTCTAATTTTCAGGATCC

Sequences are shown from +1 to the restriction enzyme site used for cloning in 3´end of the precursor.
