# Supplementary material for: Identification of key sequence features required for microRNA biogenesis in plants
Source: Nat Commun. 2020 Oct 21;11:5320. doi: 10.1038/s41467-020-19129-6 (PMC7577975; doi:10.1038/s41467-020-19129-6)

Supplementary Data 1.

Molecular Dynamics simulation of *MIR172A* containing the nucleotide pairs modified at position 1.

A- Representative snapshot of the wt variant of the miR172a model. Carbon atoms are shown in cyan, nitrogen atoms in blue, oxygen atoms in red, phosphorus atoms in brown, sodium ions in pink, chloride ions in green, and hydrogen atoms are omitted.

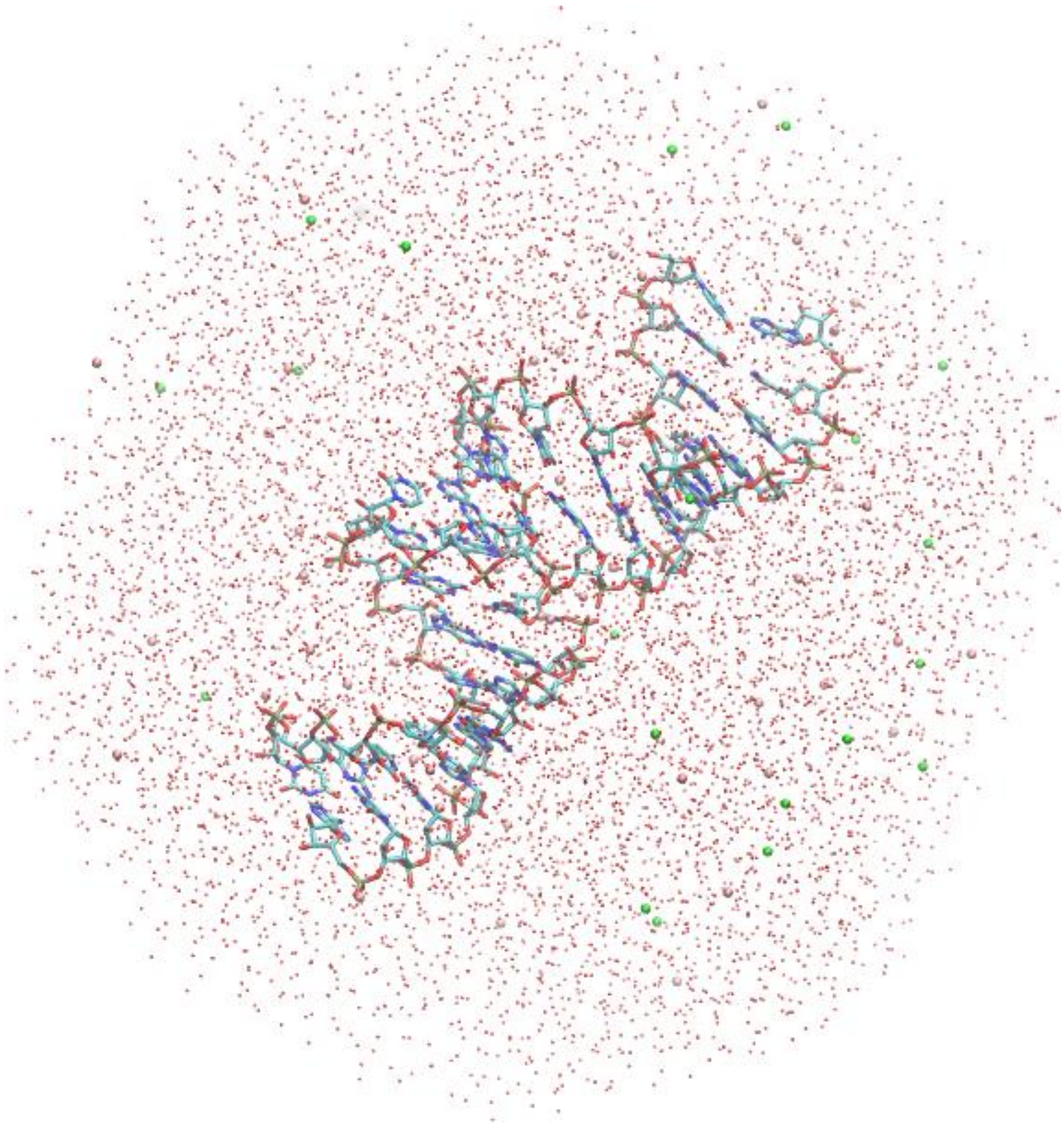

B- Axial bend through the simulation of *MIR172A* variants. Above each graph the nucleotide pair is indicated, wt variant graph is highlighted with a purple rectangle.

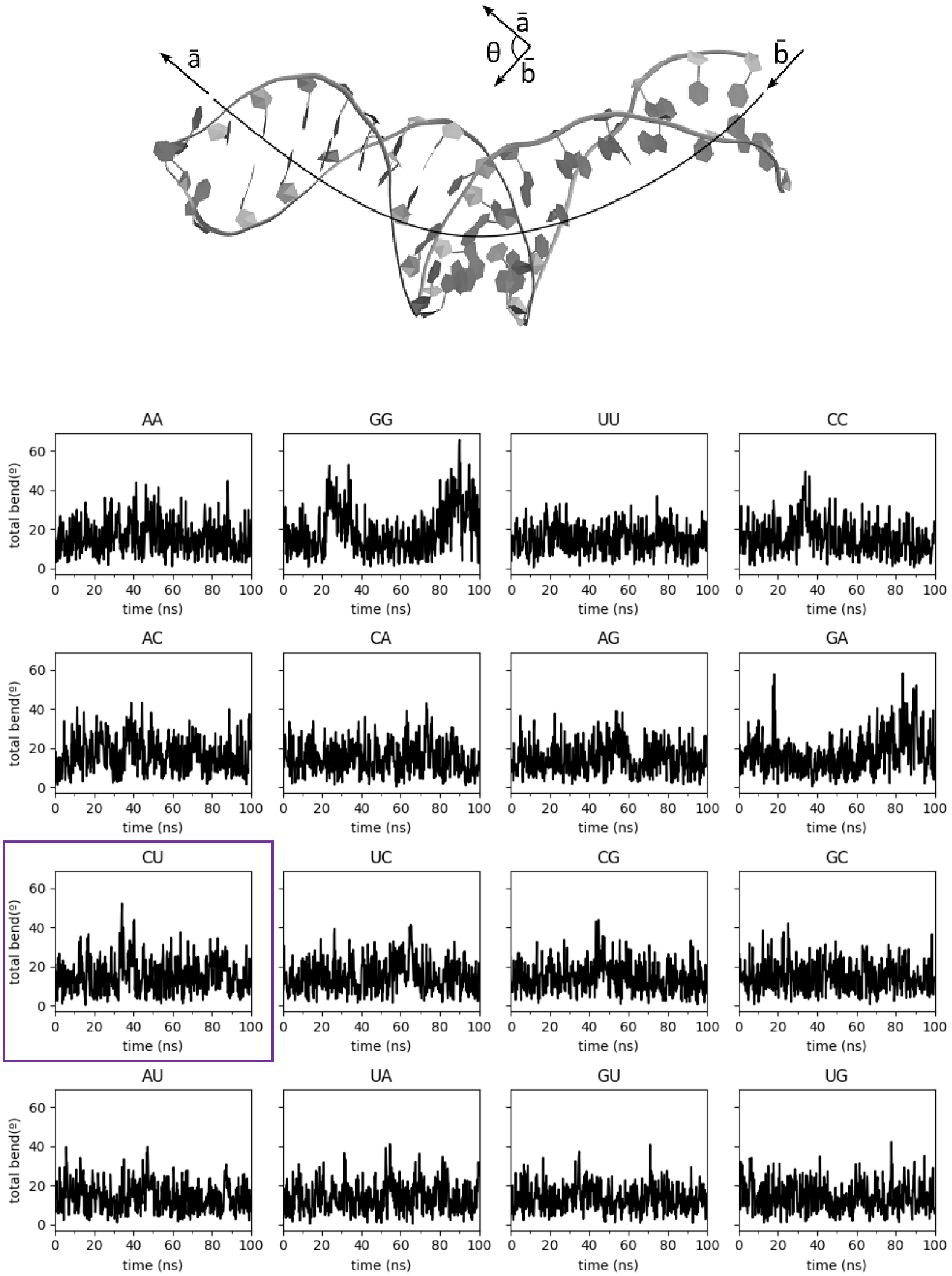

C- Structural parameters for the dsRNA calculated using Curves+.

A segment of *MIR172A* containing the nucleotide pairs modified (corresponding to positions 1) were used for these analyses.

A schematic diagram is depicted representing each parameter based on previous work (Xiang-Jun Lu, Wilma K. Olson, 3DNA: a software package for the analysis, rebuilding and visualization of three-dimensional nucleic acid structures, Nucleic Acids Research, Volume 31, Issue 17, 1 September 2003, Pages 5108–5121, https://doi.org/10.1093/nar/gkg680; Lu, X., Olson, W. 3DNA: a versatile, integrated software system for the analysis, rebuilding and visualization of three-dimensional nucleic-acid structures. Nat Protoc 3, 1213–1227 (2008). https://doi.org/10.1038/nprot.2008.104)

In these diagrams, the shaded edge (facing the viewer) denotes the minor-groove side of a base or base pair as indicated by the author (http://x3dna.org/highlights/schematic-diagrams-of-base-pair-parameters).

The parameters shown correspond to position 1 of the wt and mutated *MIR172A* precursors. The purple rectangle indicates the wt variant graph.

TRASLATIONAL PARAMETERS

Shear parameter.

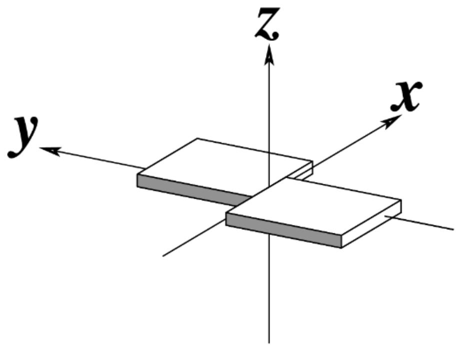

For this precursor and parameter, the MD was performed three times. First column in the graphs correspond to the MD performed with Berendsen thermostat ("simulation 1"), whereas second and third columns graphs were calculated with Langevin thermostat (labeled as "simulation 2" and "simulation 3"). Similar results were obtained.

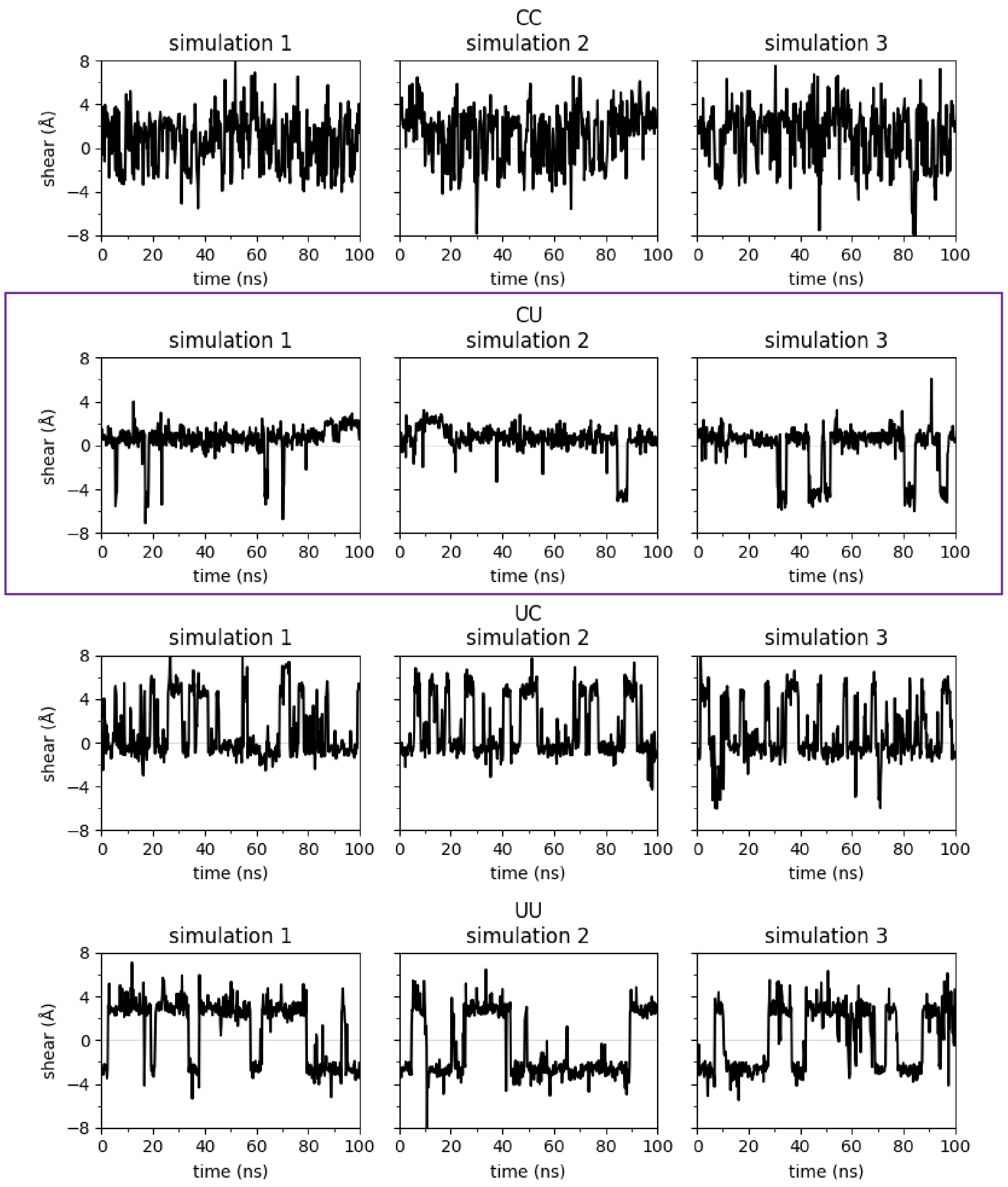
CC
CU
UC
UU
simulation 1
simulation 2
simulation 3
shear (Å)
time (ns)

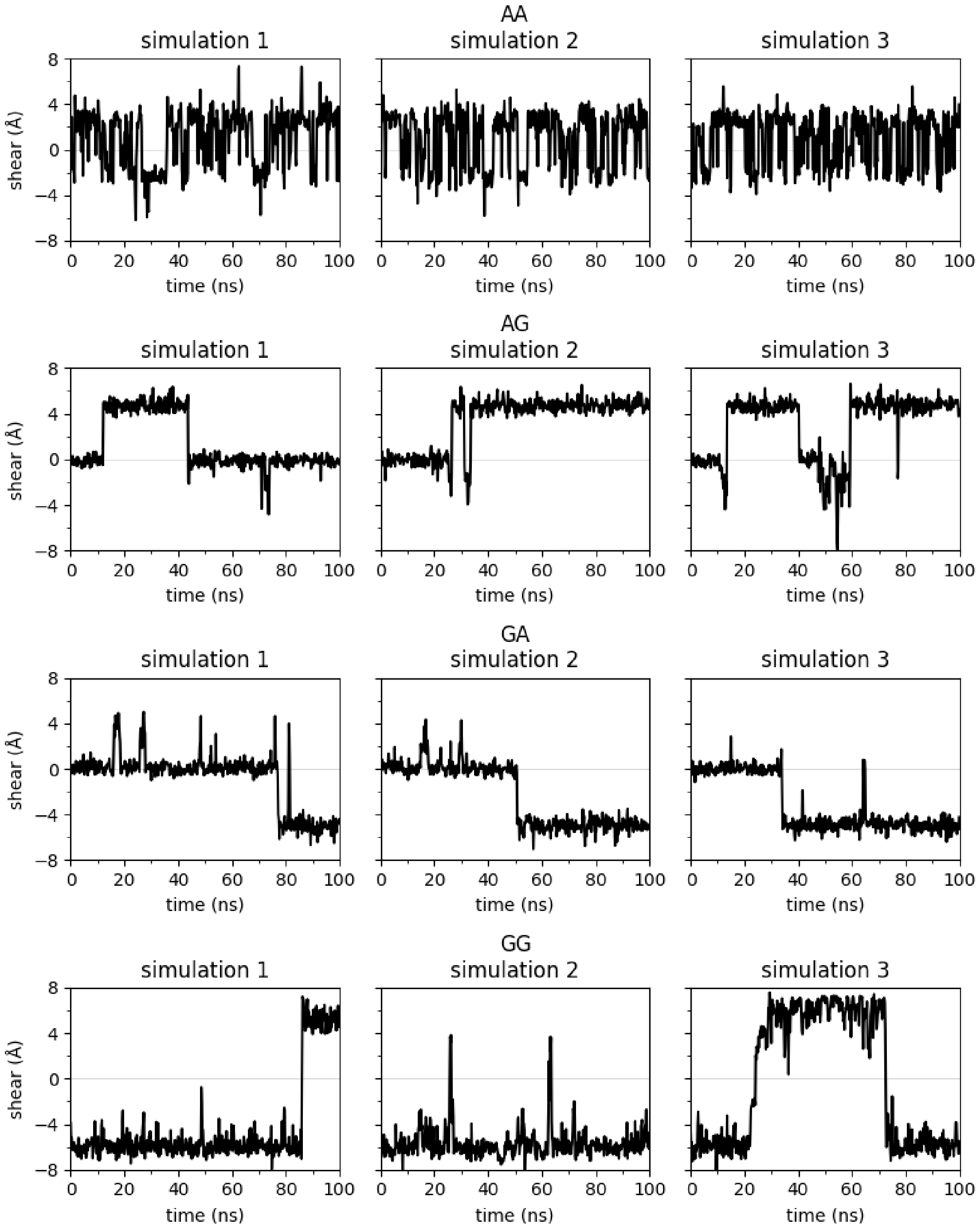
AA
AG
GA
GG
simulation 1
simulation 2
simulation 3
shear (Å)
time (ns)
8
4
0
−4
−8
0
20
40
60
80
100

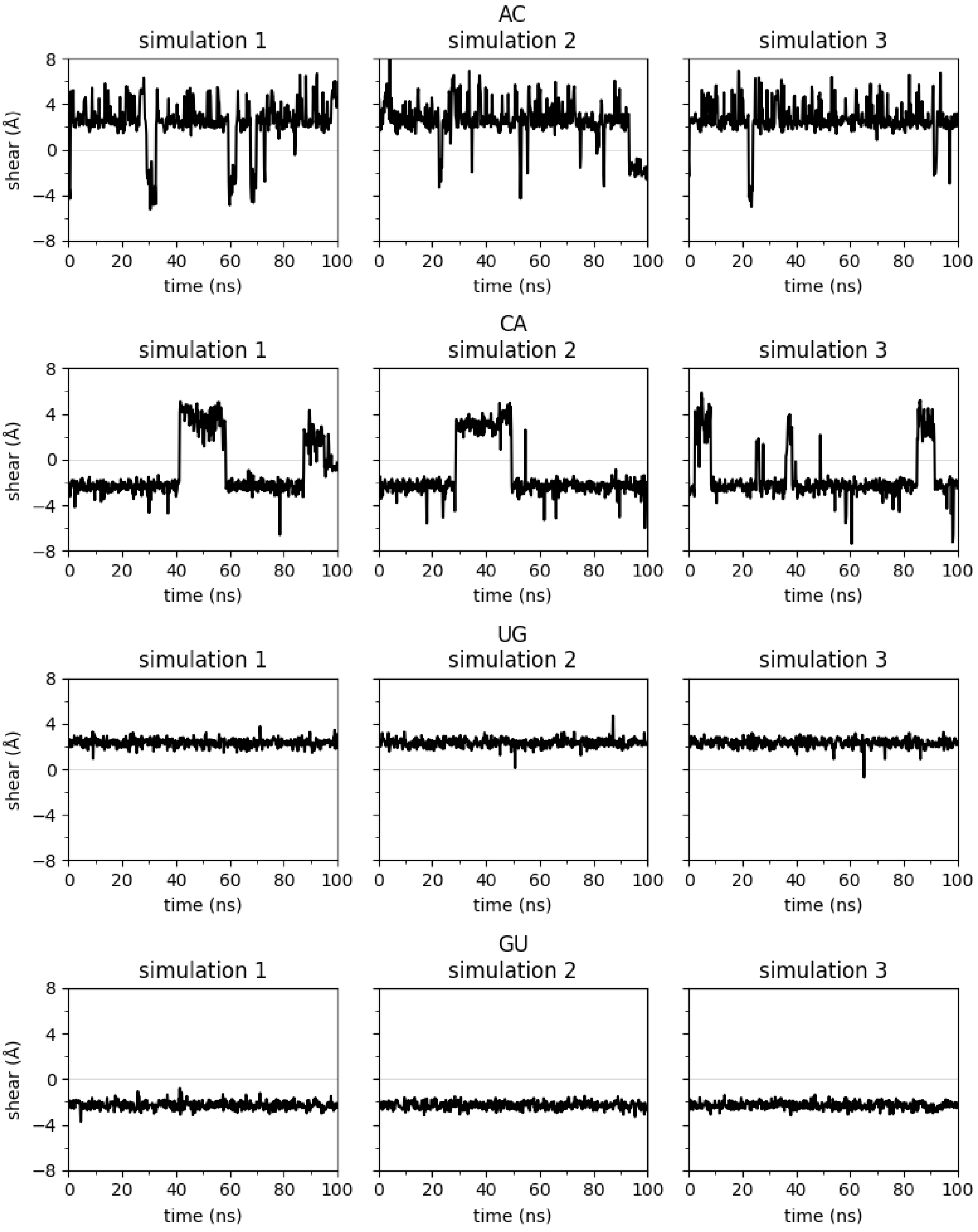
AC
simulation 1
simulation 2
simulation 3
CA
simulation 1
simulation 2
simulation 3
UG
simulation 1
simulation 2
simulation 3
GU
simulation 1
simulation 2
simulation 3
shear (Å)
8
4
0
−4
−8
time (ns)
0
20
40
60
80
100

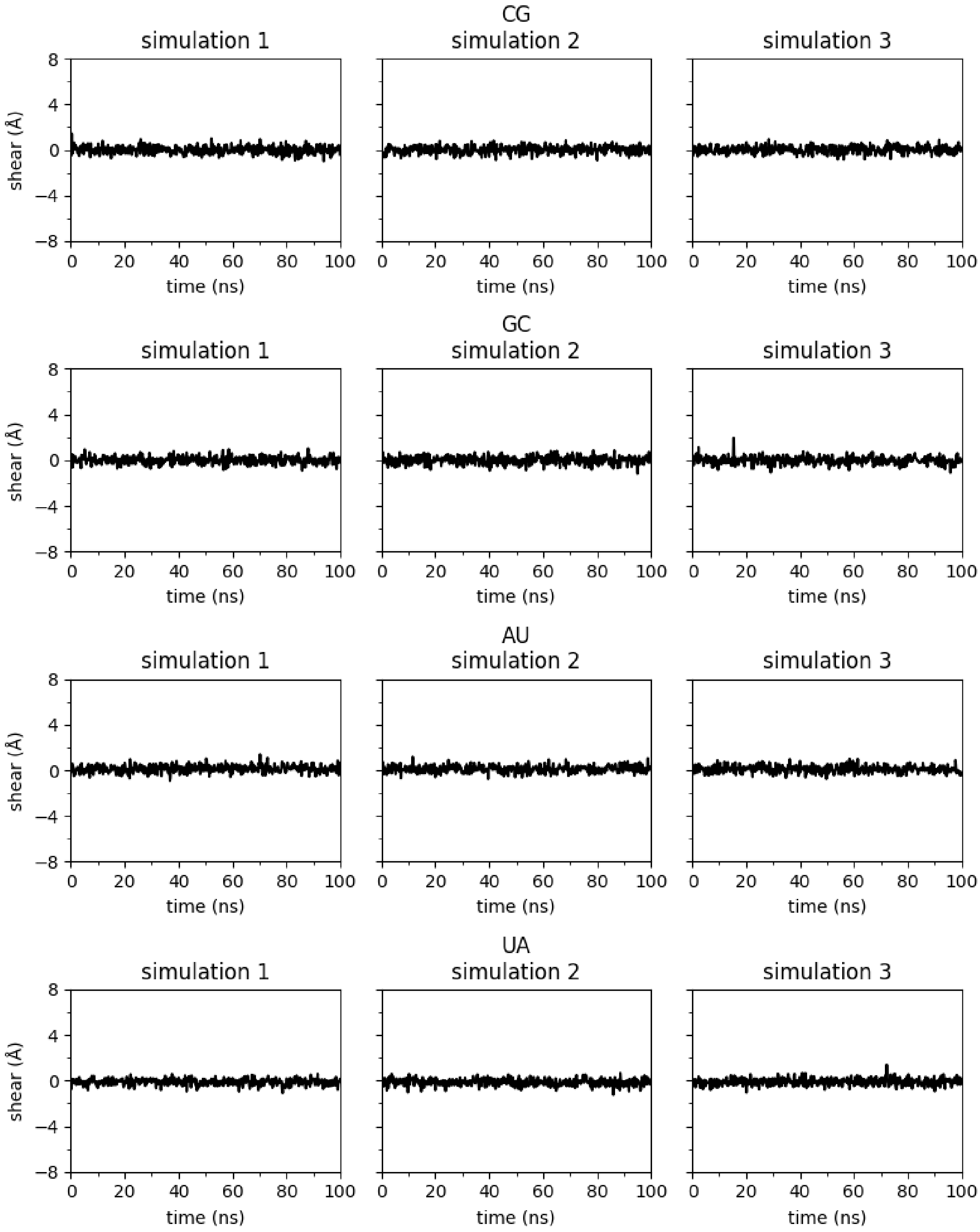
CG
GC
AU
UA
simulation 1
simulation 2
simulation 3
shear (Å)
time (ns)
8
4
0
−4
−8
0
20
40
60
80
100

Stretch parameter.

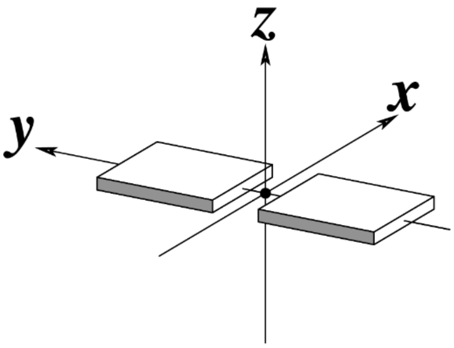

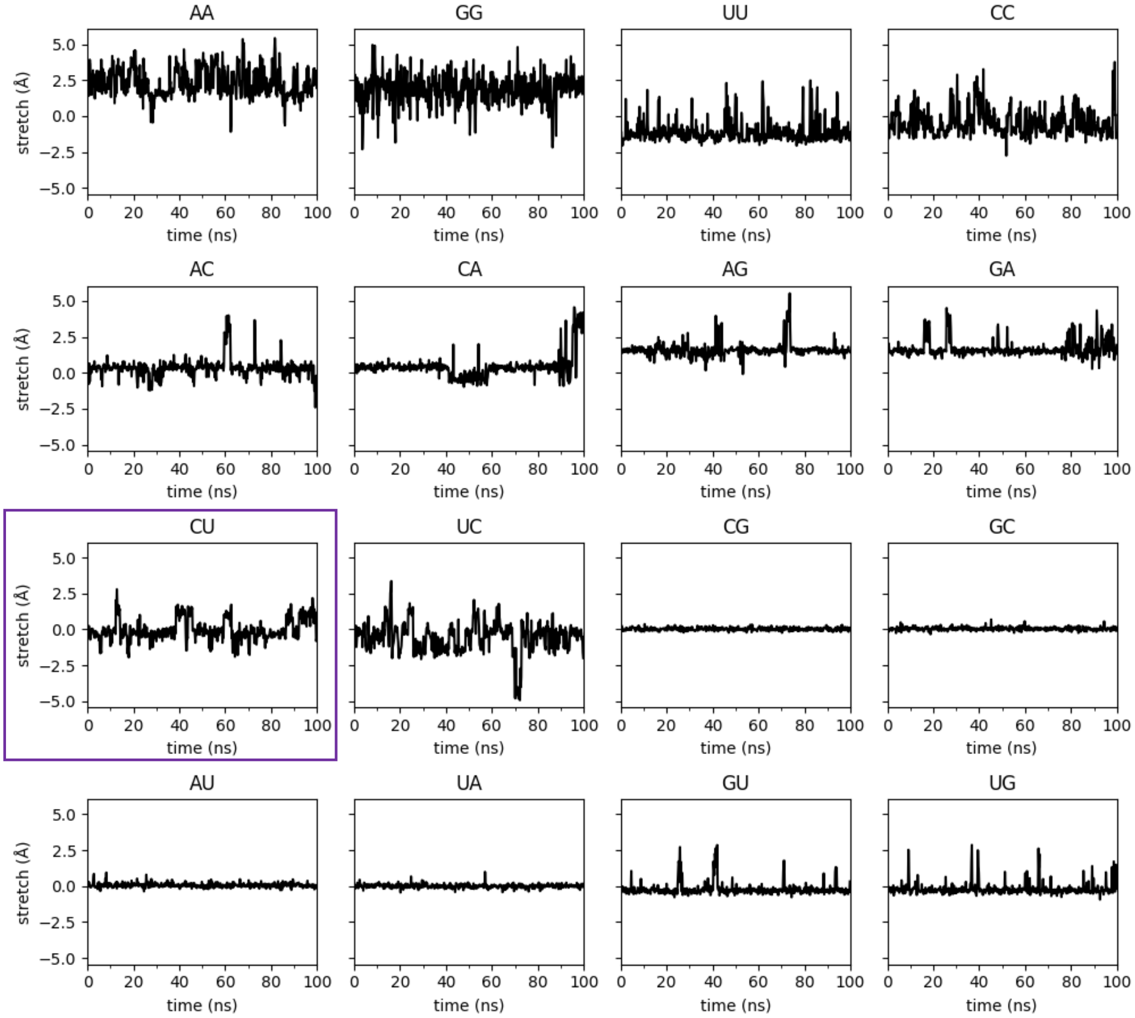

Stagger parameter.

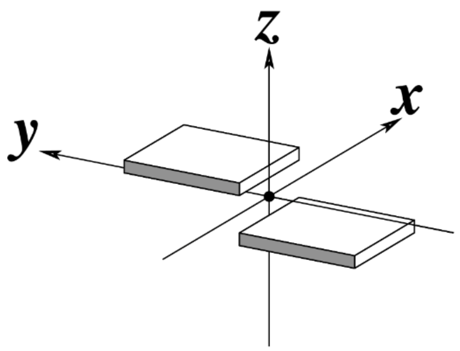

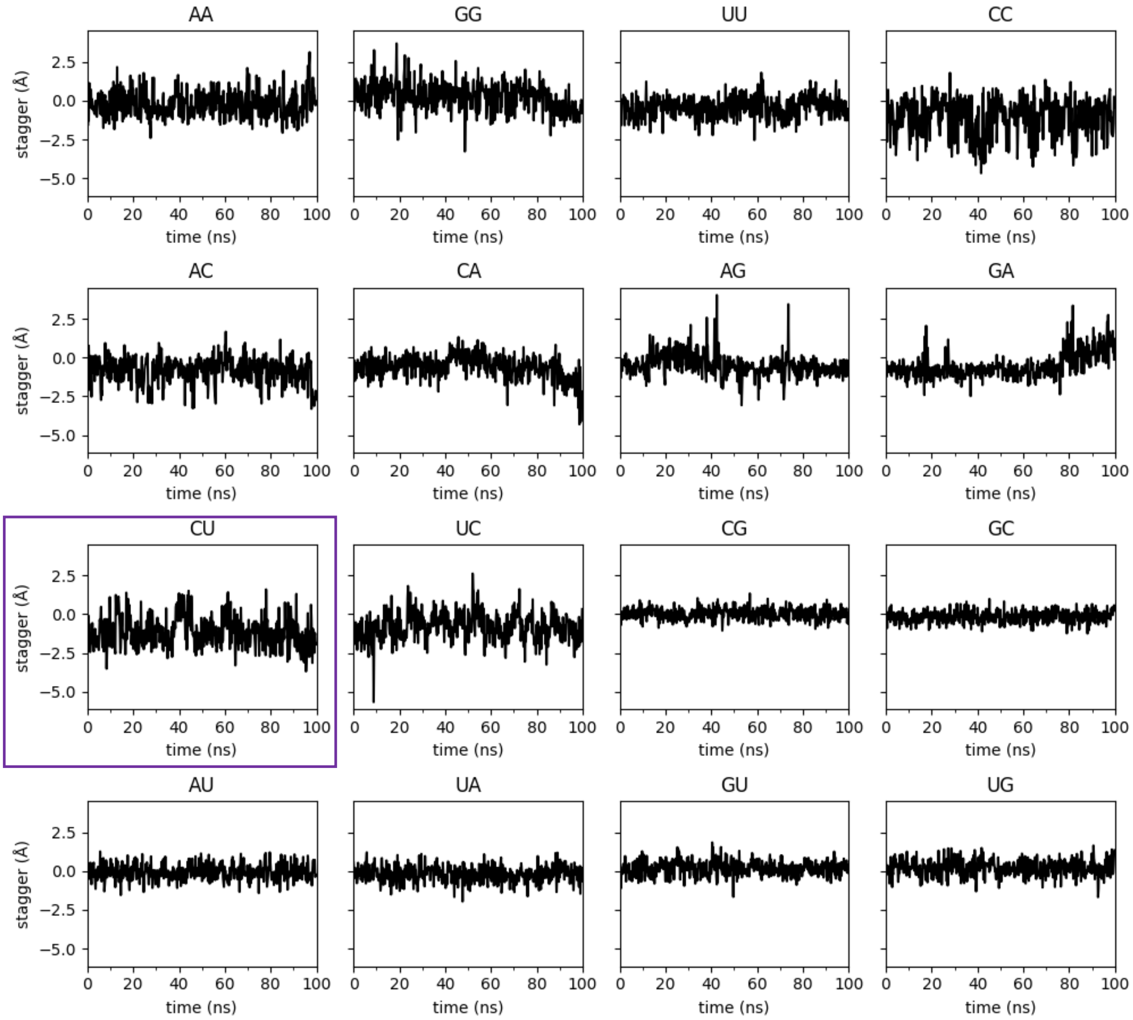

Shift parameter.

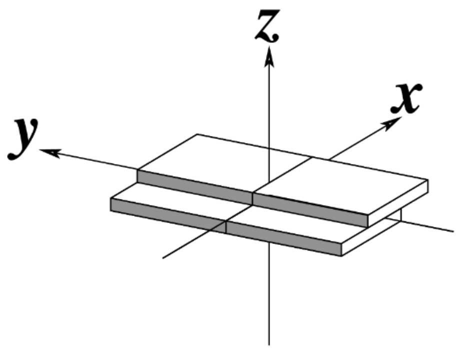

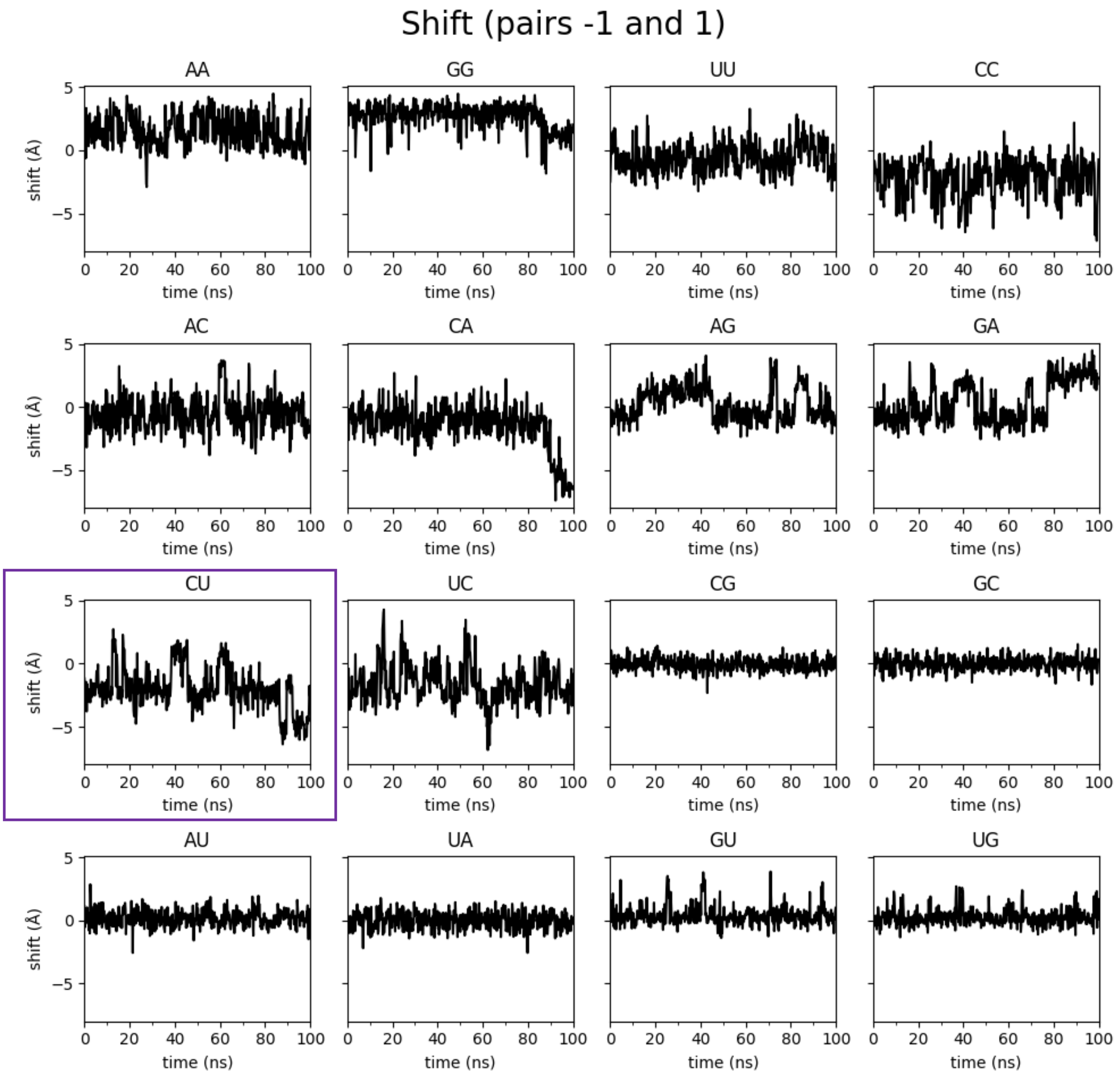

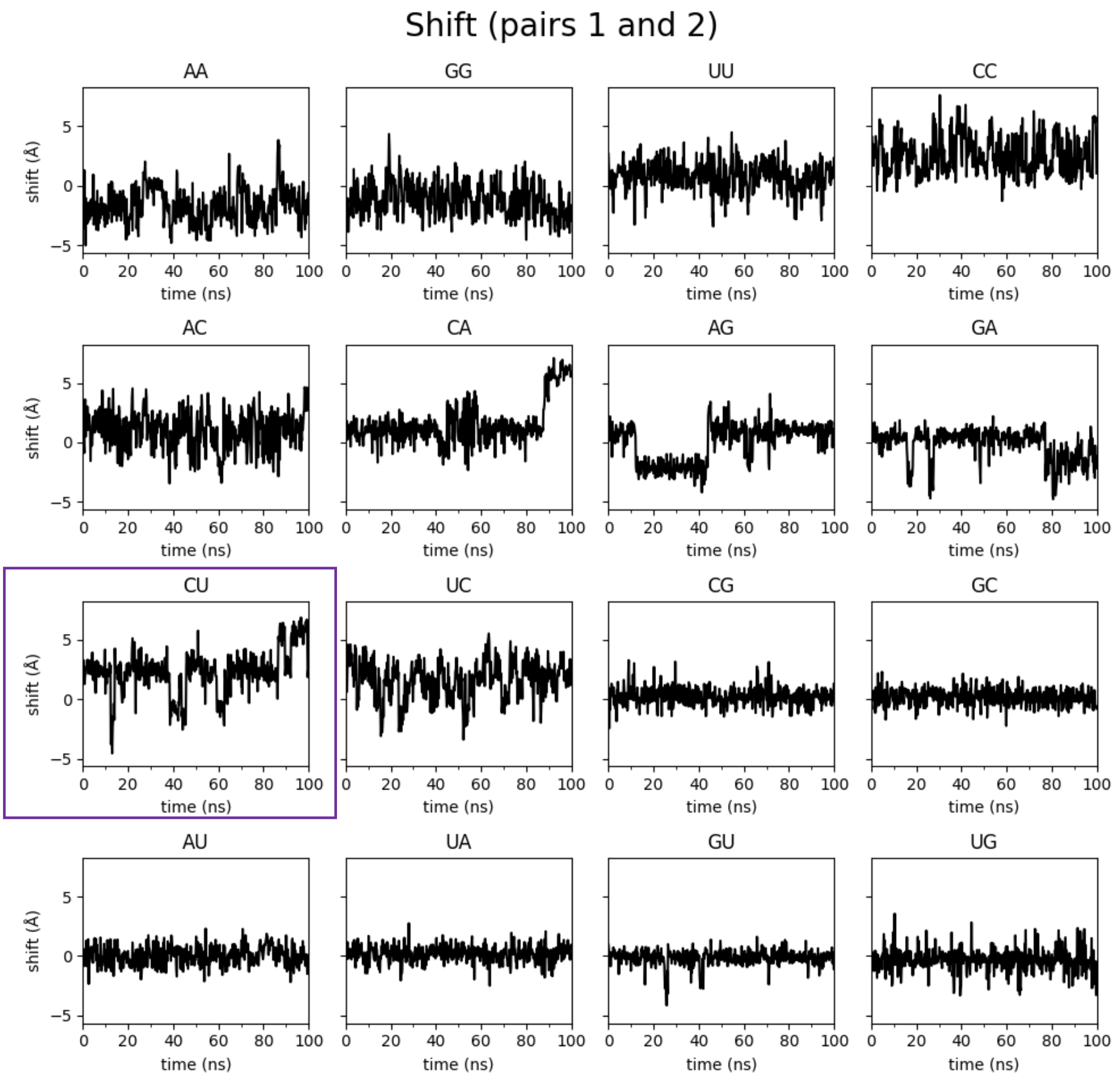
Shift (pairs 1 and 2)
AA
GG
UU
CC
AC
CA
AG
GA
CU
UC
CG
GC
AU
UA
GU
UG
shift (Å)
time (ns)

Slide parameter.

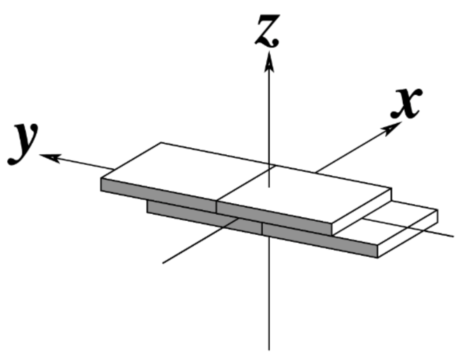

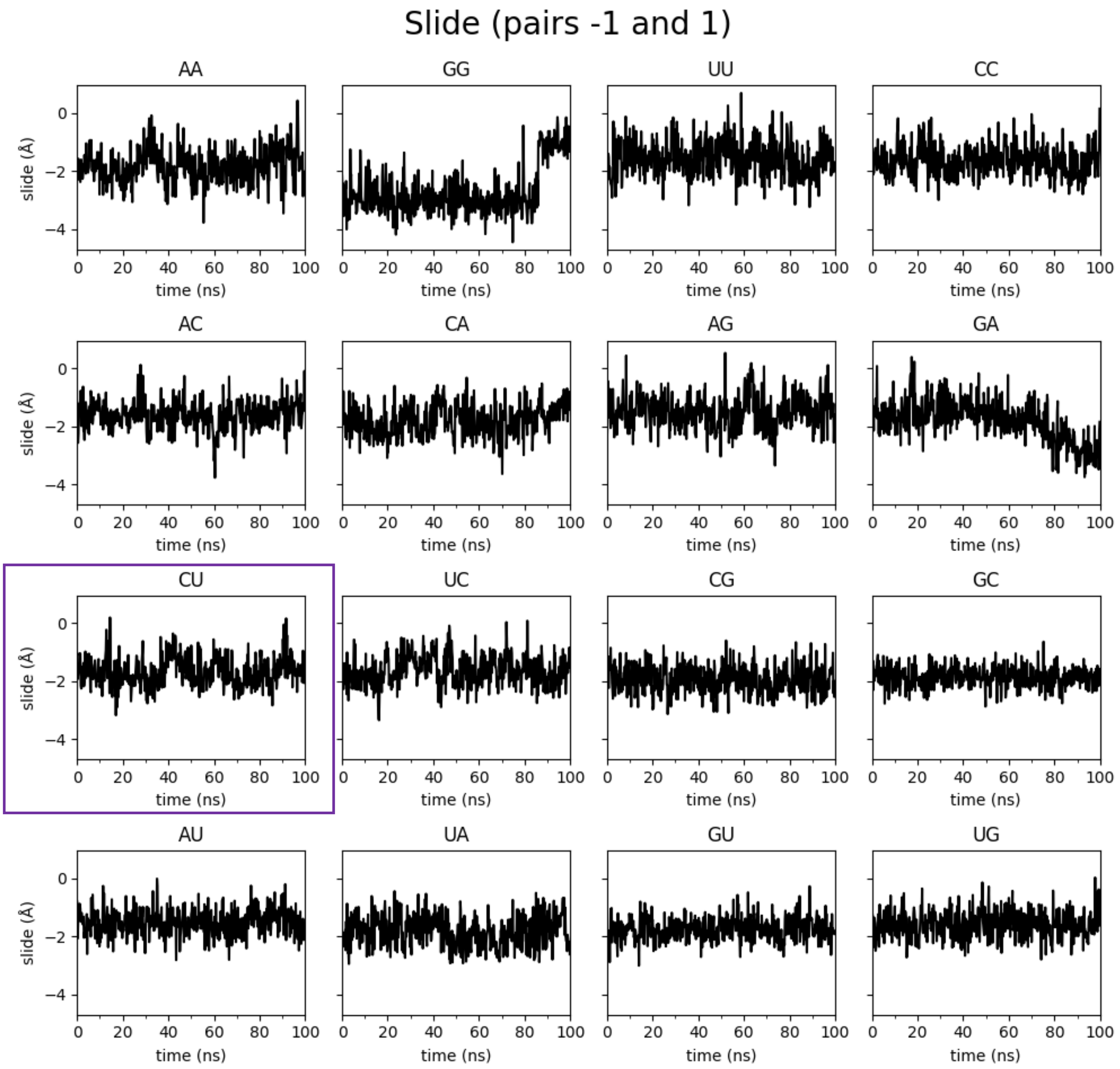

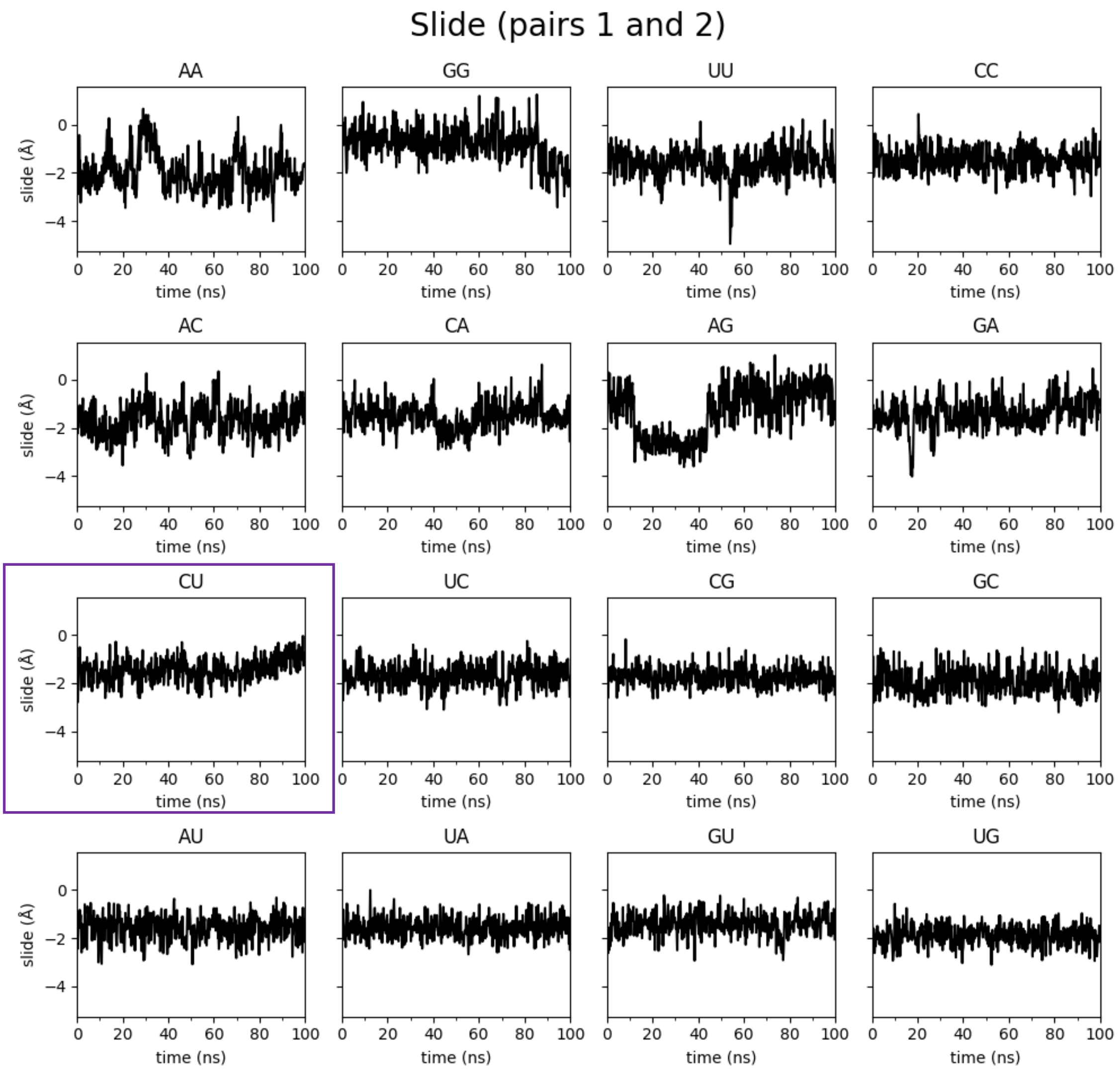
Slide (pairs 1 and 2)
AA
GG
UU
CC
AC
CA
AG
GA
CU
UC
CG
GC
AU
UA
GU
UG
slide (Å)
time (ns)

Rise parameter.

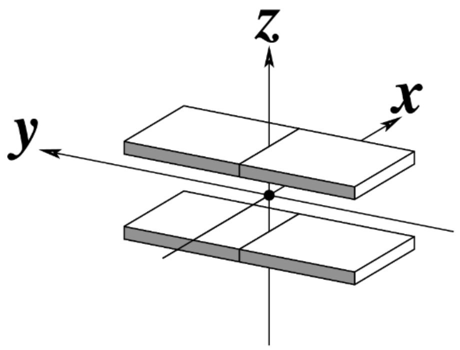

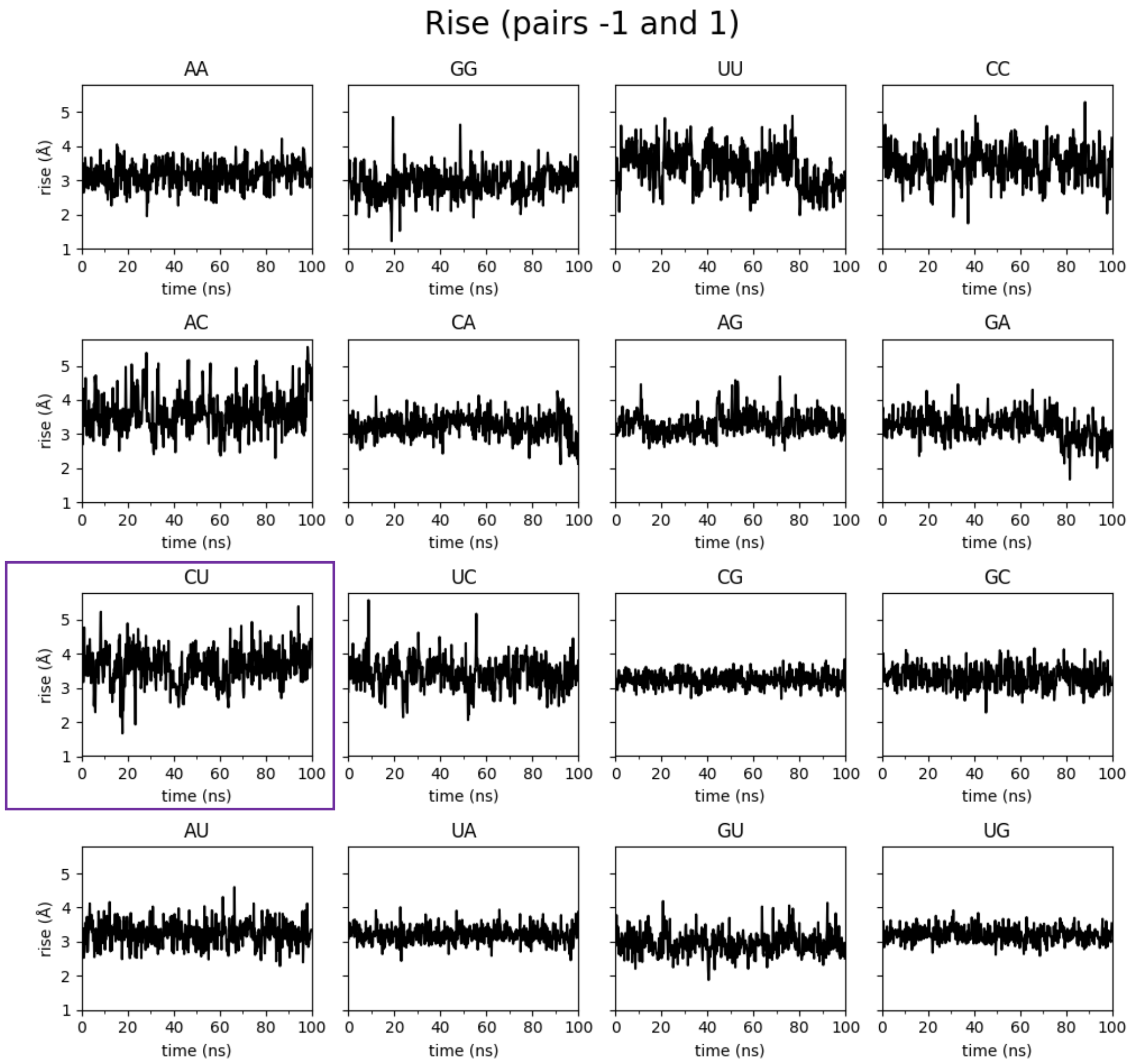

ROTATIONAL PARAMETERS

Buckle parameter.

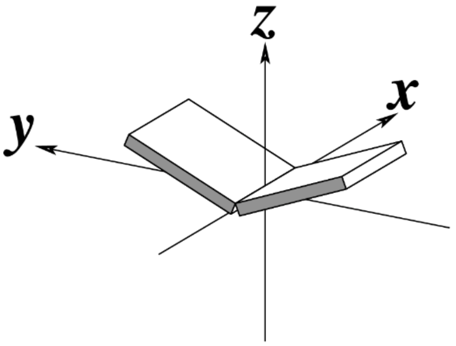

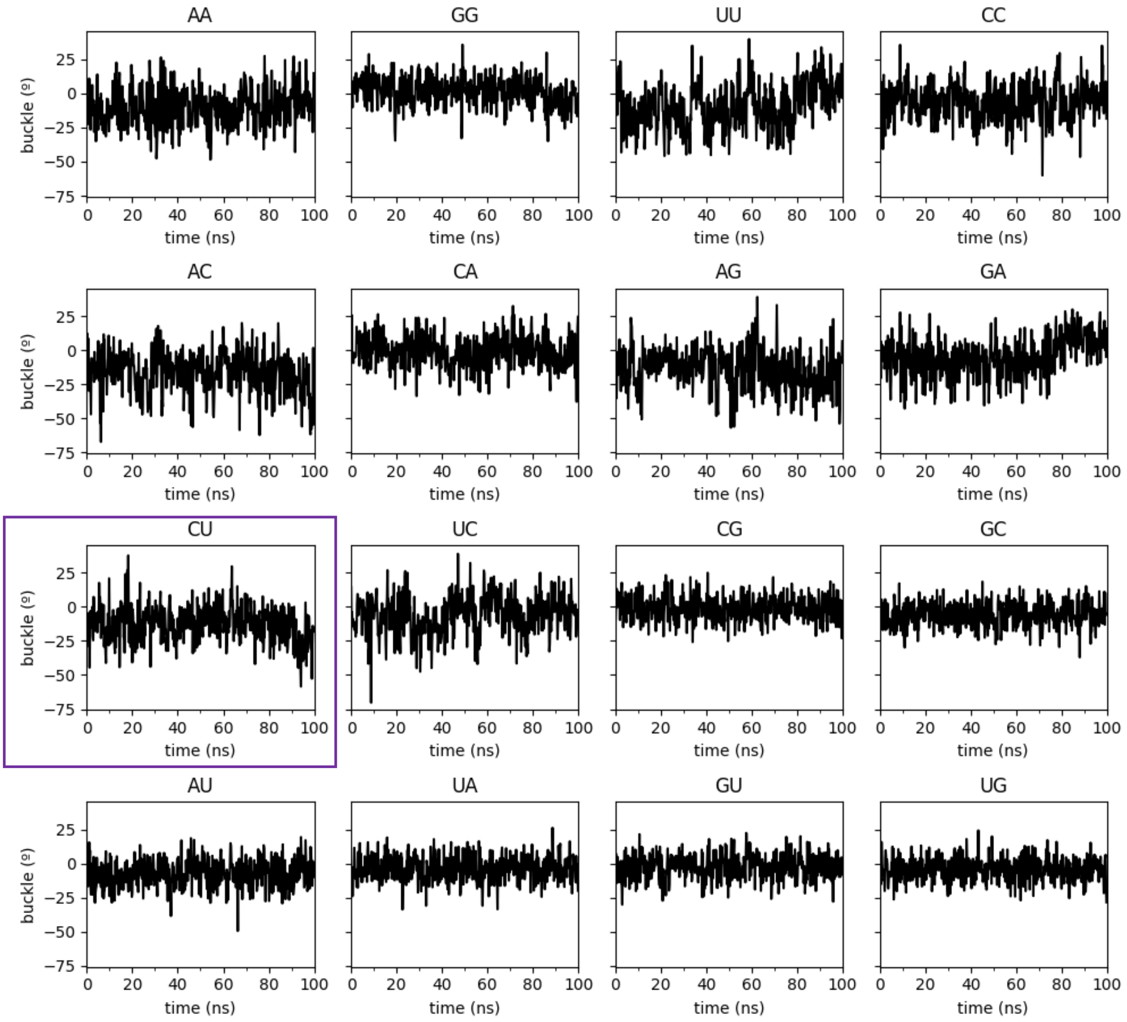

Propeller parameter.

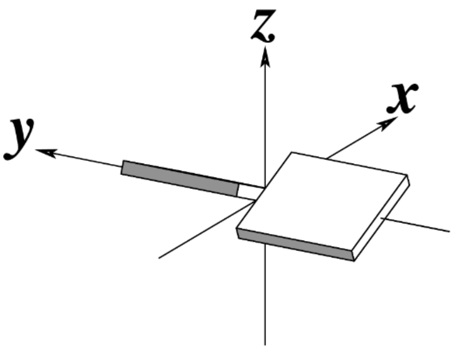

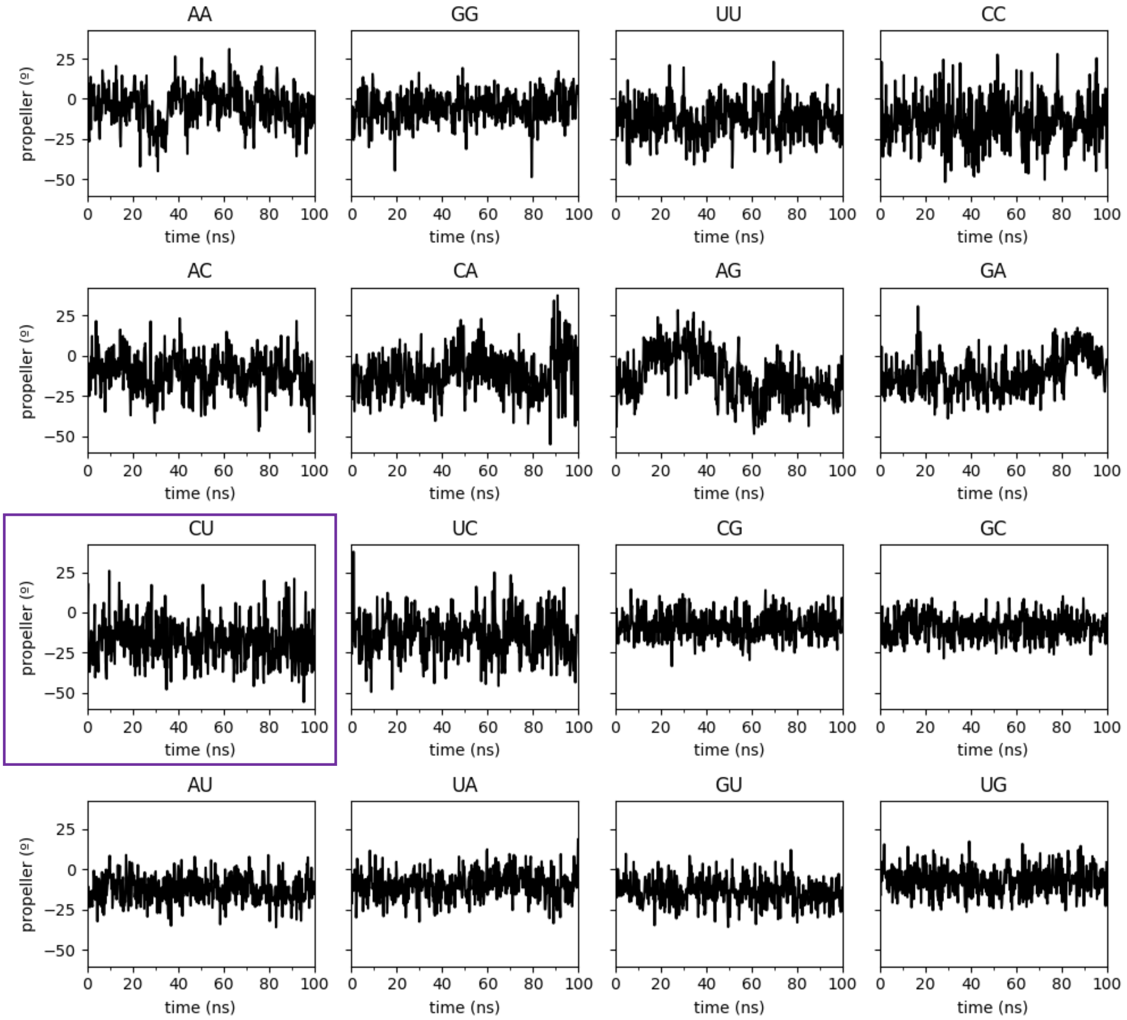

Opening parameter.

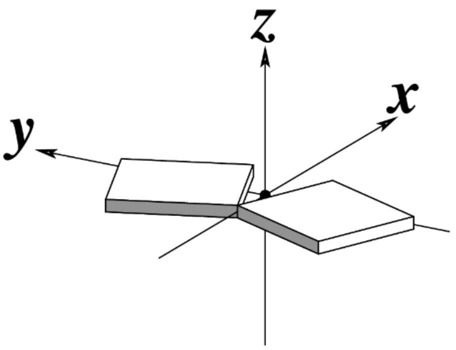

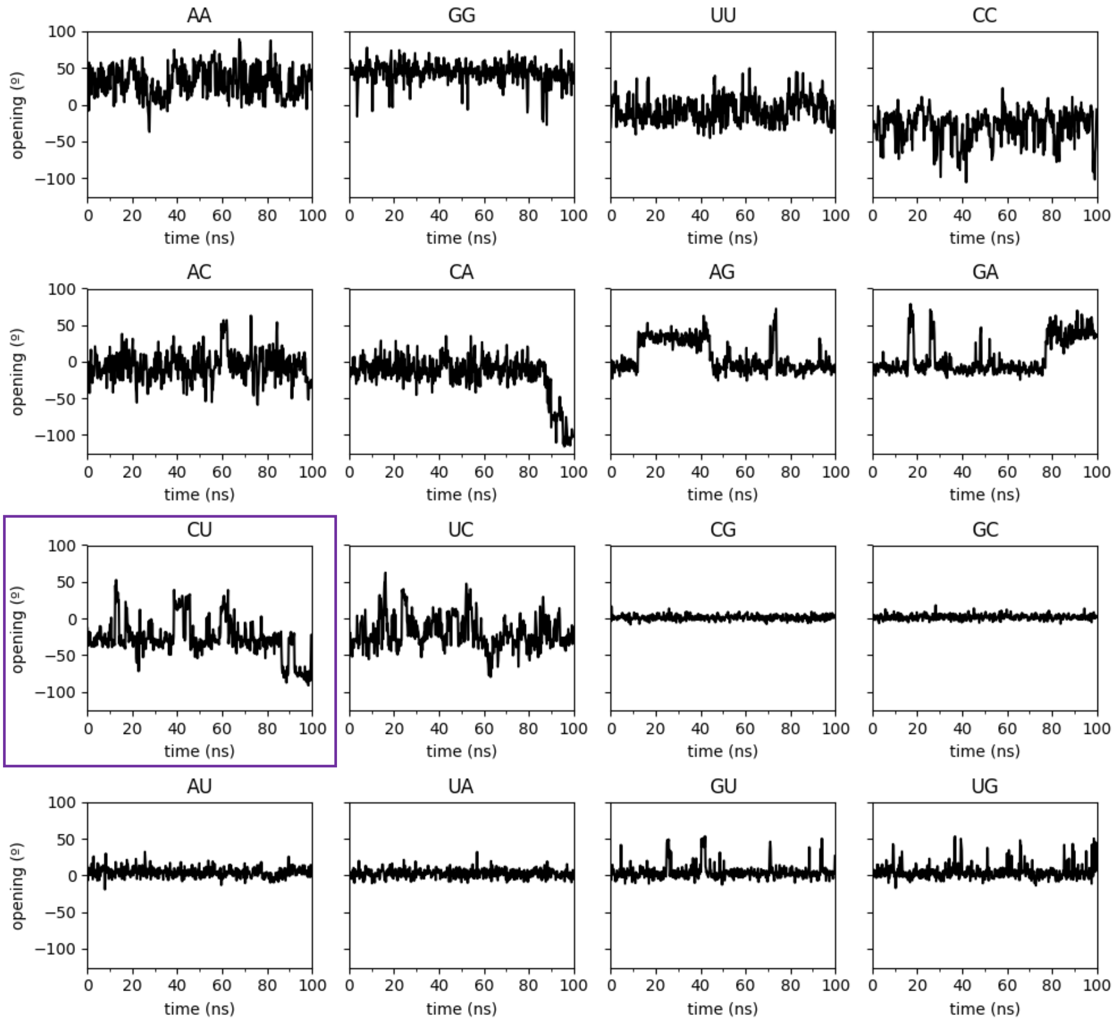

Tilt parameter.

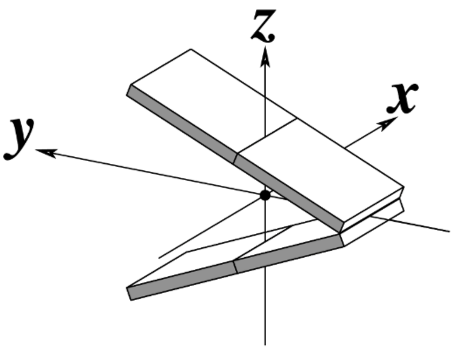

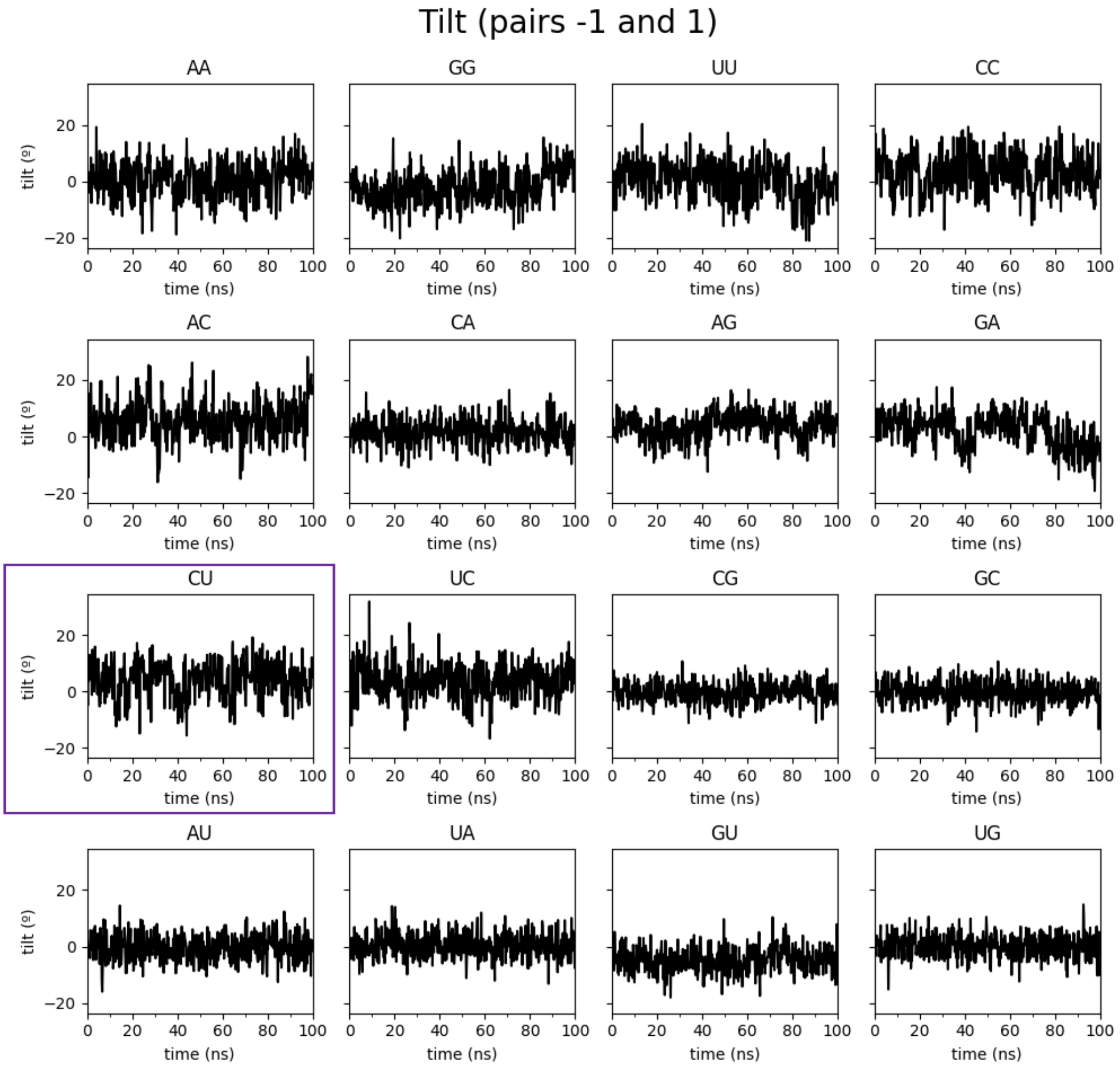

Tilt (pairs 1 and 2)

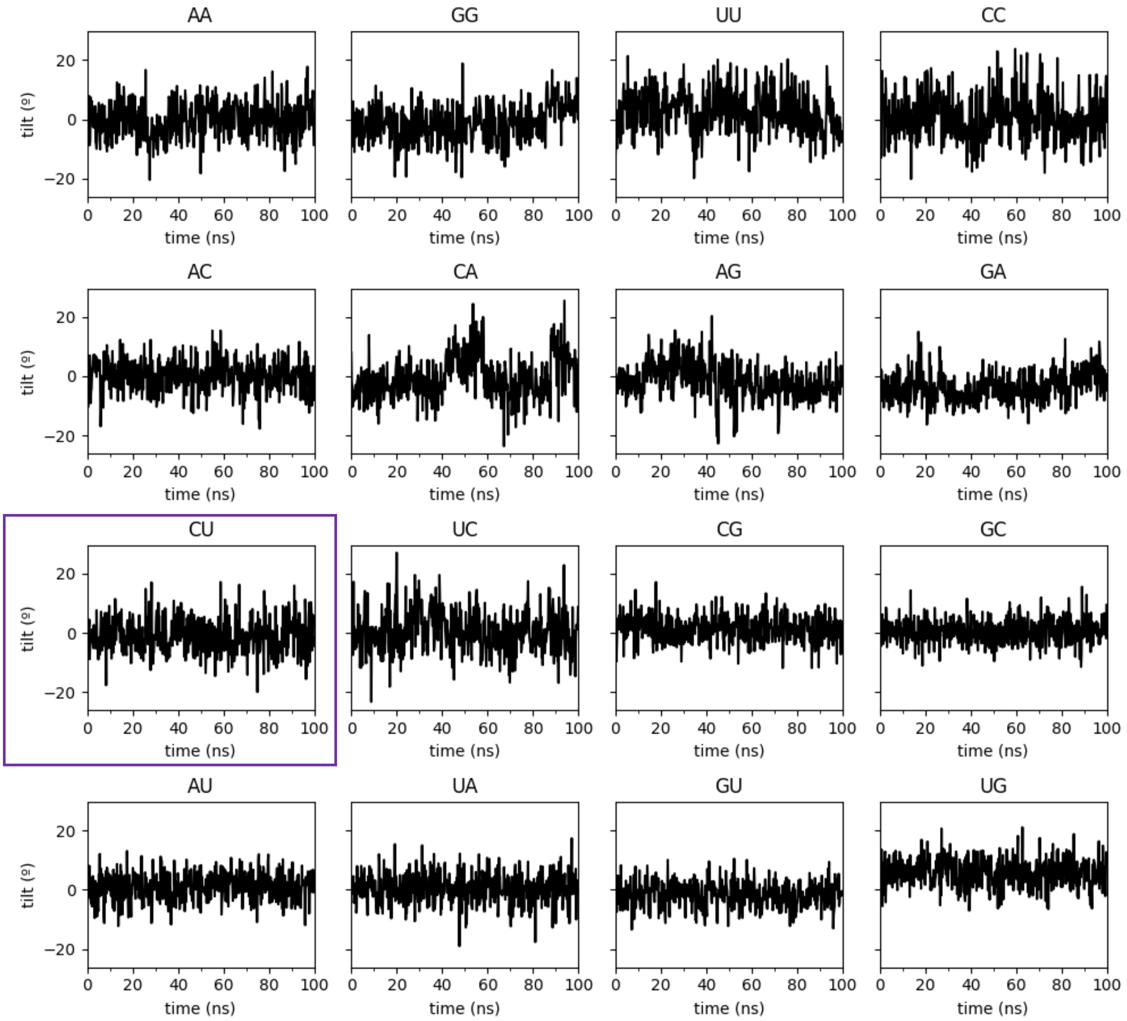

Roll parameter.

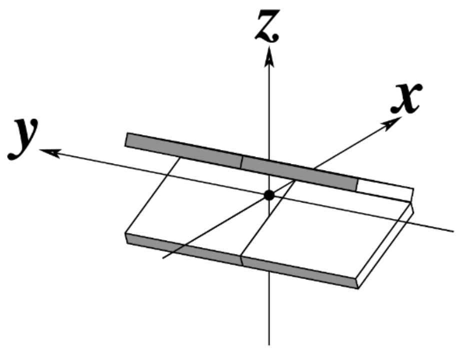

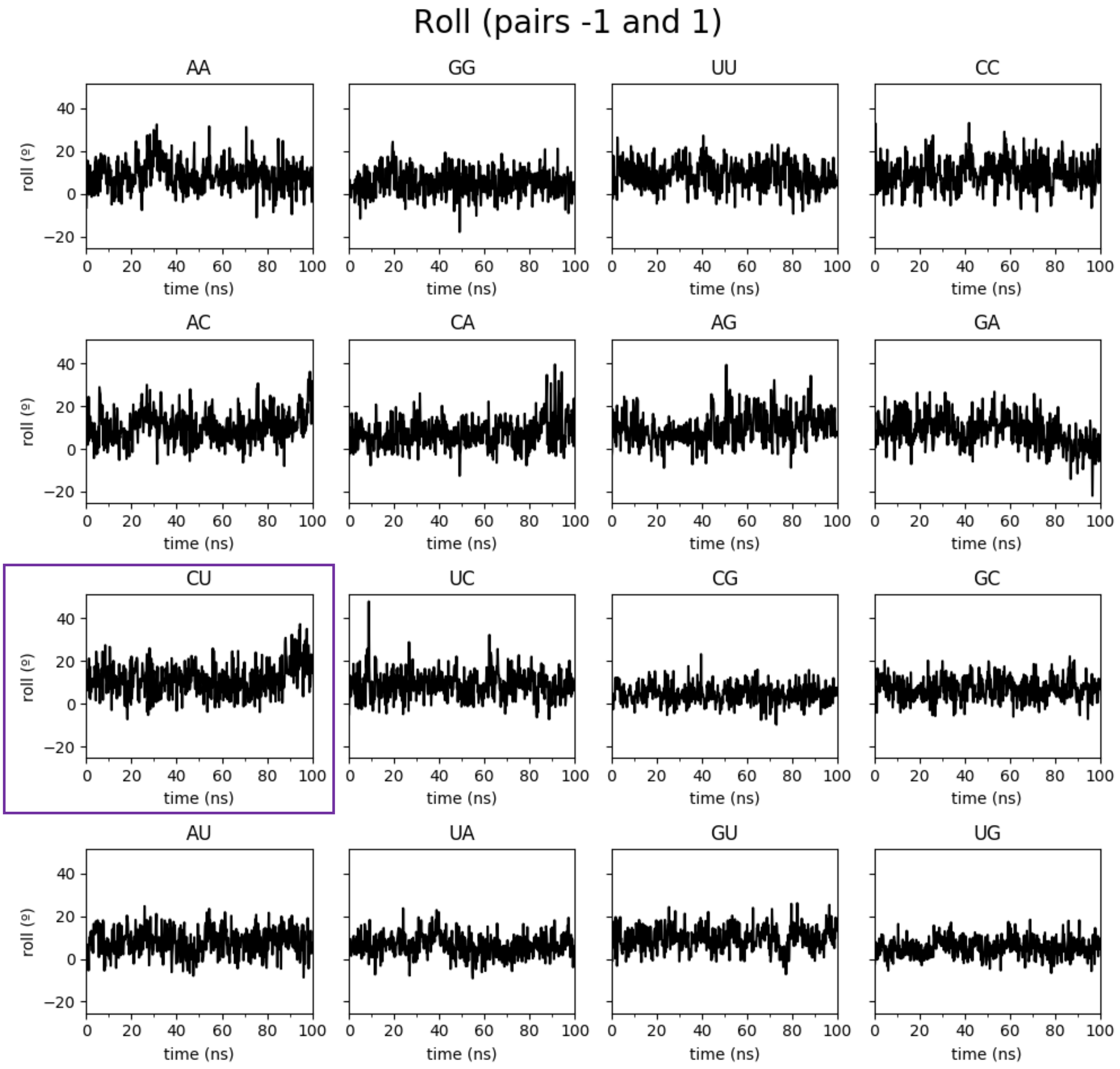

Roll (pairs 1 and 2)

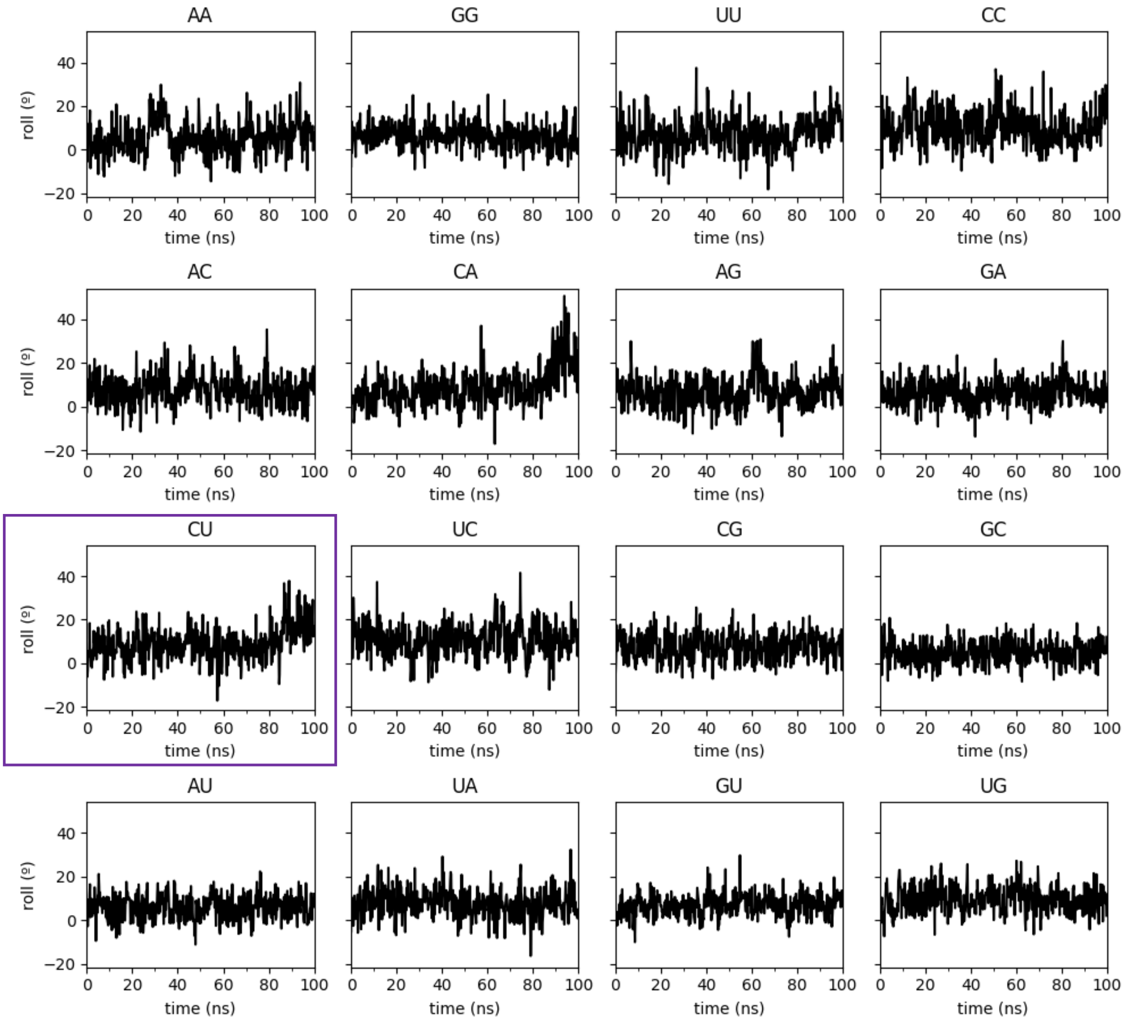

Twist parameter.

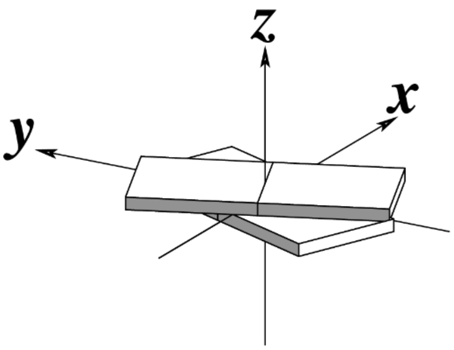

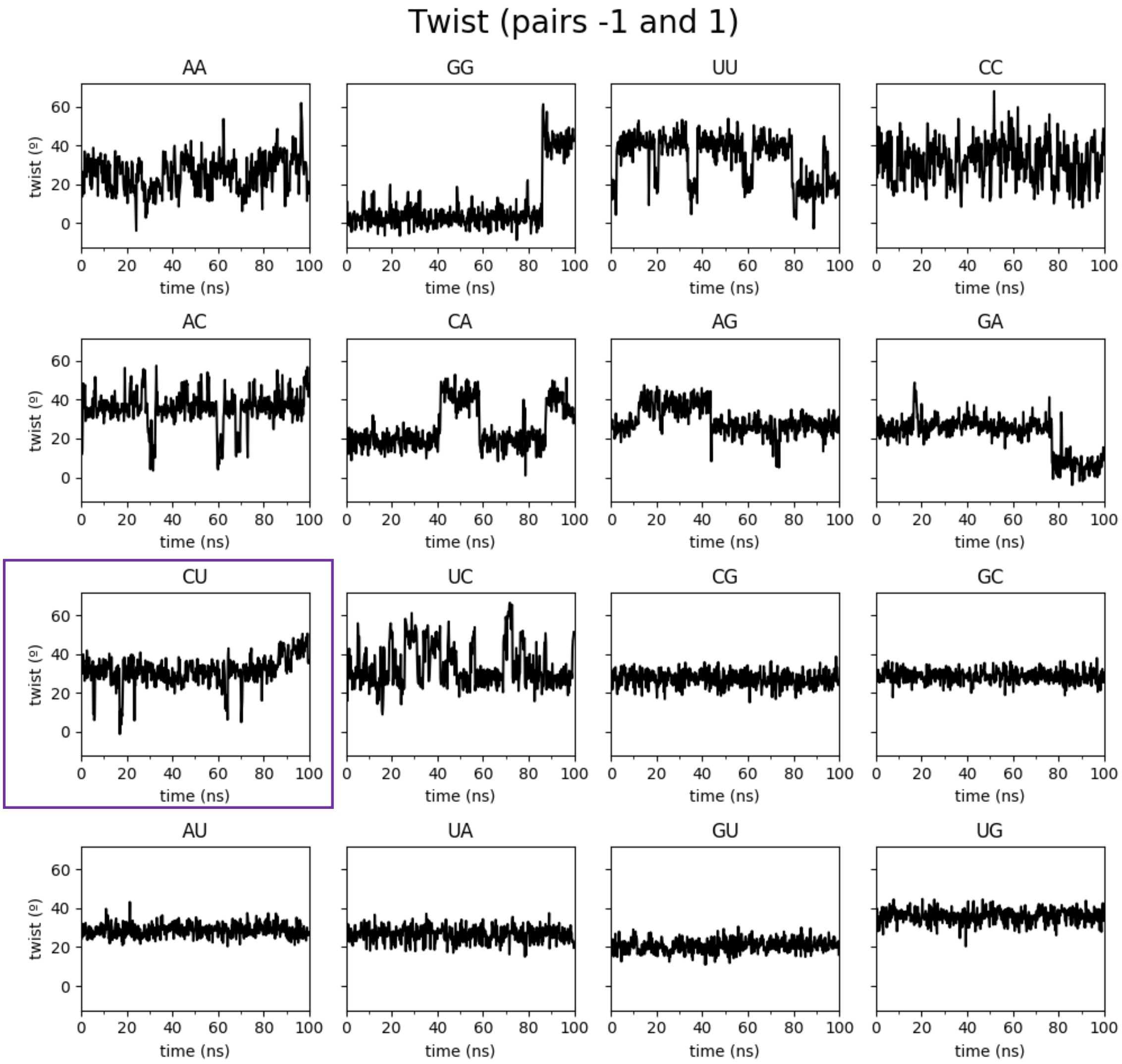

Twist (pairs 1 and 2)

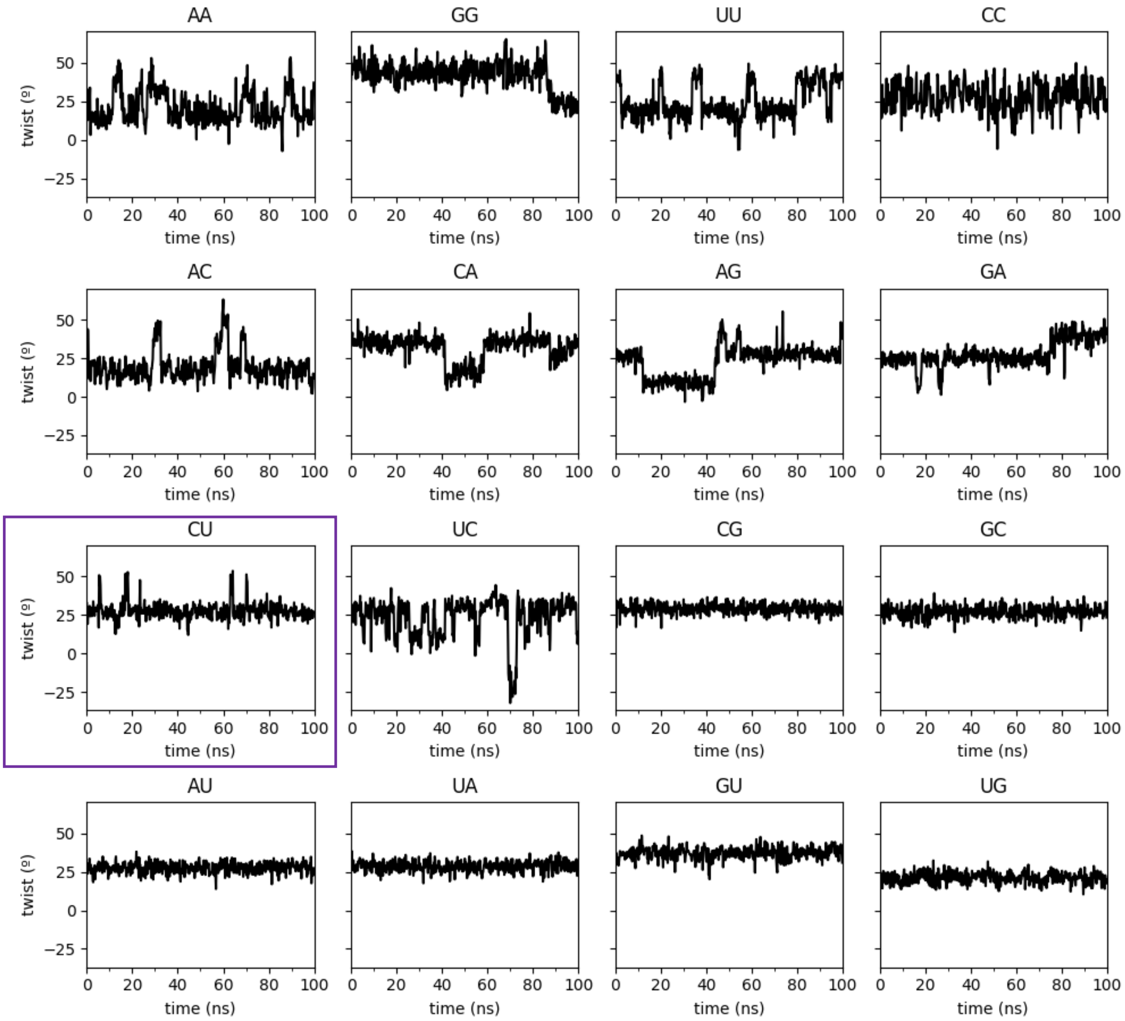

Supplement: Supplementary file 2 — Supplementary Data 1 [file 41467_2020_19129_MOESM2_ESM.pdf]
