# Supplementary material for: Identification of key sequence features required for microRNA biogenesis in plants
Source: Nat Commun. 2020 Oct 21;11:5320. doi: 10.1038/s41467-020-19129-6 (PMC7577975; doi:10.1038/s41467-020-19129-6)

Supplementary Data 2.

Molecular Dynamics simulation of *MIR164C* containing the nucleotide pairs modified at position 23.

A- Axial bend through the simulation of *MIR164C* variants. Above each graph the nucleotide pair is indicated. wt variant graph is highlighted with a purple rectangle.

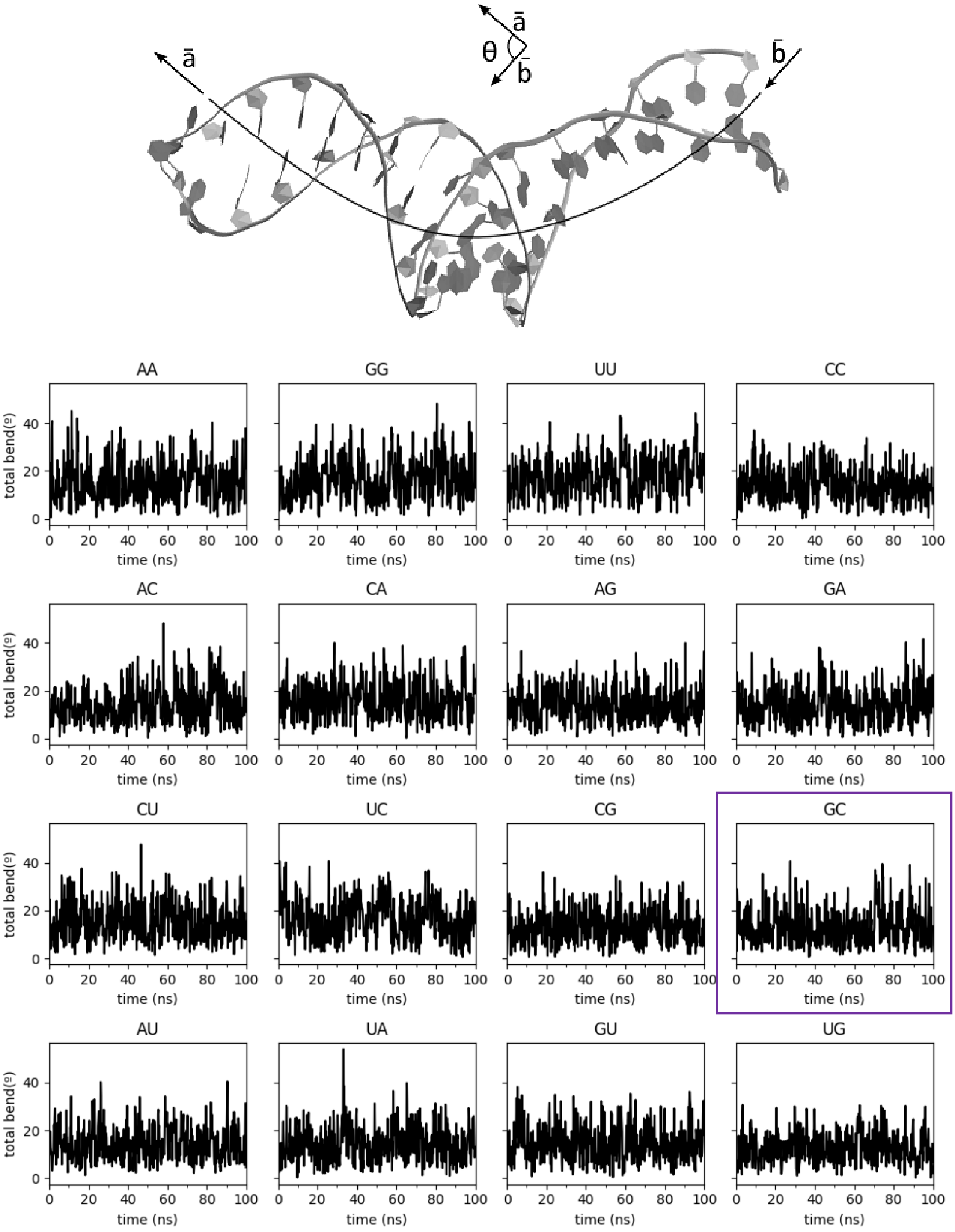

B- Structural parameters for the dsRNA calculated using Curves+.

A segment of *MIR164C* containing the nucleotide pairs modified (corresponding to positions 23) were used for these analyses.

A schematic diagram is depicted representing each parameter based on previous work (Xiang-Jun Lu, Wilma K. Olson, 3DNA: a software package for the analysis, rebuilding and visualization of three-dimensional nucleic acid structures, Nucleic Acids Research, Volume 31, Issue 17, 1 September 2003, Pages 5108–5121, https://doi.org/10.1093/nar/gkg680; Lu, X., Olson, W. 3DNA: a versatile, integrated software system for the analysis, rebuilding and visualization of three-dimensional nucleic-acid structures. Nat Protoc 3, 1213–1227 (2008). https://doi.org/10.1038/nprot.2008.104)

In these diagrams, the shaded edge (facing the viewer) denotes the minor-groove side of a base or base pair as indicated by the author (http://x3dna.org/highlights/schematic-diagrams-of-base-pair-parameters).

The parameters shown correspond to position 1 of the wt and mutated *MIR164C* precursors. The purple rectangle indicates the wt variant graph

TRASLATIONAL PARAMETERS

Shear parameter.

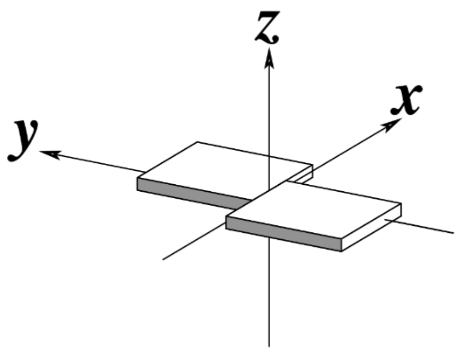

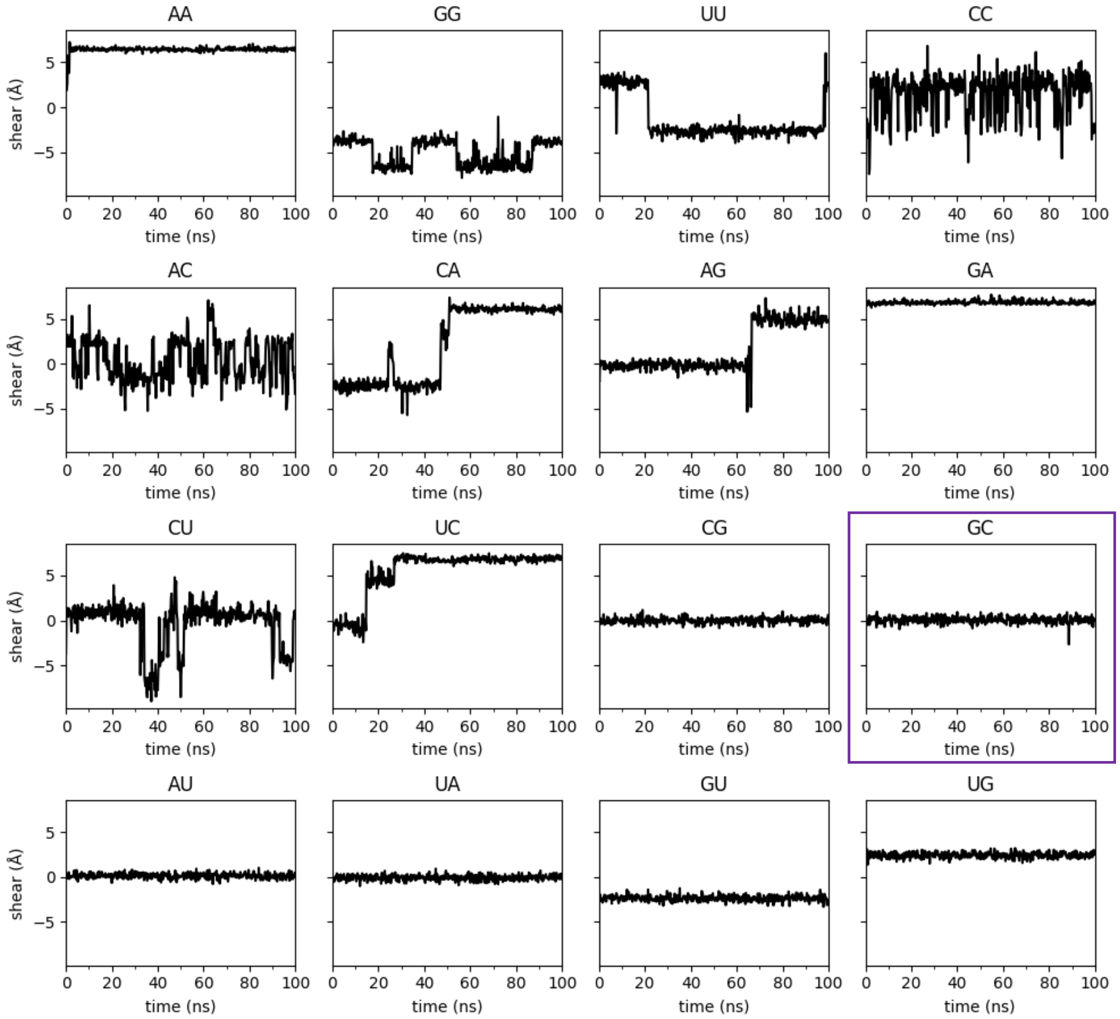

Stretch parameter.

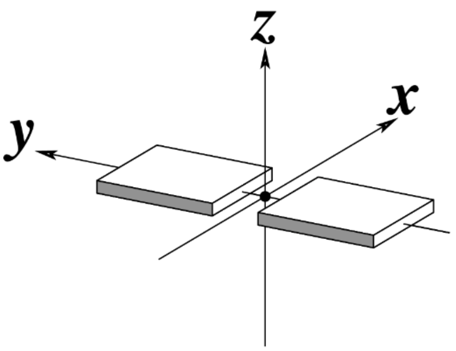

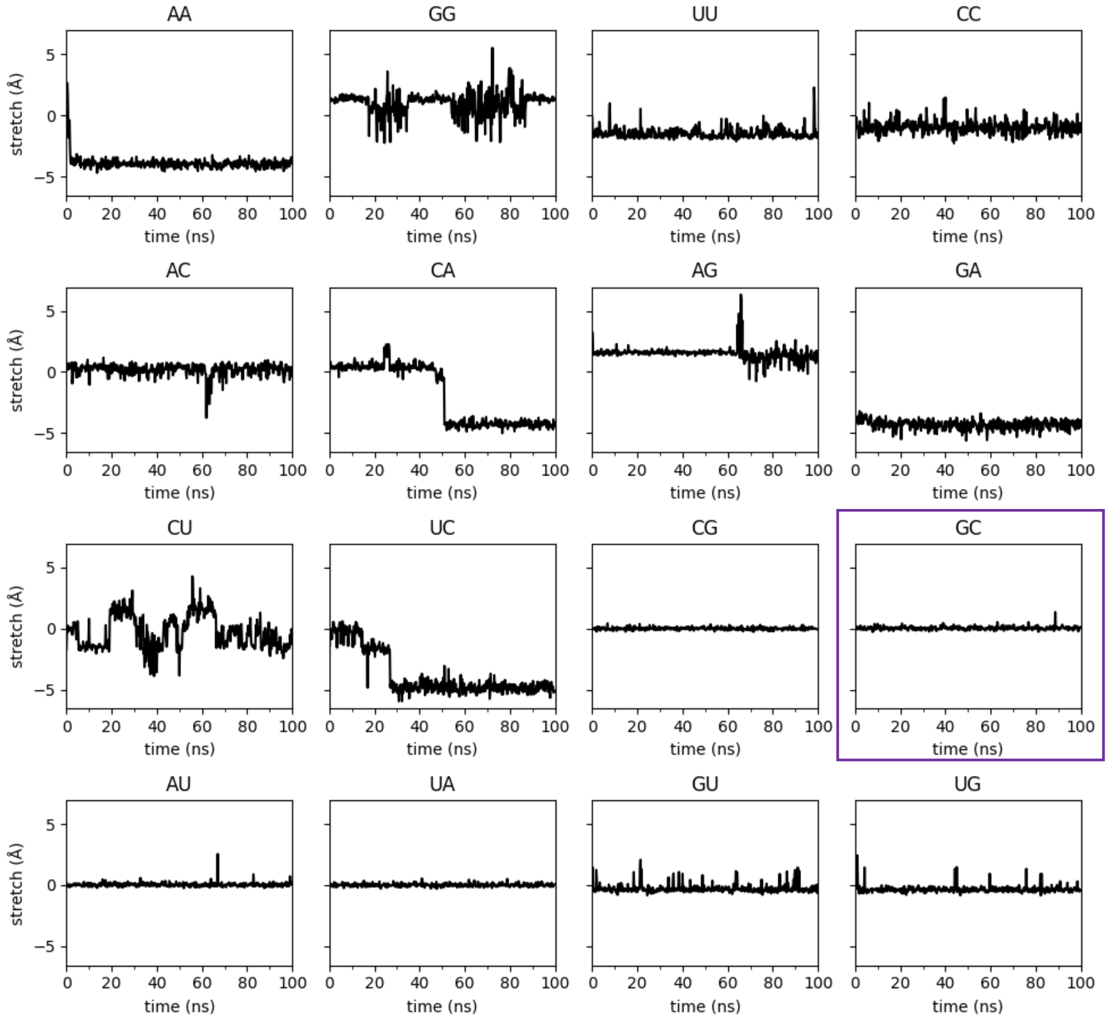

Stagger parameter.

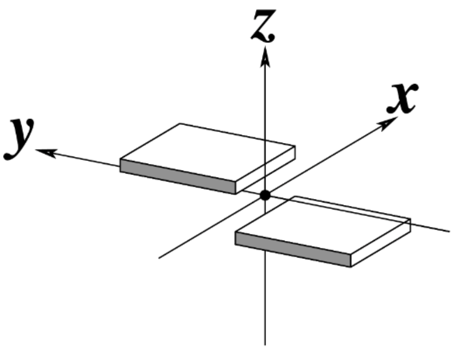

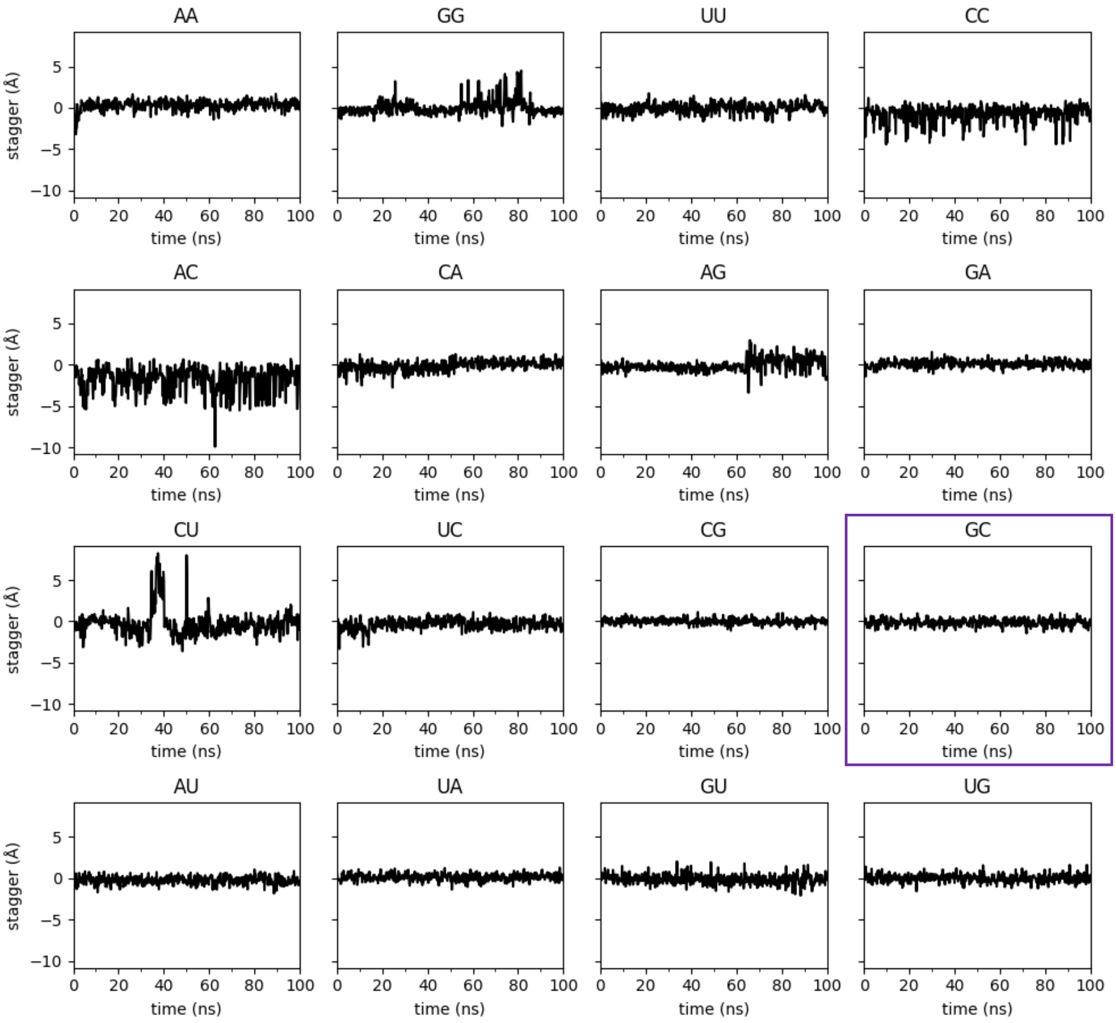

Shift parameter.

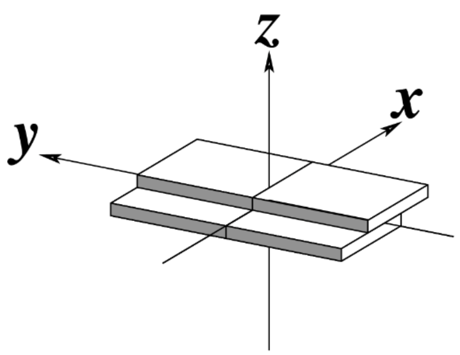

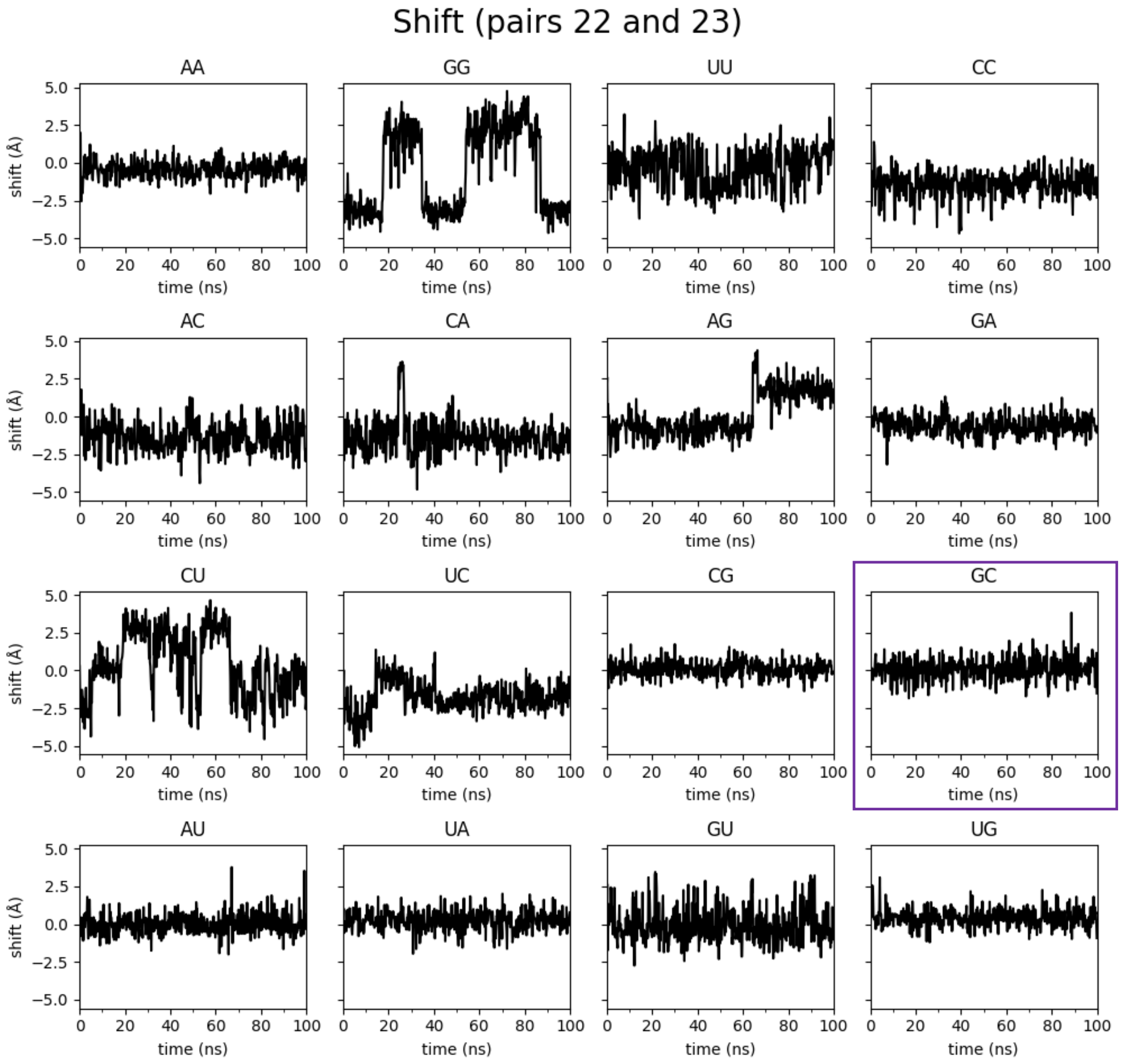

Shift (pairs 23 and 24)

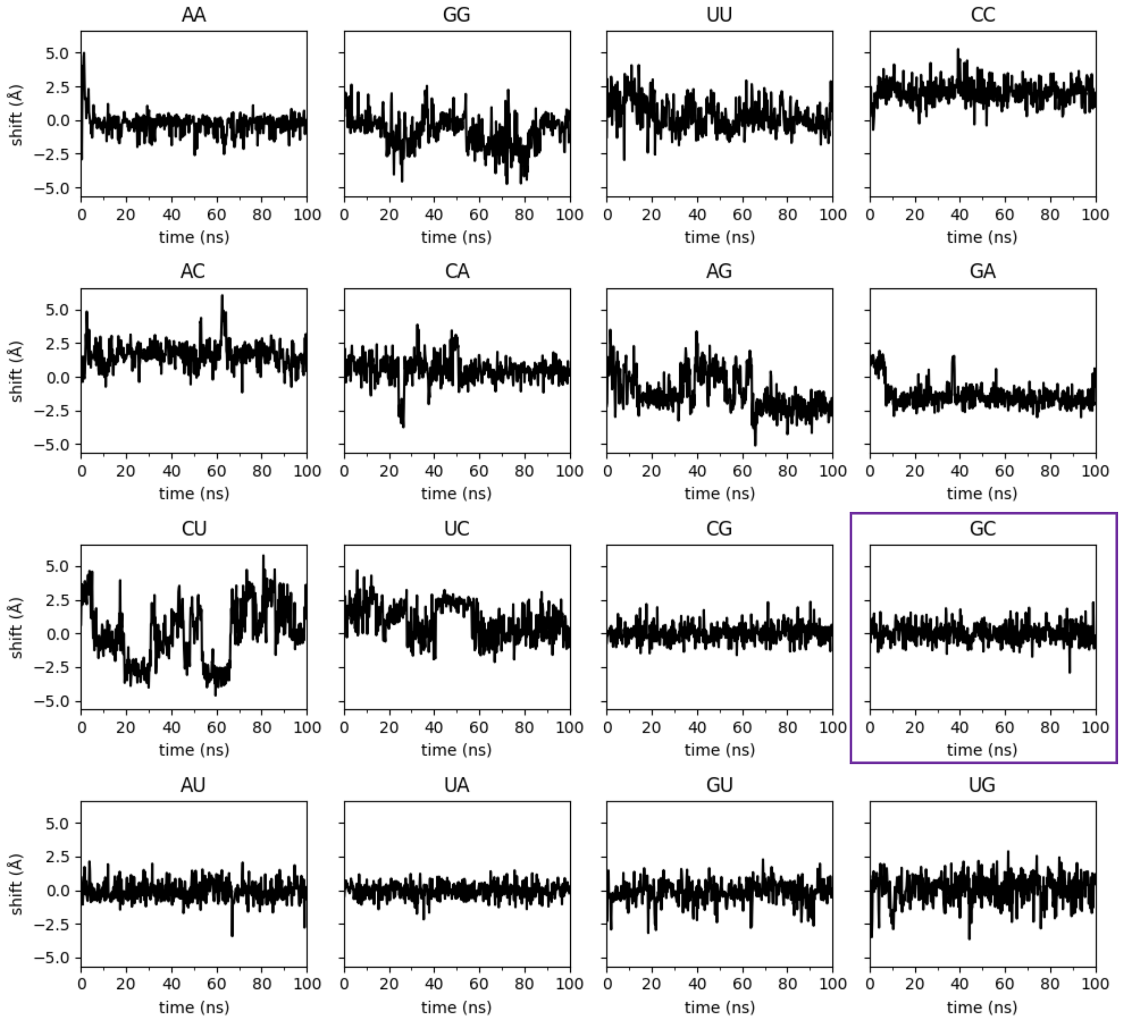

Slide parameter.

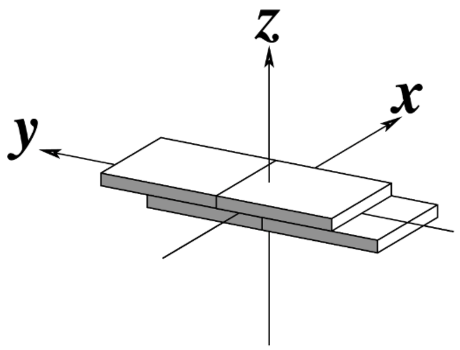

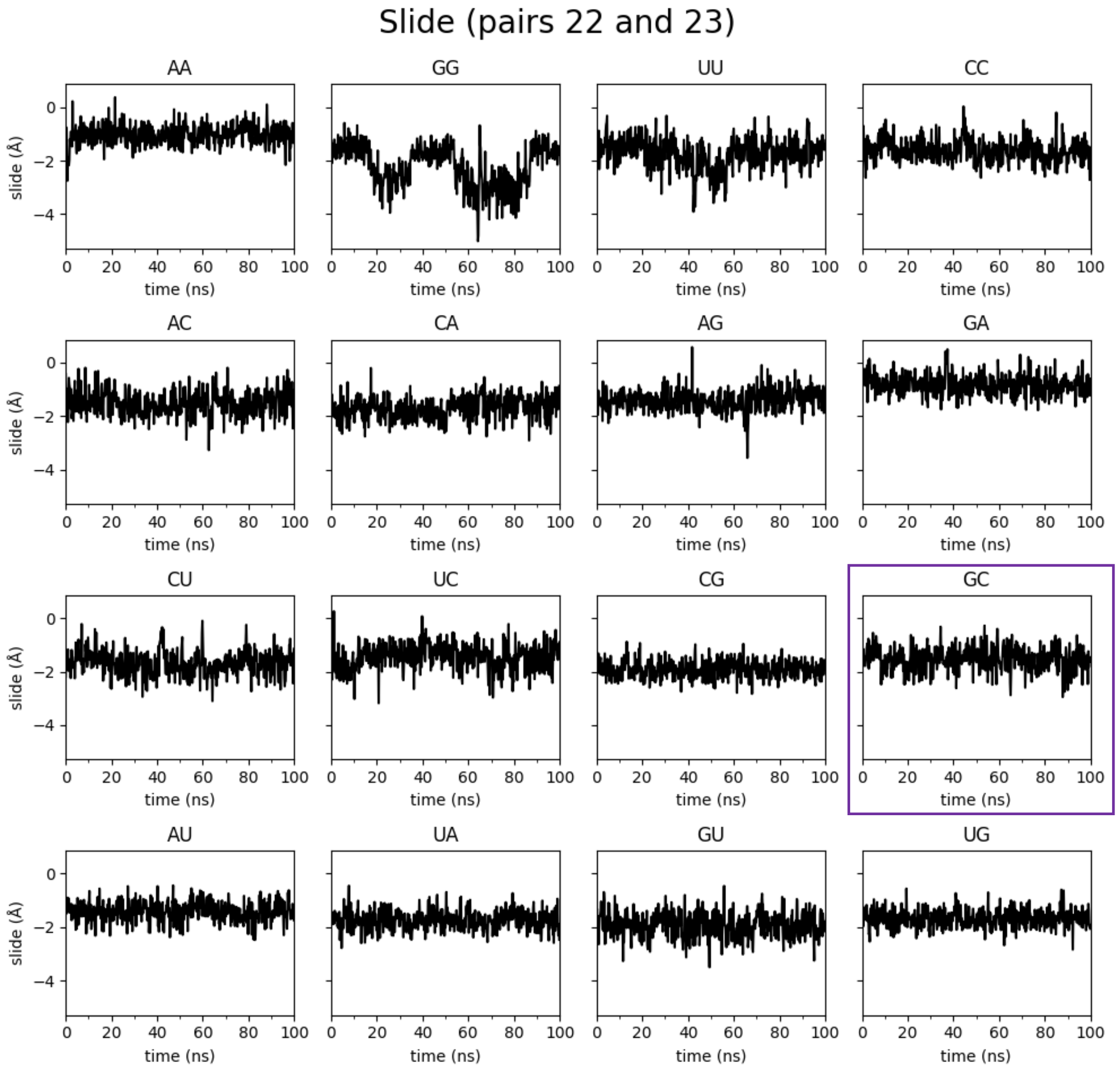

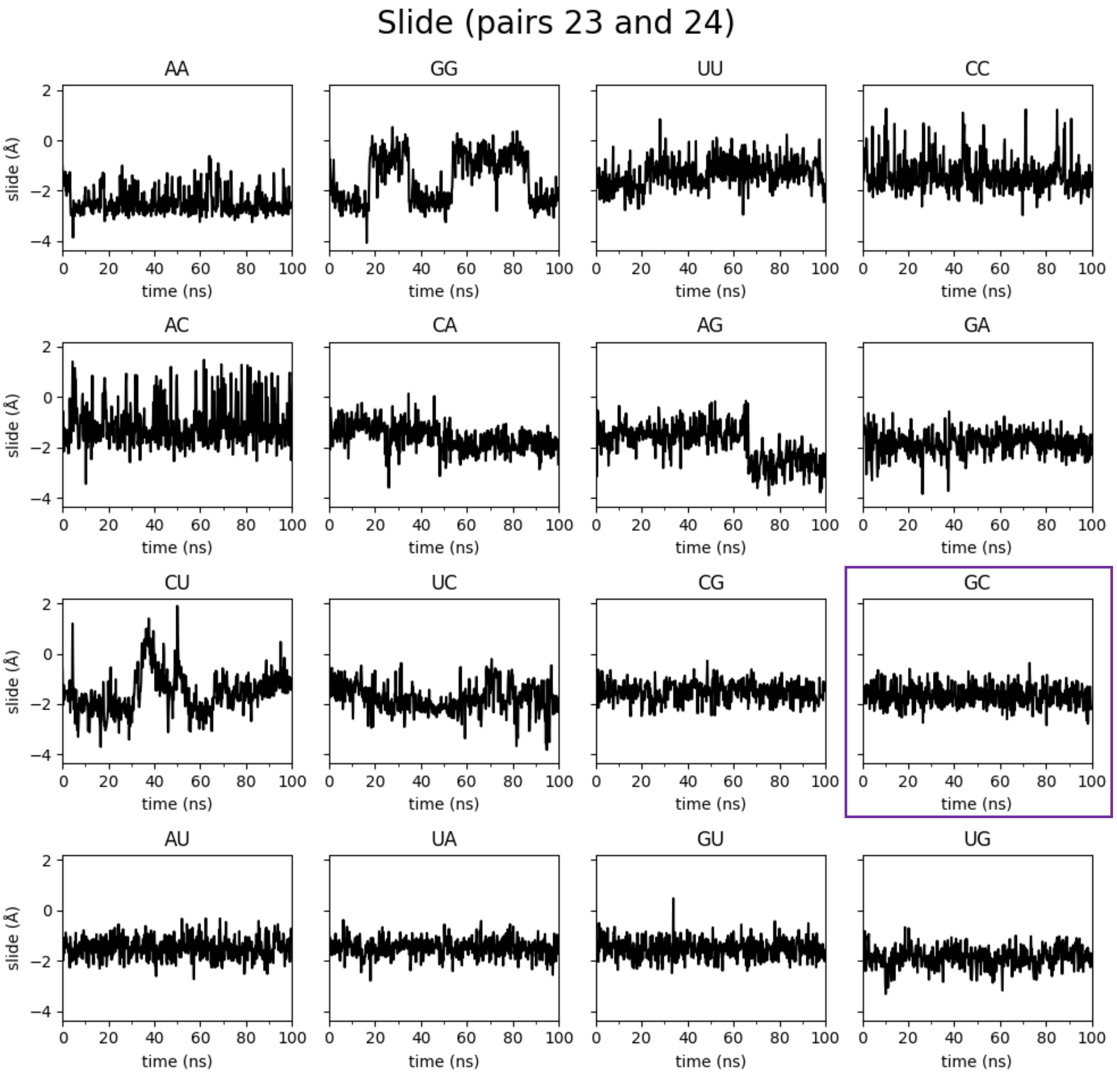

Slide (pairs 23 and 24)
AA
GG
UU
CC
AC
CA
AG
GA
CU
UC
CG
GC
AU
UA
GU
UG
slide (Å)
time (ns)
2
0
−2
−4
0
20
40
60
80
100

Rise parameter.

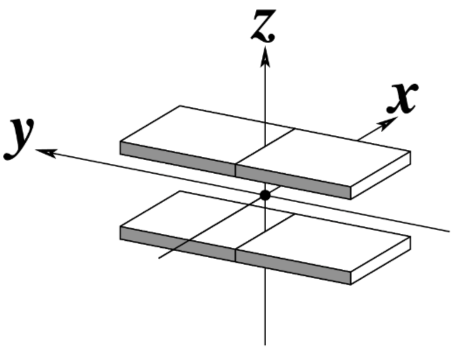

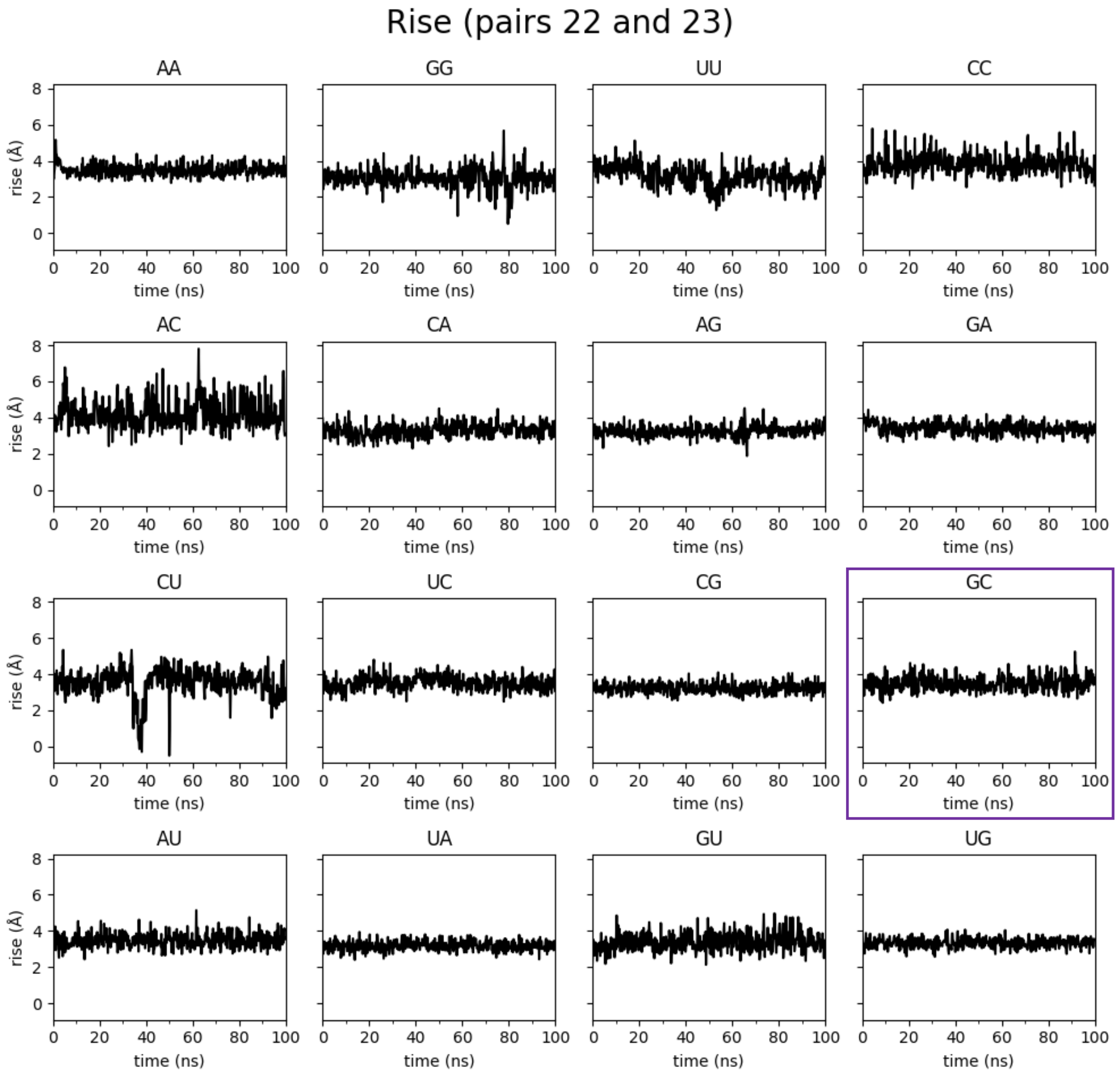

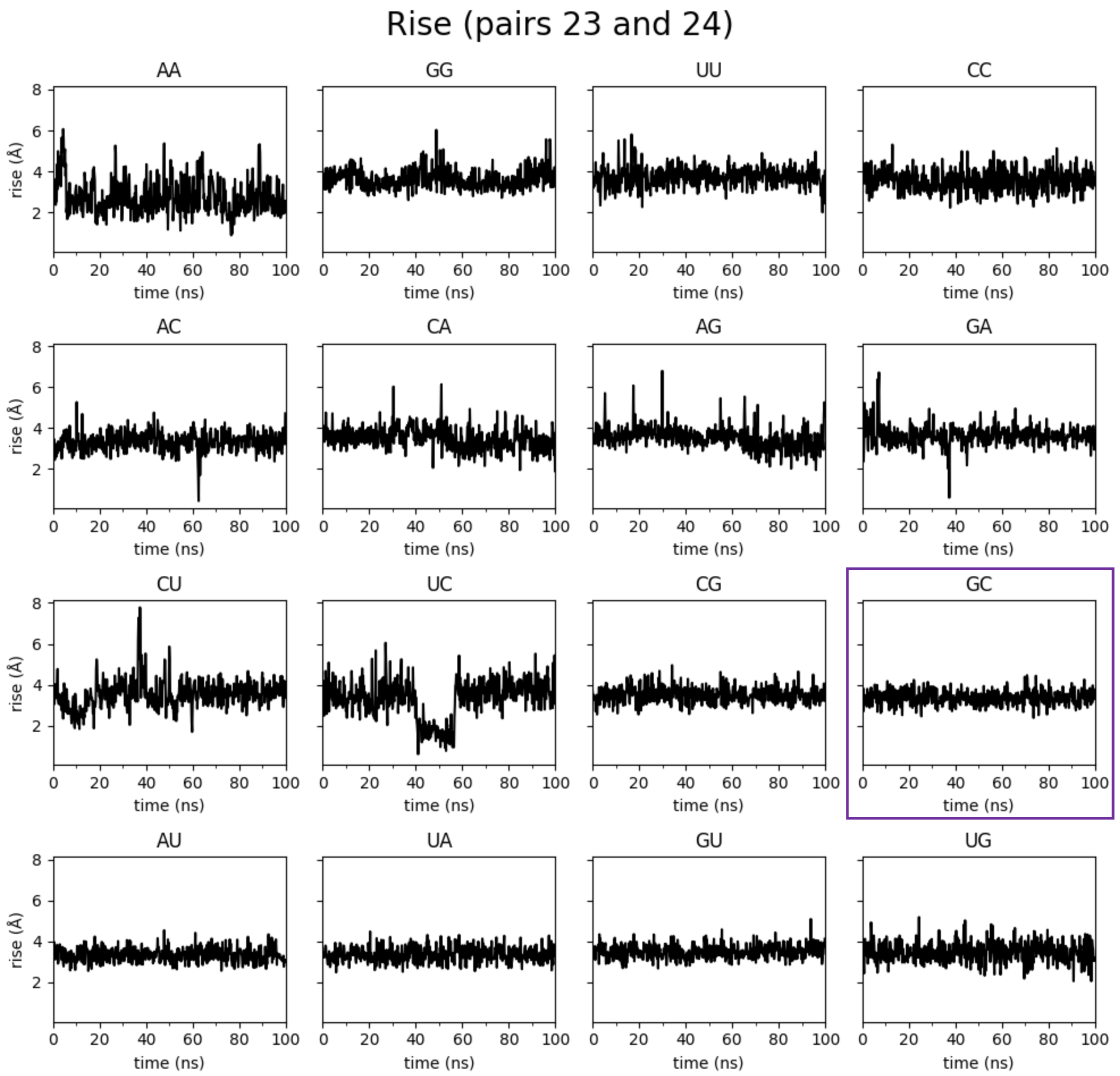
Rise (pairs 23 and 24)
AA
GG
UU
CC
AC
CA
AG
GA
CU
UC
CG
GC
AU
UA
GU
UG
rise (Å)
time (ns)
0
20
40
60
80
100
2
4
6
8

ROTATIONAL PARAMETERS

Buckle parameter.

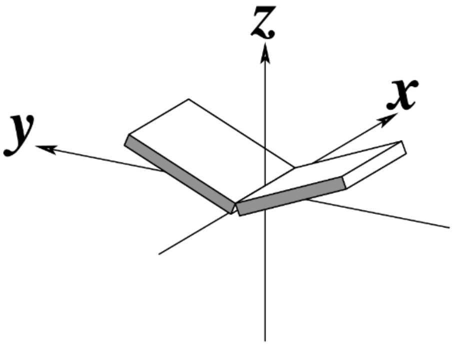

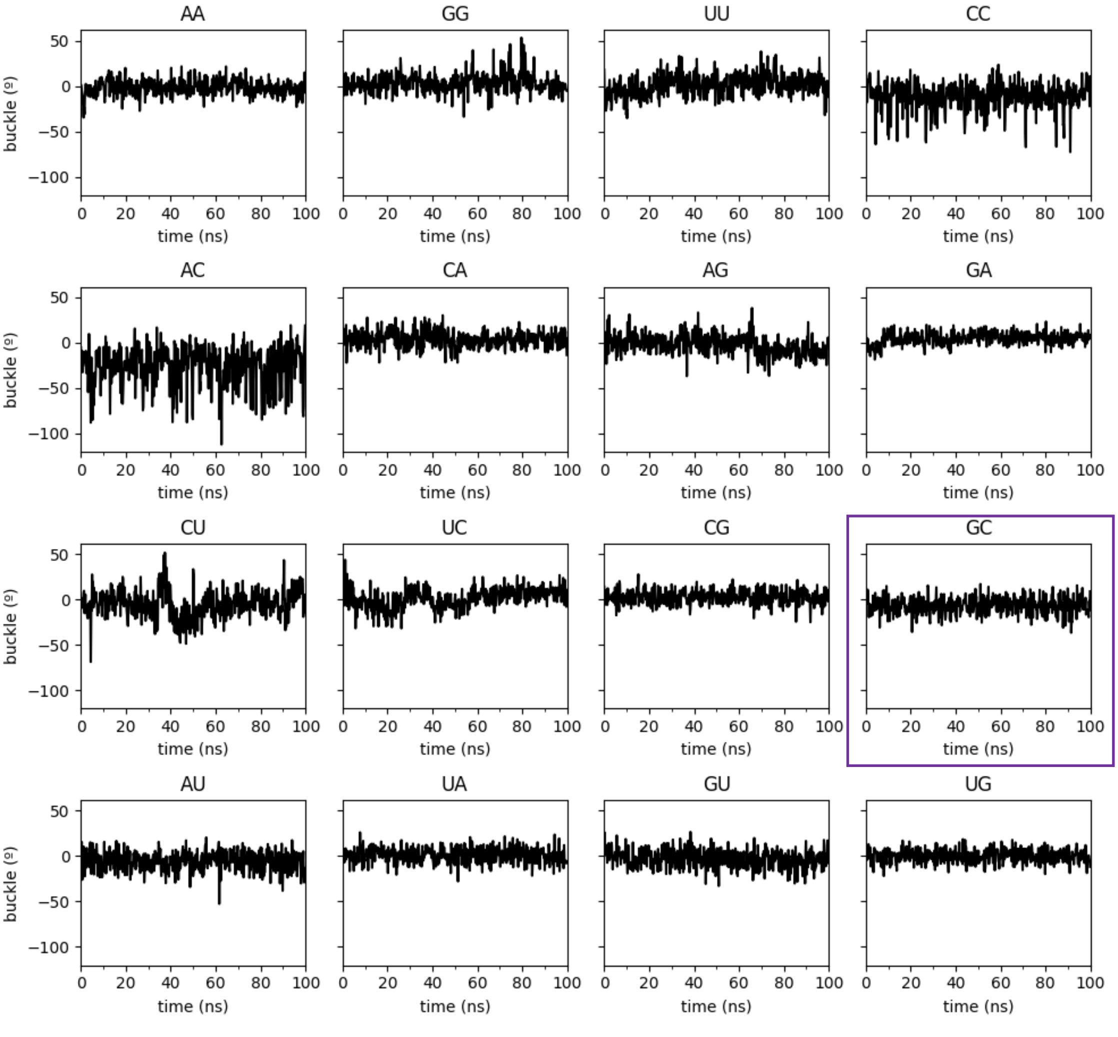

Propeller parameter.

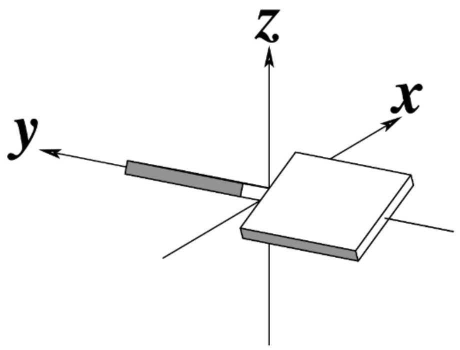

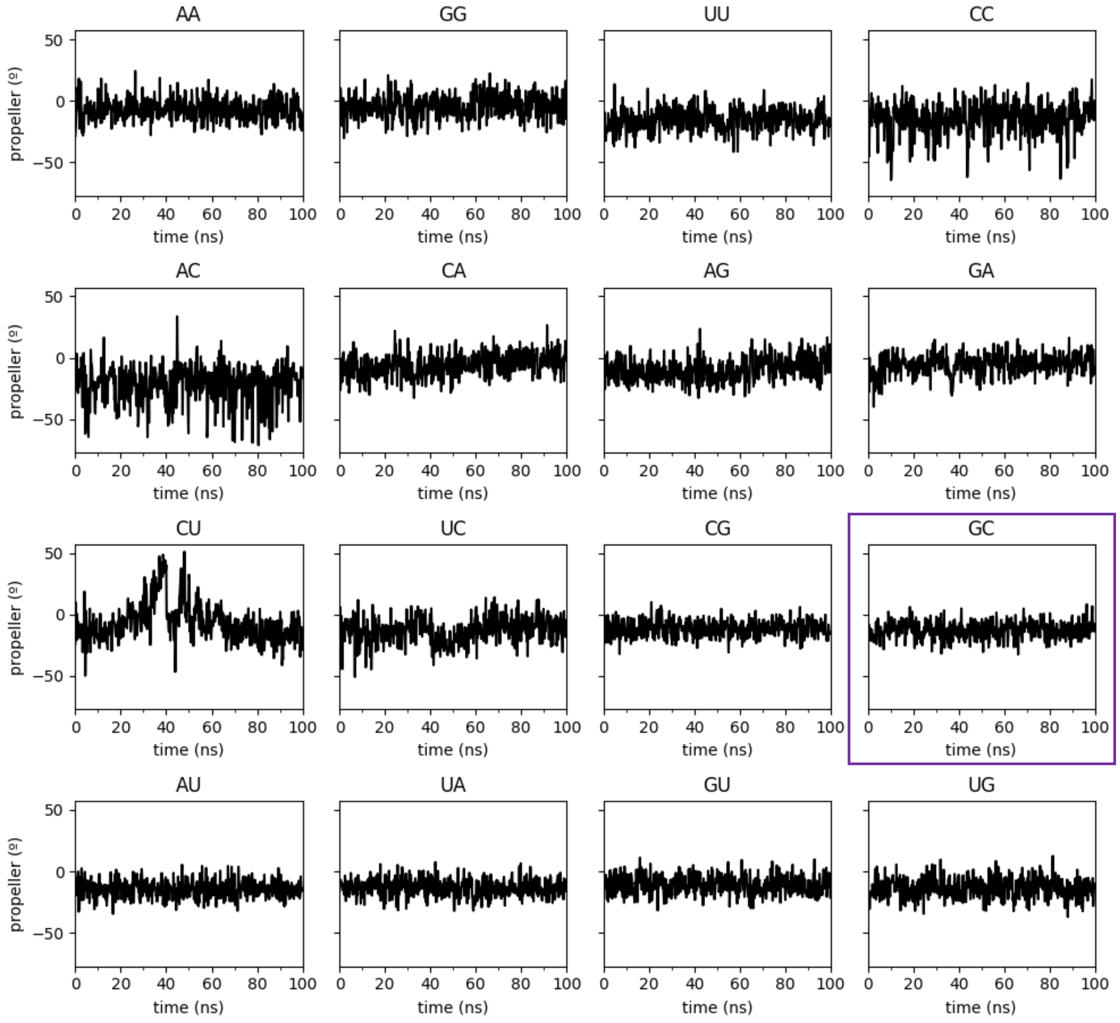

Opening parameter.

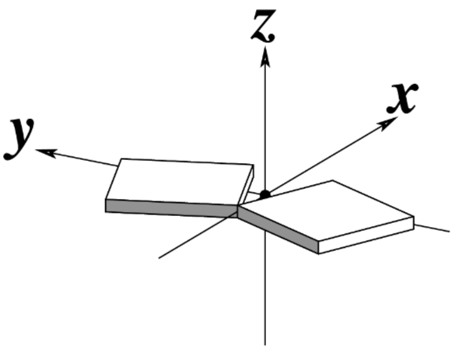

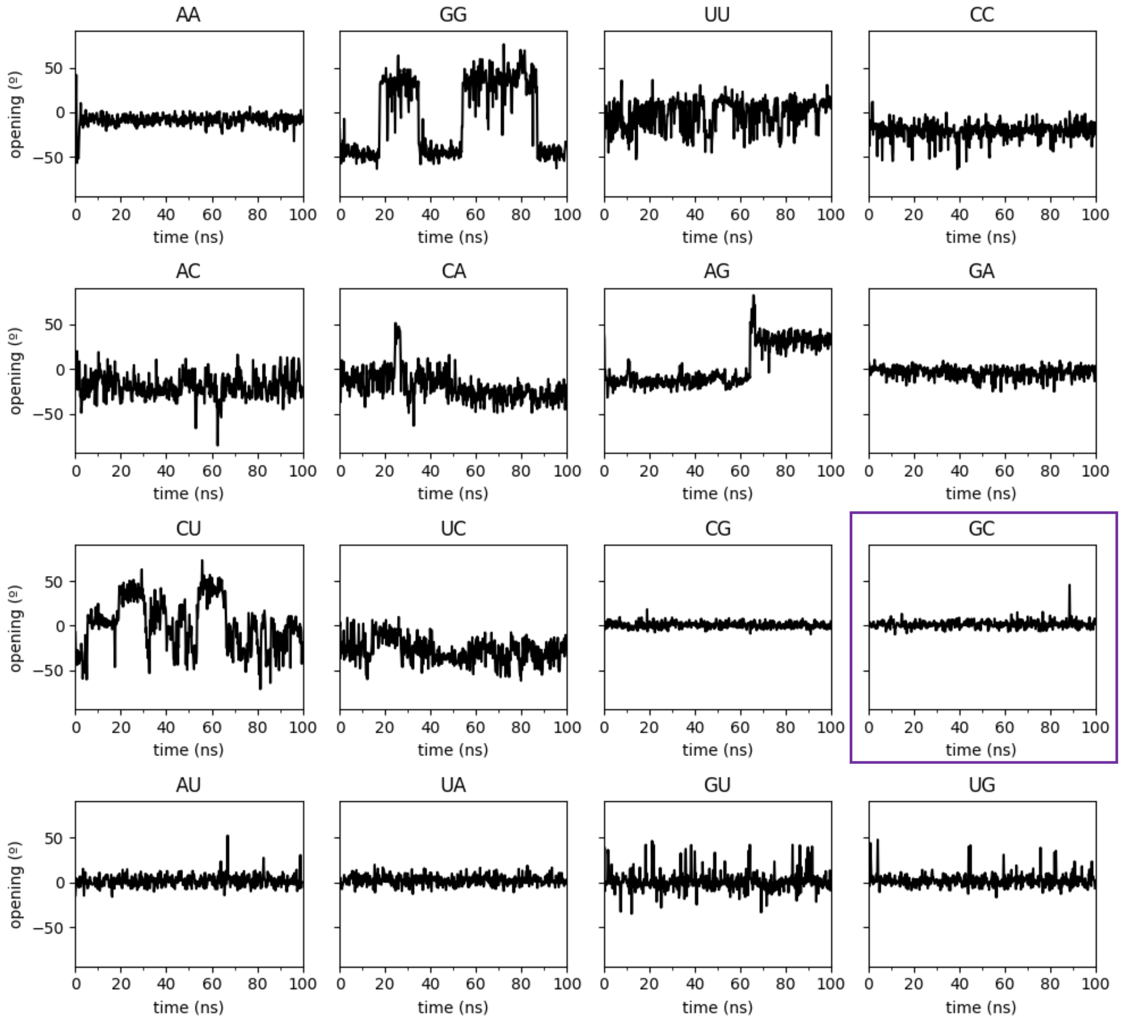

Tilt parameter.

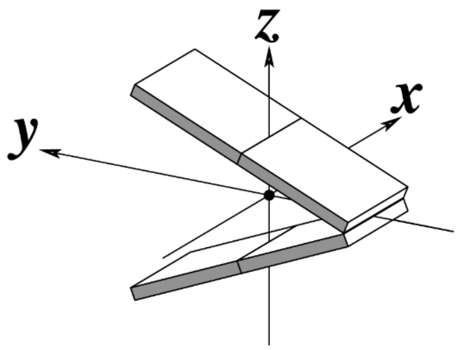

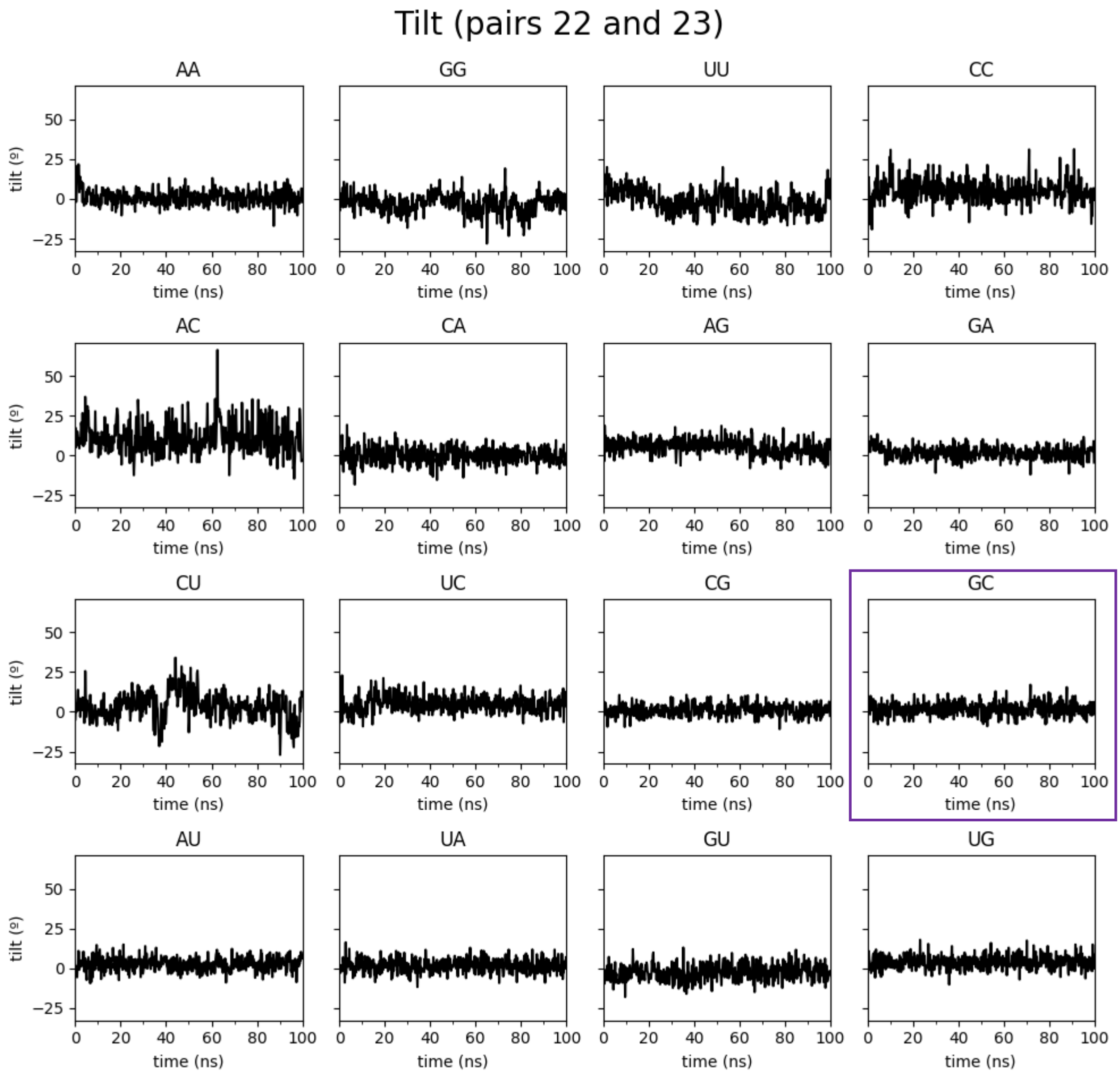

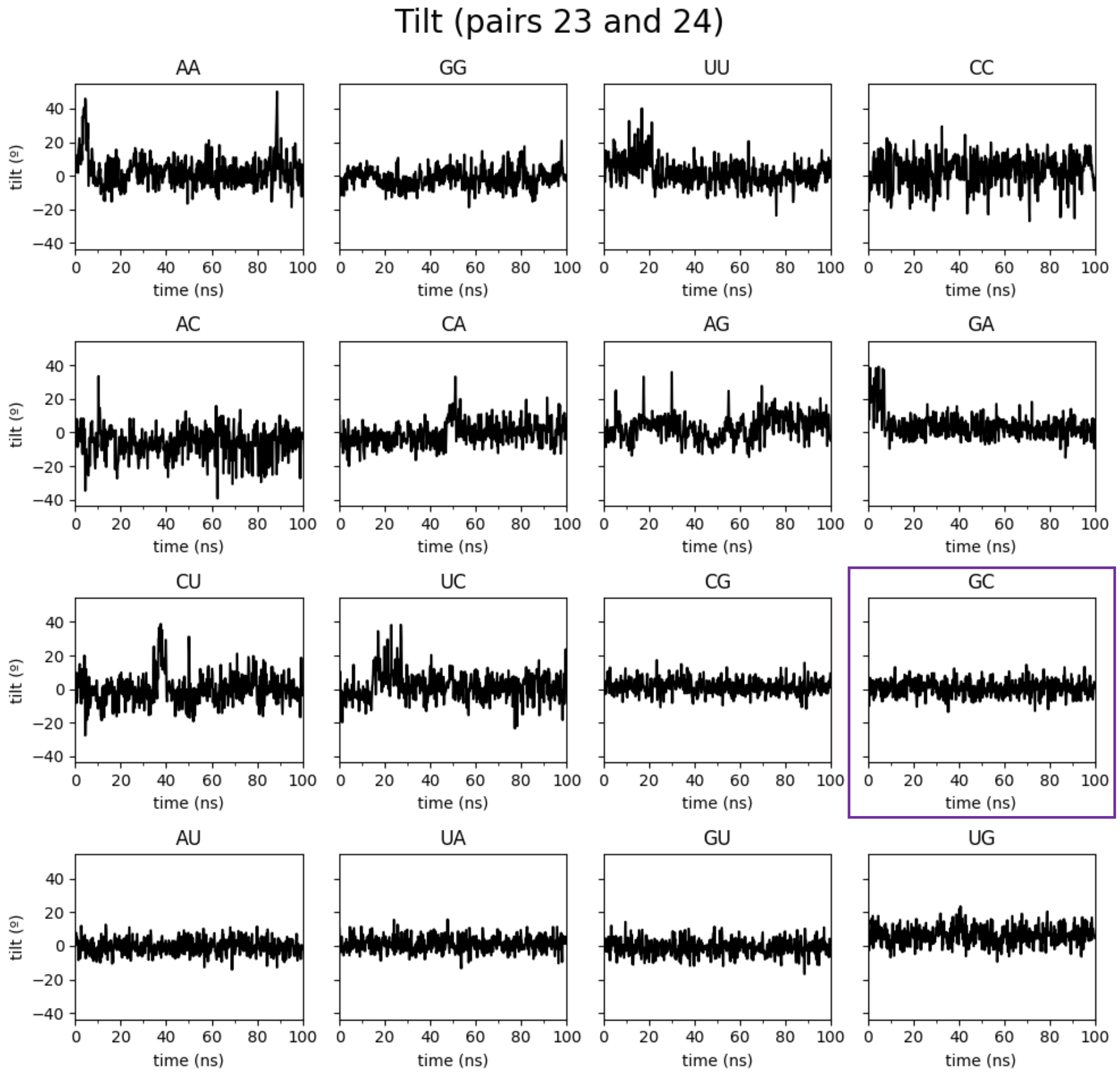
Tilt (pairs 23 and 24)
AA
GG
UU
CC
AC
CA
AG
GA
CU
UC
CG
GC
AU
UA
GU
UG
tilt (º)
time (ns)

Roll parameter.

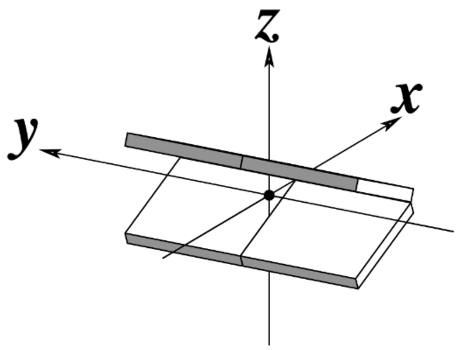

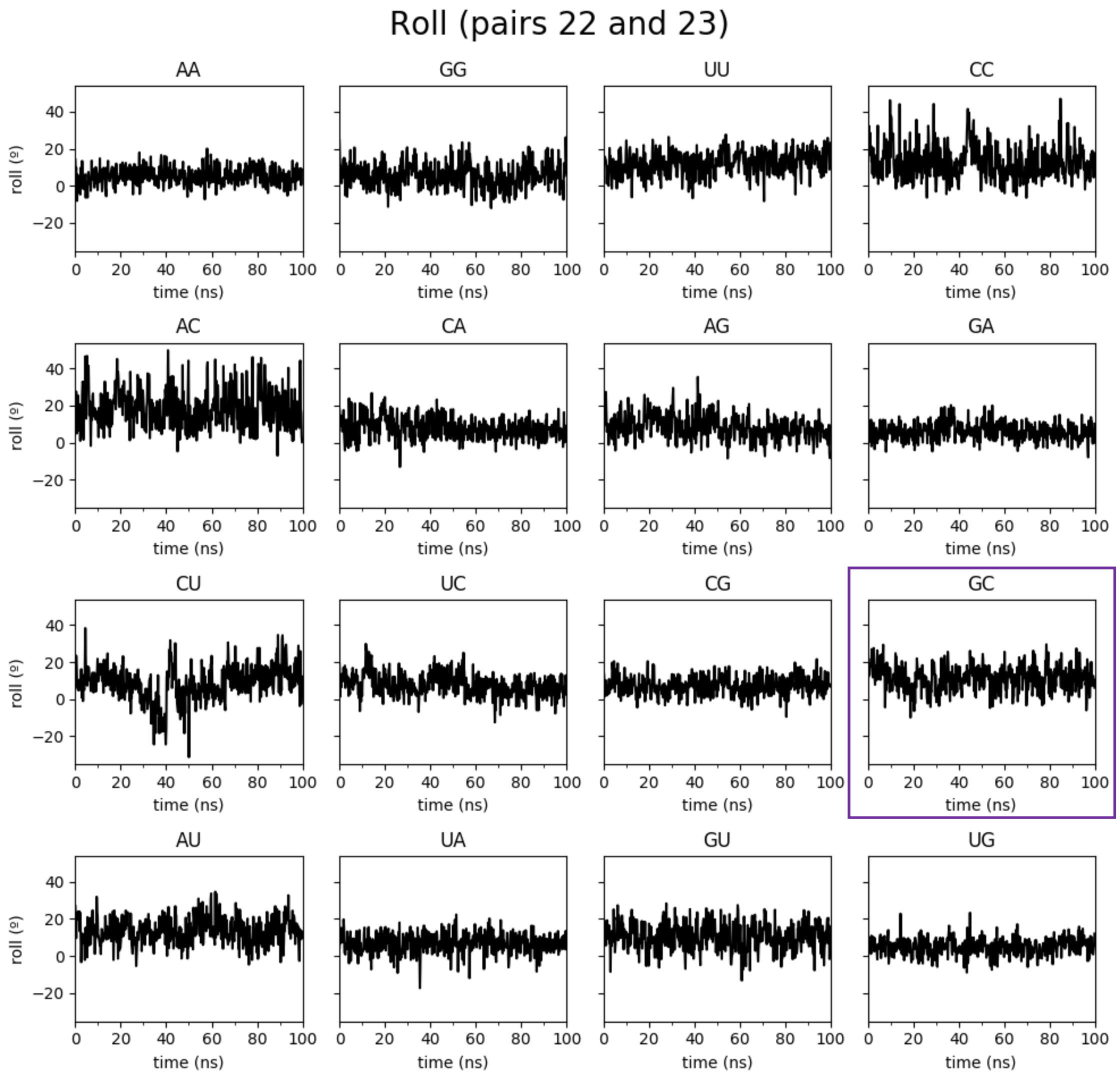

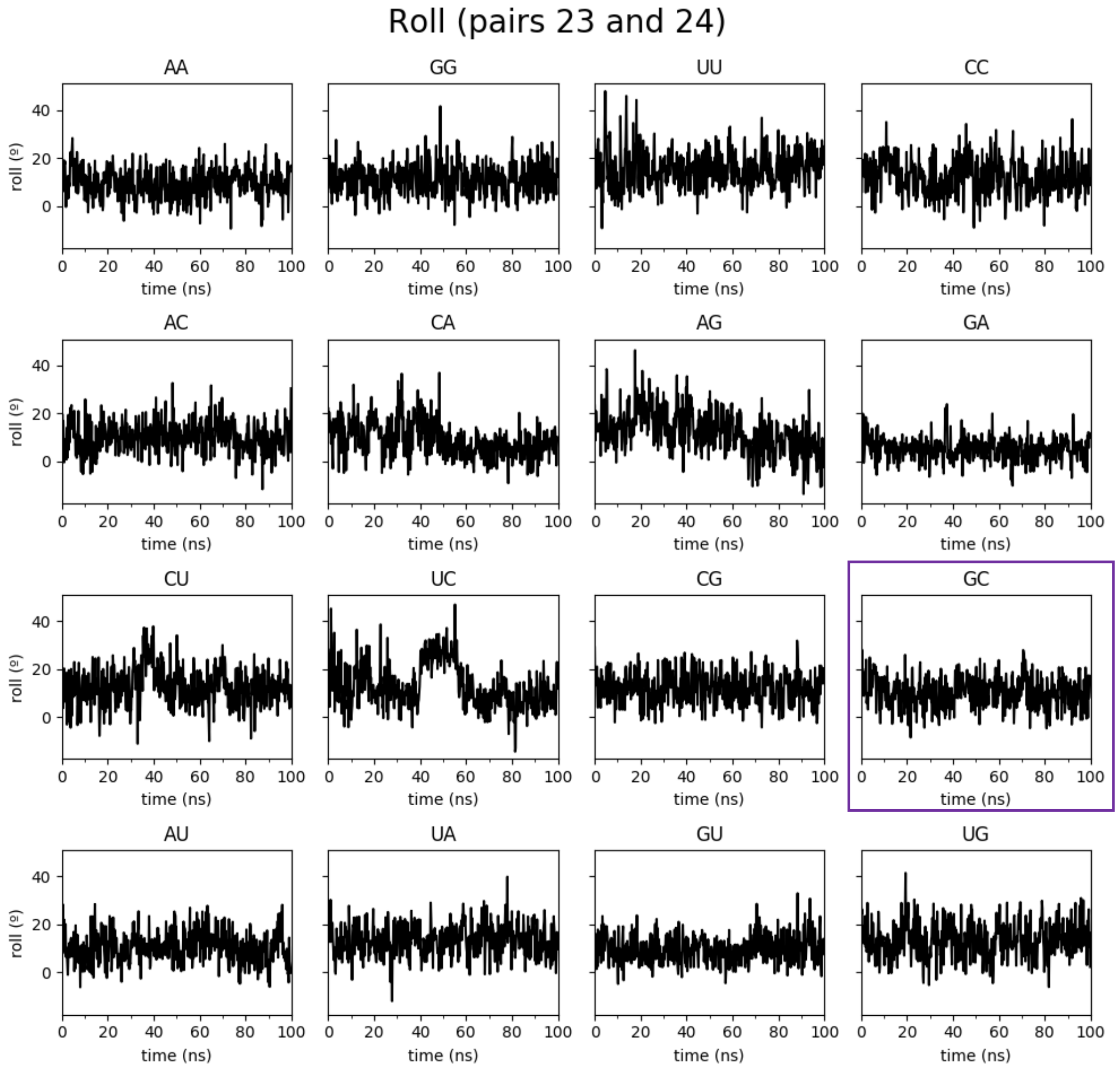
Roll (pairs 23 and 24)
AA
GG
UU
CC
AC
CA
AG
GA
CU
UC
CG
GC
AU
UA
GU
UG
roll (º)
time (ns)
0
20
40
60
80
100

Twist parameter.

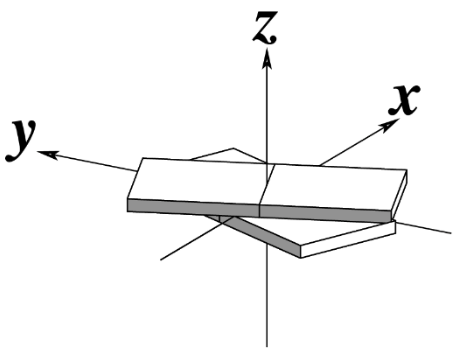

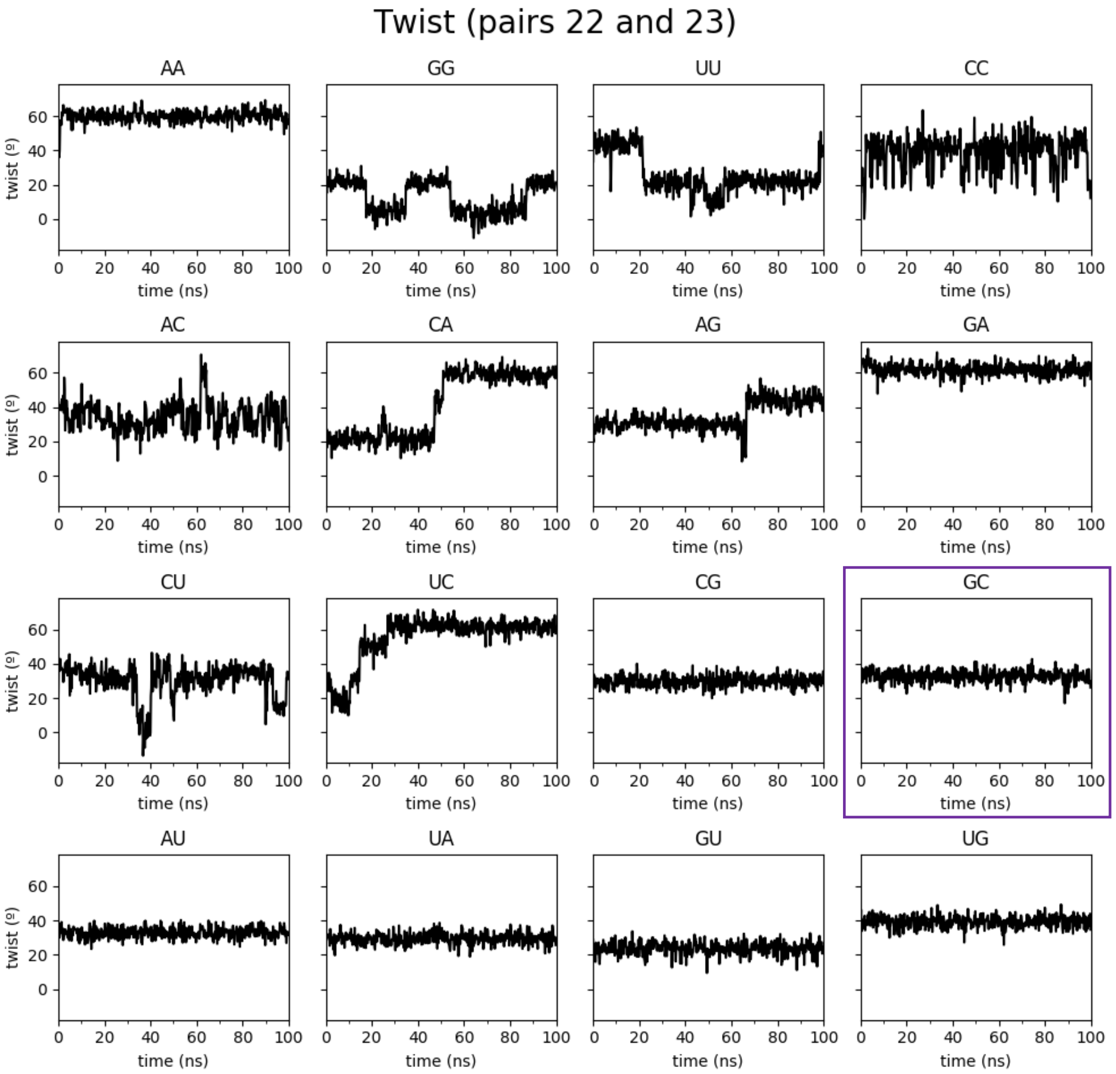

Supplement: Supplementary file 3 — Supplementary Data 2 [file 41467_2020_19129_MOESM3_ESM.pdf]
