# Supplementary material for: Identification of key sequence features required for microRNA biogenesis in plants
Source: Nat Commun. 2020 Oct 21;11:5320. doi: 10.1038/s41467-020-19129-6 (PMC7577975; doi:10.1038/s41467-020-19129-6)

Supplementary Data 3.

Conformations visited for each pair during the simulations.

A snapshot of each conformation is shown, indicating, the hydrogen bonds when they were formed between bases. A table with the hydrogen bonds information is included. In each graph, the wt variant is highlighted with a purple rectangle.

Snapshots showing the base pairs conformations visited by each variant during Molecular Dynamics simulations. On the left, numbers correspond to the conformations as indicated on Clustering graphs. Letters below each nucleotide indicates base identity: C, Cytosine; G, Guanine; A, Adenine; U, Uracil. Dashed lines indicate Hydrogen bonds formed between bases, and letters used to name them correspond to the ones and are used in the following Table.

CG and GC pairs:

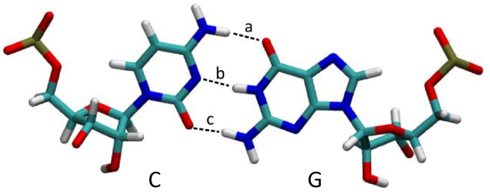

AU and UA pairs:

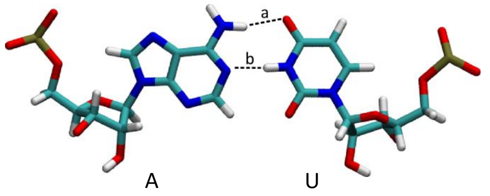

GU and UG pairs:

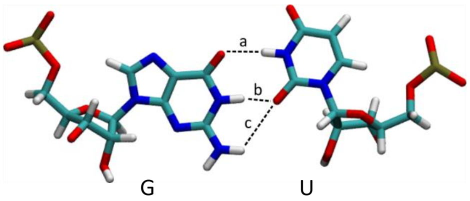

CU and UC pairs:

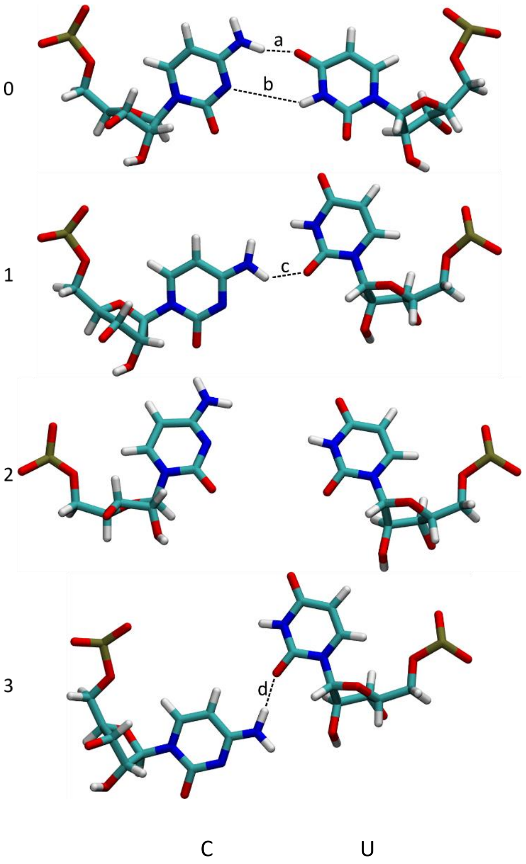

GA and AG pairs:

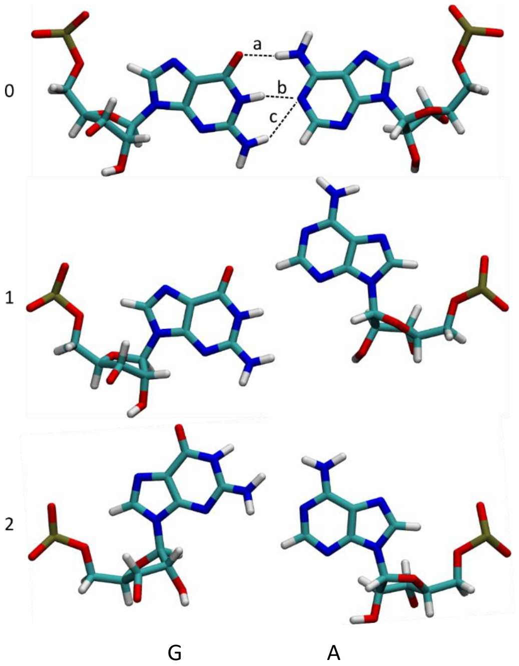

CA and AC pairs:

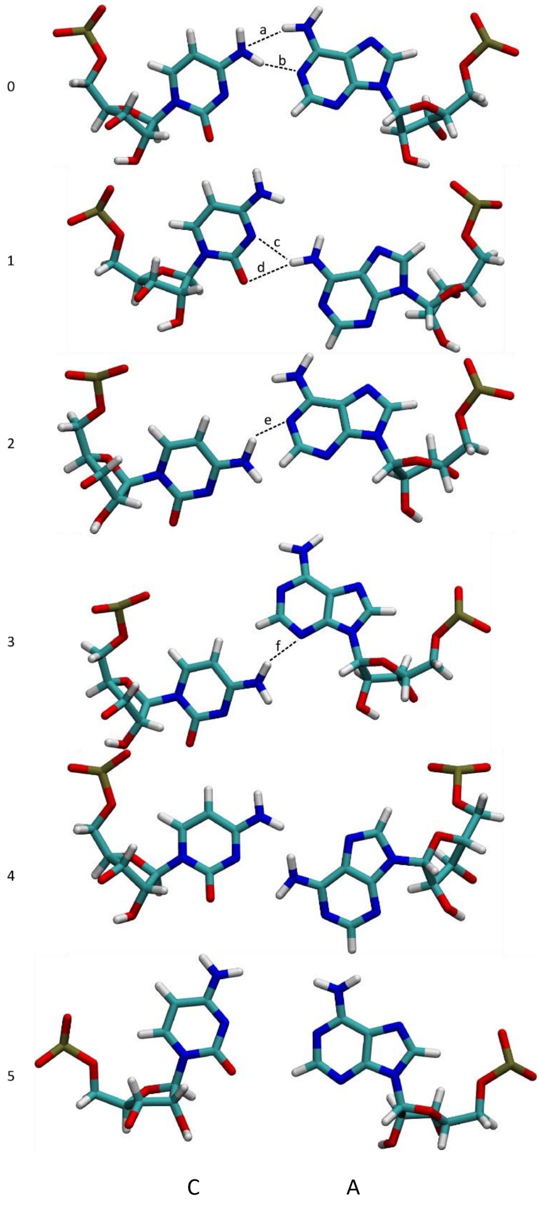

AA pair:

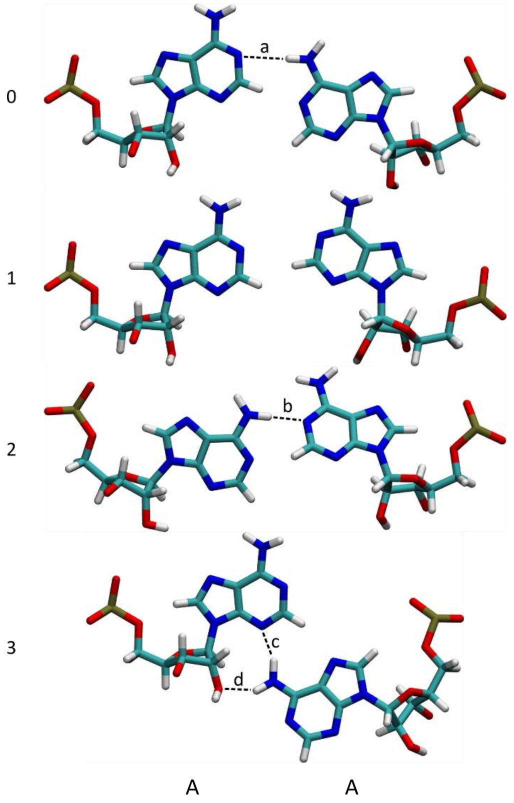

UU pair:

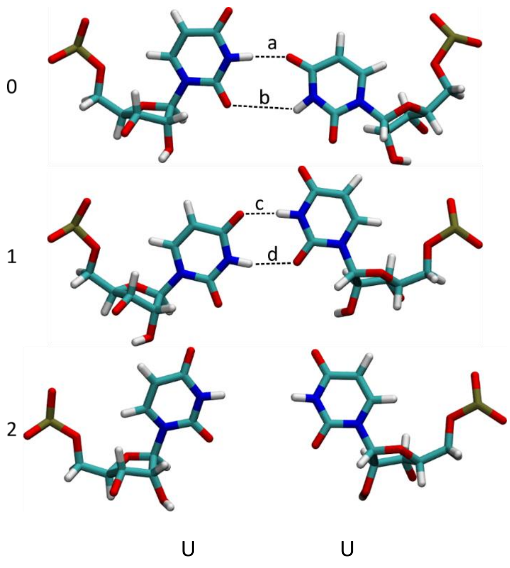

GG pair:

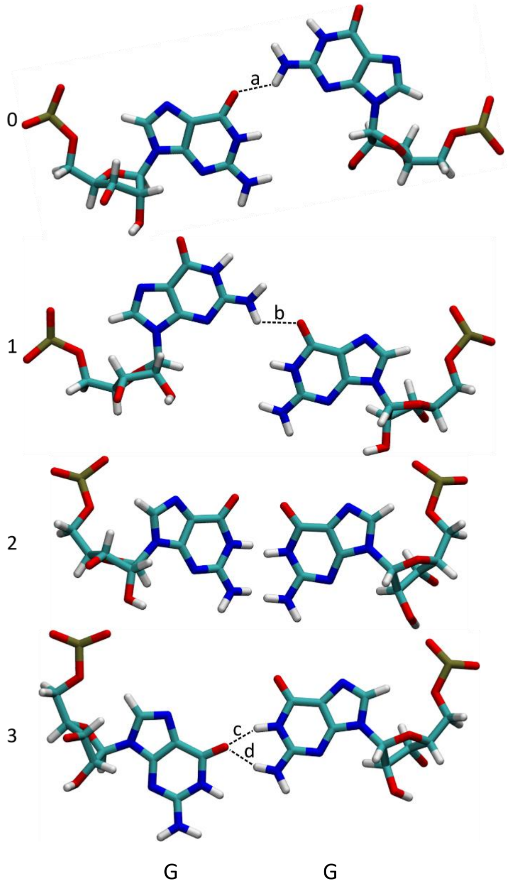

CC pair:

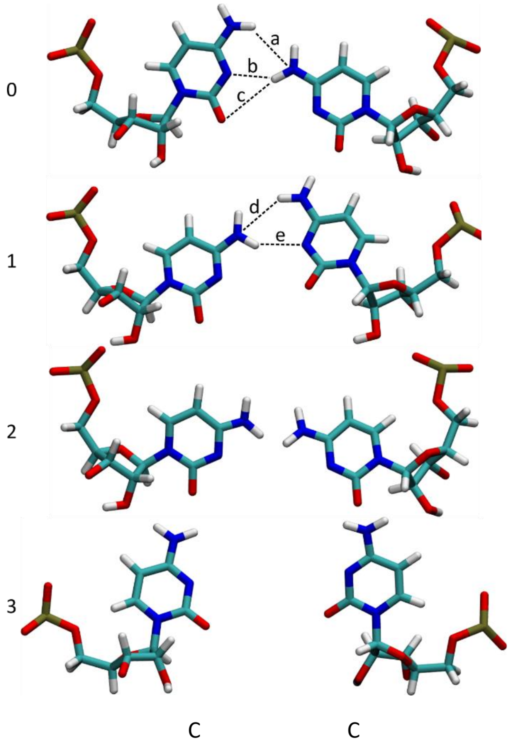

Percentage of time that Hydrogen bonds remain formed between each base pair variant on each precursor. Letters indicating different hydrogen bonds (H b) are indicated in the base pair conformations depicted above. The pair corresponding to wt variant on each precursor is highlighted with a purple border

.

H b	*CG-172a*	*GC-172a*	*CG-164c*	*GC-164c*
a	70,24%	66,24%	72,54%	67,48%
b	71,66%	72,18%	78,02%	75,64%
c	86,88%	88,64%	88,06%	85,72%
H b	AU-172a	UA-172a	AU-164c	UA-164c
a	49,12%	59,38%	60,70%	52,10%
b	65,40%	62,98%	62,44%	71,74%
H b	GU-172a	UG-172a	GU-164c	UG-164c
a	63,30%	62,34%	71,66%	76,58%
b	80,02%	79,14%	71,86%	80,86%
c	3,82%	4,40%	4,22%	1,90%
H b	GA-172a	AG-172a	GA-164c	AG-164c
a	49,94%	41,84%	54,64%	48,88%
b	43,22%	36,72%	34,82%	40,94%
c	2,76%	-	-	-
H b	CA-172a	AC-172a	CA-164c	AC-164c
a	1,62%	36,72%	1,24%	2,50%
b	38,78%	41,20%	29,36%	22,68%
c	1,18%	1,64%	-	6,08%
d	7,56%	-	1,68%	1,96%
e	-	1,90%	-	-
f	-	-	41,64%	-
H b	CU-172a	UC-172a	CU-164c	UC-164c
a	54,40%	38,12%	36,98%	5,95%
b	-	-	1,38%	-
c	-	4,42	5,2%	3,36%
d	-	1,66%	-	67,84%

H b	*AA-172a*	*AA-164c*
a	6,08%	-
b	5,92%	-
c	-	52,52%
d	-	22,00%
H b	GG-172a	GG-164c
a	17,26%	27,76%
b	1,60%	-
c	-	33,30%
d	-	31,40%
H b	UU-172a	UU-164c
a	33,82%	17,24%
b	2,64%	4,04%
c	18,34%	53,22%
d	3,62%	22,92%
H b	CC-172a	CC-164c
a	1,96%	3,48%
b	4,68%	9,80%
c	-	3,40%
d	1,16%	-
e	4,32%	1,96%

Clustering of conformations visited through the simulation of *MIR172A* variants. Above each graph the nucleotide pair is indicated. Note that there is not a graph for wobbles or Watson-Crick pairs because those always remains in the same conformation.

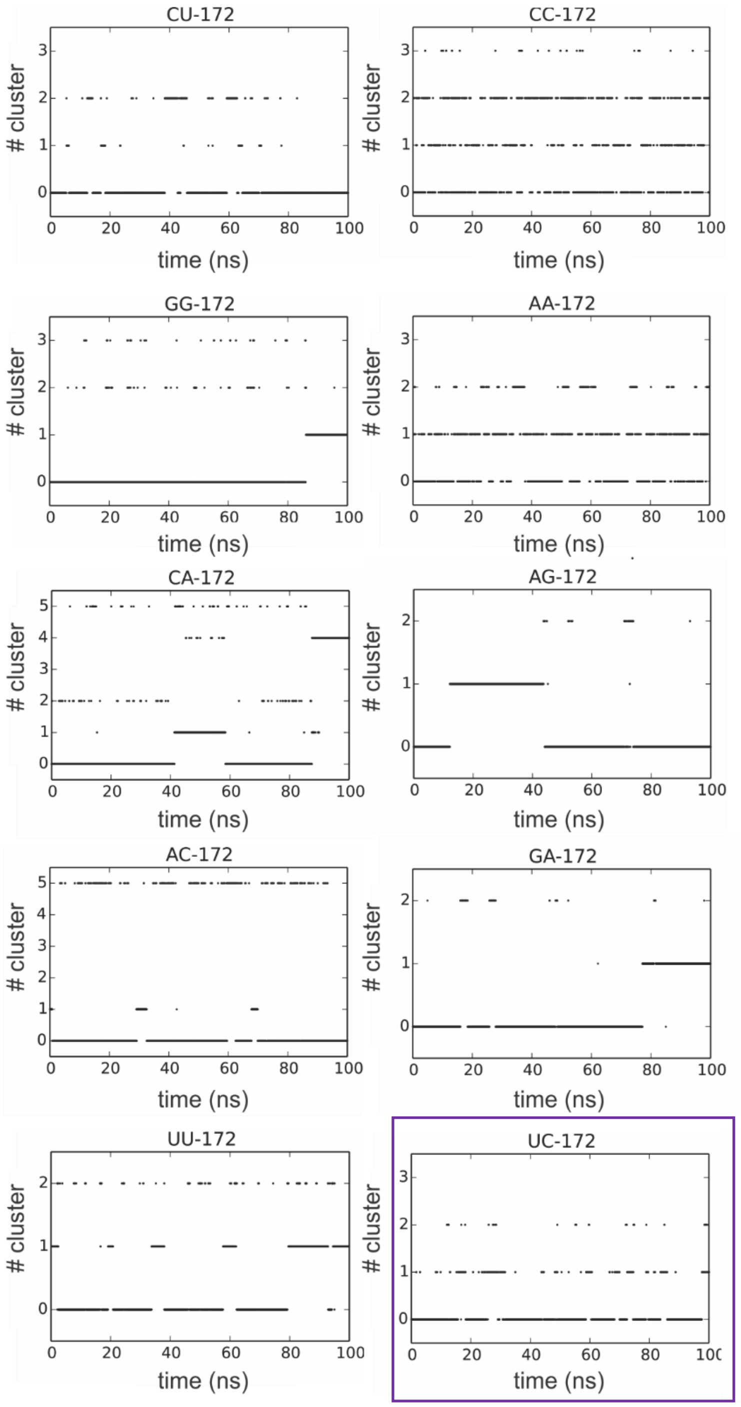

Clustering of conformations visited through the simulation of *MIR164C* variants. Above each graph the nucleotide pair is indicated. Note that there is not a graph for wobbles or Watson-Crick pairs because those always remains in the same conformation. The wt variant has a G-C pair.

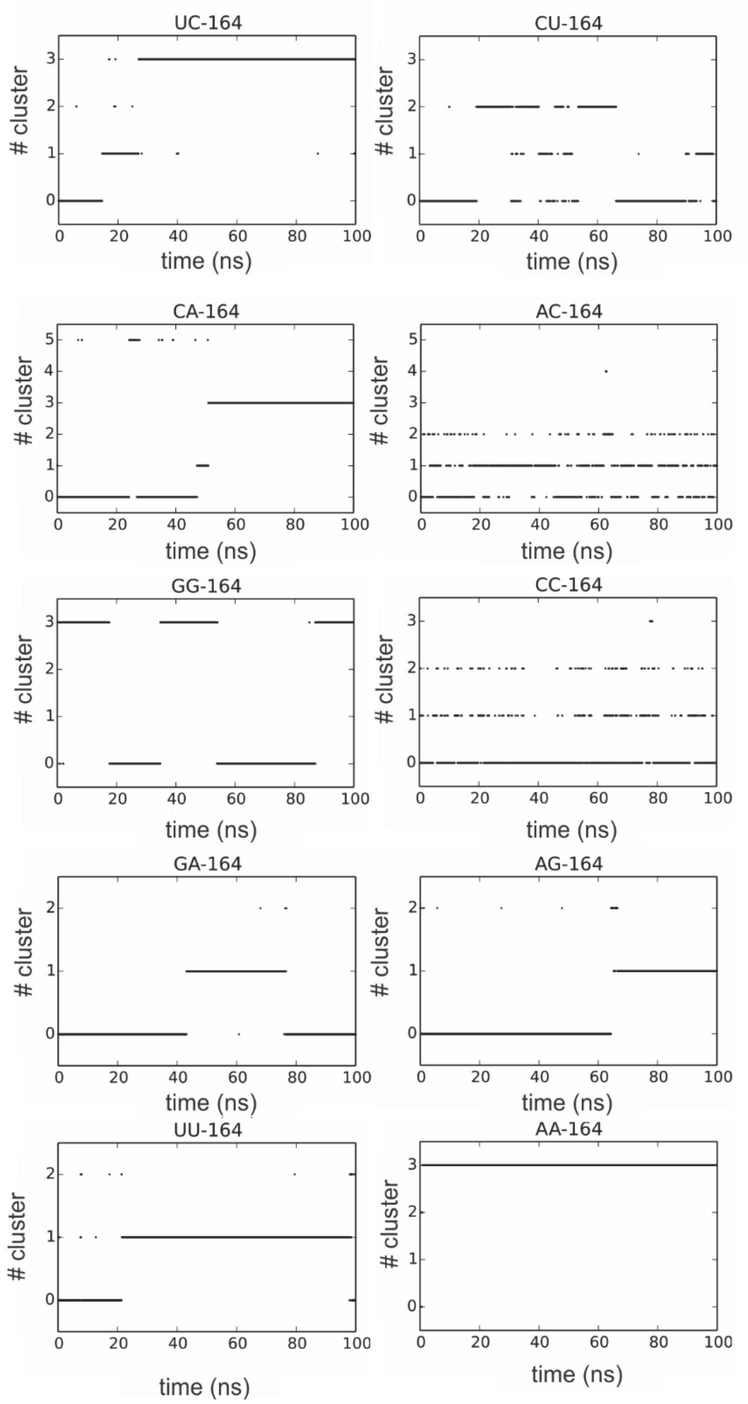

Supplement: Supplementary file 4 — Supplementary Data 3 [file 41467_2020_19129_MOESM4_ESM.pdf]
